# Taming the late Quaternary phylogeography of the Eurasiatic wild ass through ancient and modern DNA

**DOI:** 10.1371/journal.pone.0174216

**Published:** 2017-04-19

**Authors:** E. Andrew Bennett, Sophie Champlot, Joris Peters, Benjamin S. Arbuckle, Silvia Guimaraes, Mélanie Pruvost, Shirli Bar-David, Simon J. M. Davis, Mathieu Gautier, Petra Kaczensky, Ralph Kuehn, Marjan Mashkour, Arturo Morales-Muñiz, Erich Pucher, Jean-François Tournepiche, Hans-Peter Uerpmann, Adrian Bălăşescu, Mietje Germonpré, Can Y. Gündem, Mahmoud-Reza Hemami, Pierre-Elie Moullé, Aliye Ötzan, Margarete Uerpmann, Chris Walzer, Thierry Grange, Eva-Maria Geigl

**Affiliations:** 1 Institut Jacques Monod, UMR7592, CNRS-Université Paris Diderot, Paris, France; 2 Institute of Palaeoanatomy, Domestication Research and the History of Veterinary Medicine, Ludwig-Maximilian University, Munich, Germany; 3 SNSB, Bavarian State Collection of Anthropology and Palaeoanatomy, München, Germany; 4 Department of Anthropology, University of North Carolina at Chapel Hill, Chapel Hill, North Carolina, United States of America; 5 Mitrani Department of Desert Ecology, Jacob Blaustein Institutes for Desert Research, Ben-Gurion University of the Negev, Sede Boqer Campus, Midreshet Ben-Gurion, Israel; 6 Laboratório de Arqueociências, (DGPC), Ajuda, Lisbon, Portugal; 7 Centre de Biologie pour la Gestion des Populations CBGP, Montferrier-sur-Lez, France; 8 Research Institute of Wildlife Ecology, University of Veterinary Medicine, Vienna, Austria; 9 Technische Universität München, AG Molekulare Zoologie/Lehrstuhl für Zoologie, Freising, Germany; 10 CNRS and Muséum national d'Histoire naturelle, UMR 7209, Archéozoologie, archéobotanique: sociétés, pratiques et environnements, Département Ecologie et Gestion de la Biodiversité, Paris, France; 11 Laboratory of Archaeozoology, Dept. Biologia, Universidad Autonoma de Madrid, Madrid, Spain; 12 Naturhistorisches Museum, Vienna, Austria; 13 Musée d'Angoulême, Angoulême, France; 14 Eberhard-Karls-Universität Tübingen, Institut für Ur- und Frühgeschichte und Archäologie des Mittelalters, Abteilung Ältere Urgeschichte und Quartärökologie, Zentrum für Naturwissenschaftliche Archäologie, Tübingen, Germany; 15 National History Museum of Romania, National Centre of Pluridisciplinary Research, Bucureşti, Romania; 16 Royal Belgian Institute of Natural Sciences, Earth and History of Life, Brussels, Belgium; 17 Department of Natural Resources, Isfahan University of Technology, Isfahan, Iran; 18 Musée de Préhistoire Régionale, Menton, France; 19 Ankara Üniversitesi Dil ve Tarih-Coğrafya Fakültesi, Ankara, Turkey; Senckenberg am Meer Deutsches Zentrum fur Marine Biodiversitatsforschung, GERMANY

## Abstract

Taxonomic over-splitting of extinct or endangered taxa, due to an incomplete knowledge of both skeletal morphological variability and the geographical ranges of past populations, continues to confuse the link between isolated extant populations and their ancestors. This is particularly problematic with the genus *Equus*. To more reliably determine the evolution and phylogeographic history of the endangered Asiatic wild ass, we studied the genetic diversity and inter-relationships of both extinct and extant populations over the last 100,000 years, including samples throughout its previous range from Western Europe to Southwest and East Asia. Using 229 bp of the mitochondrial hypervariable region, an approach which allowed the inclusion of information from extremely poorly preserved ancient samples, we classify all non-African wild asses into eleven clades that show a clear phylogeographic structure revealing their phylogenetic history. This study places the extinct European wild ass, *E*. *hydruntinus*, the phylogeny of which has been debated since the end of the 19^th^ century, into its phylogenetic context within the Asiatic wild asses and reveals recent mitochondrial introgression between populations currently regarded as separate species. The phylogeographic organization of clades resulting from these efforts can be used not only to improve future taxonomic determination of a poorly characterized group of equids, but also to identify historic ranges, interbreeding events between various populations, and the impact of ancient climatic changes. In addition, appropriately placing extant relict populations into a broader phylogeographic and genetic context can better inform ongoing conservation strategies for this highly-endangered species.

## Ethics statement

**Fig 1:**
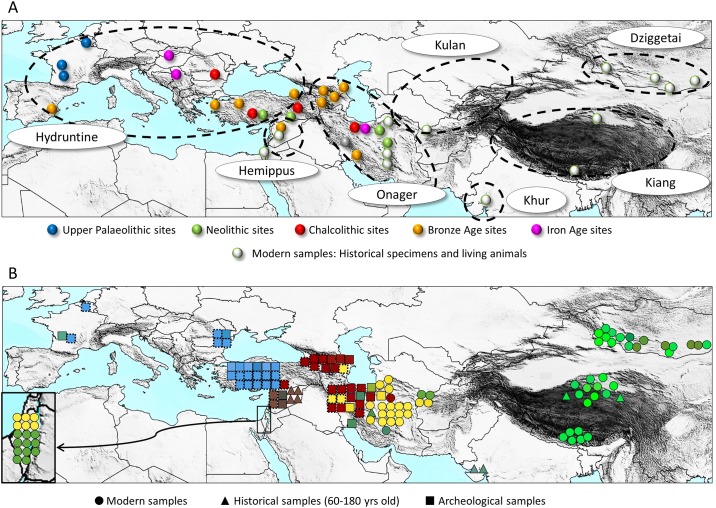
Map representing the origin of the samples and the results of the landscape genetics sPCA analyses. **A)** Origin of the samples. The color code indicates the dates of the sites from which the samples originated. The dotted lines indicate the past range of the various hemione populations. **B)** Results from a spatial principal component analysis (sPCA) performed as described in the Material & Methods. The sPCA values of each individual were displayed as a single color by converting each of the three principal scores into a color channel (red, green, and blue for 1^st^, 2^nd^ and 3^rd^ principal components respectively, see Materials & methods). To legibly display all samples analyzed, each individual sequence was placed as closely as possible to its original geographical location while avoiding overlaps. Additional samples for which only partial sequences were obtained but which contained enough information to allow unambiguous assignment to a specific clade are represented using a dotted outer line. Since they did not contain enough sequence information to be used in the sPCA, they are given the color of a representative member of the clade. The specimens from the Hai-Bar Yotveta reserve in Israel that descended from hemiones captured in Turkmenistan and Iran are represented in the magnified box on the lower left side of the map connected by an arrow to their original location.

No animals were used in the present study. Samples used in this study and their archeological contexts are described in the Supporting Information and listed in Table A in S1 File. Briefly, we analyzed 189 archaeological bone and teeth specimens that had been assigned osteologically to *E*. *hemionus sp*., *E*. *hydruntinus* or *E*. *kiang* and were dated between 3,500 and 100,000 years ago. These samples originated from 49 archaeological sites in ten European and six southwest Asian countries (Fig 1A and Table A in S1 File). In addition, we analyzed 11 historical museum specimens (between 60 and 180 years old) of onagers, hemippi, khurs and kiangs and 53 present-day samples, 94% of which originated from wild individuals, coming from the Gobi Desert and protected nature reserves in Iran and Israel. The samples from the Negev desert in Israel came from the Israel Nature and Park Authority (INPA) breeding core and had been collected there between 1989 and 1991 as part of the INPA management activities toward reintroduction (e.g., medical treatment, translocations between enclosures): http://www.parks.org.il/sites/English/parksandreserves/haibaryotvata/Pages/default.aspx.

## Introduction

The Asiatic wild ass (*Equus hemionus*), once widely distributed over a vast geographical area, is witnessing a dramatic range reduction leaving nearly all of the remaining but isolated populations endangered. In many high-altitude plains or deserts of Asia, these arid-adapted and cold-tolerant animals have long been the largest and most widespread herbivore taxon, and their disappearance threatens to eliminate a major ecological agent from these extreme environments. In contrast to the caballoids, or horses, Asiatic wild asses belong to the stenonids, a group which also includes zebras and the African wild ass *Equus africanus* along with its domestic form *E*. *asinus*. Currently, Asiatic wild asses are subdivided into two species, *Equus kiang*–the kiang of Tibet, and *Equus hemionus* with four living and one extinct subspecies, i.e., *E*. *h*. *hemionus* (also known as *E*. *h*. *luteus*)–the Mongolian kulan or dziggetai, *E*. *h*. *khur*–the Indian khur, *E*. *h*. *kulan*–the Turkmen kulan, *E*. *h*. *onager*–the Iranian or Persian onager, and *E*. *h*. *hemippus*–the extinct Syrian wild ass [1–3] (see Fig 1A for their geographic ranges). The two largest surviving populations, the dziggetais in the Mongolian Gobi Desert [3] and the kiangs of the Tibetan plateau [4], still occur over large parts of their former distribution range. However, increased livestock grazing, fencing, railway and highway construction, and poaching also threaten the future of dziggetais and kiangs. The Iranian onagers, the Turkmen kulans, and the Indian khurs are reduced to small pocket populations with contracted distributions in protected areas located either in endemic centers or in refuge zones in Iran, Turkmenistan and northwest India, respectively [3,5,6].

Understanding the evolution as well as past and present genetic diversity of these species is essential for the design of appropriate conservation strategies [7,8]. Asiatic wild asses, however, are not well characterized genetically. The profound lack of data on the past and recent distribution and population structures of these regionally endangered animals is particularly worrisome at a time so critical for the conservation of Asiatic wild asses. Shrinking population sizes and habitat reduction of species on their way to extinction lead to isolated pocket populations the analyses of which tend to overemphasize their differences. This may result in taxonomic over-splitting. The knowledge of past distributions and diversity assessed using paleontological and paleogenetic approaches helps to minimize this problem and to define and implement adequate conservation biology strategies.

Although palaeontology has accumulated much data concerning equid evolution, long considered a paradigmatic evolutionary model [7], much taxonomic uncertainty remains. Indeed, the classification of ancient equids based on osteomorphometry is ambiguous since modern skeletons used for comparisons represent mosaics of various, restricted combinations of a relatively small number of characteristics [1]. Whole skeletons are required for reliable morphological determination and these are extremely rare in the fossil record [8]. Consequently, knowledge of past morphological diversity within and between equid species is scarce. These features put the presently accepted equid taxonomy on shaky ground and question interpretations about the ancient geographical and temporal distribution of this taxon ([9], and citations therein).

Paleontological approaches have identified a European wild ass, termed *E*. *hydruntinus*, or hydruntine, known only from skeletal remains and prehistoric engravings such as that found in the cave of *Les Trois Frères* in France. The oldest Western European remains that have been attributed to this morphotype are from France and date to around 350,000 years ago [10]. The hydruntine was widespread during the Late Pleistocene, with a geographic distribution from Western Europe to the Volga, Turkey, the Levant and the northern Middle East [11–13]. During the Holocene hydruntine populations declined and were reduced to small patches of their previous range, before eventually becoming extinct [12].

Paleogenetic analyses of the mitochondrial and, very recently, nuclear genomes preserved in equid bones have allowed researchers to revisit equid taxonomy, which has reduced the number of species proposed in paleontological studies [14–18]. These recent paleogenetic studies suggested that the “oversplitting” of earlier palaeontological work was the consequence of an underestimation of the morphological plasticity of equids throughout their ranges and evolutionary history [17]. Indeed, ancient DNA research has the potential to unravel the phylogeographic structure of populations and species, past migrations, gene flow, erosion of past diversity and population fragmentation. By correctly identifying the past geographic distribution of genotypes, it is possible to reconstruct the sequences of such events (e.g., [19,20]).

We studied the mitochondrial lineages of the wild asses from Europe and Asia in archeological, historical and recent samples spanning the last 100,000 years and the area from western Europe to eastern Asia. The ancient DNA (aDNA) results obtained show that during the Upper Pleistocene the distribution of the Asiatic wild ass ranged from western Europe, where it is now extinct, to eastern Asia where it is still found at present. The genetic relationships between these taxa explain why we subsume these populations under the unifying term “Eurasiatic wild ass”. We explored the patterns of the past and present genetic diversity to reconstruct the population structure of the species and its evolution since the Late Pleistocene.

## Materials & methods

Samples used in this study and their archeological contexts are described in the Supporting Information and listed in Table A in S1 File. Briefly, we analyzed 189 archaeological bone and teeth specimens that had been assigned osteologically to *E*. *hemionus sp*., *E*. *hydruntinus* or *E*. *kiang* and were dated between 3,500 and 100,000 years ago. These samples originated from 49 archaeological sites in ten European and six southwest Asian countries (Fig 1A; Table A in S1 File). In addition, we analyzed 11 historical museum specimens (between 60 and 180 years old) of onagers, hemippi, khurs and kiangs and 53 present-day samples, 94% of which originated from wild individuals, coming from the Gobi Desert and protected nature reserves in Iran and Israel.

### Working procedures

Modern and historical specimens were processed in a laboratory of the Jacques Monod Institute (IJM) dedicated to modern, non-amplified DNA analysis, which is physically separated from the ancient DNA facility and post-amplification laboratory, using aDNA procedures. Ancient specimens (those older than 150 years) were processed in the core facility of palaeogenomics of the IJM, a high containment laboratory physically separated from the modern DNA laboratories and dedicated to the analysis of ancient DNA.

Ancient samples were processed in the Core Facility of Palaeogenetics at the IJM, Paris (http://www.ijm.fr/ijm/plates-formes/pole-paleogenomique/). This highly contained pressurized laboratory dedicated to aDNA analysis is isolated on a floor of the institute where no other laboratories of molecular biology are located. It consists of an airlock chamber and three different laboratory rooms, each subjected to a positive air pressure gradient, dedicated to the specific steps of the experimental procedures, (i) sample preparation, (ii) DNA extraction and purification, (iii) PCR set-up. Within the laboratory, each experimental step is carried out in a working station or flow hood. The working stations and equipment are cleaned with bleach (3.5% hypochlorite solution) or RNase away^®^ (Molecular Bio-Products, USA) and UV irradiated at short distance between each experiment to ensure efficient decontamination [21]. To minimize contamination with exogenous DNA and maximize the aDNA yield, low retention microtubes (Axygen, Union City, USA) and extra-long filtered pipette tips were used for extraction and PCR preparation. Experimenters entered the laboratory only after having removed their street clothes and replaced them with lab clothing washed with bleach. All-body protection was worn during each step of pre-PCR work consisting of a disposable protective suit (one specific to each laboratory room), two pairs of gloves, shoe covers and a facemask. Purification was carried out in a flow hood and pipetting of the PCRs in an enclosed PCR workstation (COY, MI, USA), both cleaned with bleach and UV-irradiated at a short distance after each experiment. Capillaries containing the reagent mixtures for the PCR and the fossil extracts were closed in the PCR workstation of the PCR-preparation room in the contained laboratory prior to transfer to the PCR machines on a different floor of the building. The handling of post-PCR products was exclusively performed in a dedicated laboratory that is physically separated from the pre-PCR laboratory and the room containing the PCR machines. Protective disposable clothing and shoe covers are also worn when entering the post-PCR laboratory and removed when exiting.

### DNA extraction

#### Samples from extant specimens

Genomic DNA isolation from blood, muscle and lung tissue was performed with the NucleoSpin^®^-Tissue Kit (Macherey-Nagel, Dueren, Germany). Feces were processed using the QIAamp DNA Stool Mini Kit (Qiagen, Hilden, Germany). Skin, hoof, and hair samples were digested with a hair lysis buffer containing 100 mM Tris-HCl pH 8.0, 100 mM NaCl, 40 mM DTT, 3 mM CaCl_2_, 2% N-lauryl sarcosyl, 250 μg/mL proteinase K (modified after [22]), and DNA was subsequently extracted with the NucleoSpin-Tissue Kit (Macherey-Nagel).

Blood samples of individuals from the Hai-Bar Yotveta breeding core, Israel, had been collected between 1989 and 1991 as part of the Israel Nature and Park Authority (INPA) breeding core management activities toward reintroduction [23].

#### Samples from museum specimens

Historical bone and teeth samples were extracted using ancient DNA methods (see below). Sinew and cartilage samples were crushed in liquid nitrogen in a mortar and DNA was purified using the QIAamp DNA Stool Minikit (Qiagen, Hilden, Germany).

#### Ancient samples

In the high containment laboratory, bone and tooth surfaces were removed with a sterilized razor blade and, depending on the individual bone characteristics, either drilled with a heat-sterilized bit using either a Dremel 4000 or Dremel Fortiflex (Dremel Europe, The Netherlands), or cut into fragments and powdered using a freezer mill (SPEX CertiPrep 6750, USA), or powdered manually using a razor blade. Bone powder (34–1896 mg) was incubated in 1–10 mL extraction buffer (0.5 M EDTA, 0.25 M PO_4_^3-^, pH 8.0, 1% beta-mercaptoethanol) for 48–70 hours at 37°C with agitation. Some samples required changes of extraction buffer to increase digestion of bone powder. Hair and hoof samples were added to a hair lysis buffer (as above) and incubated 4–24 hours at 50°C, shaken with 300 RPM. Solutions were then pelleted and the supernatant was purified using a QIAquick Gel extraction kit (Qiagen, Hilden, Germany) with protocol modifications [24].

For external reproduction of the results, some ancient samples (S1, D4, K2, ASE24, ASE25, ASE382, ASE383, ASE384) were analyzed by M.P. in the laboratory of the Humboldt-University Berlin, Landwirtschaftlich-Gärtnerische Fakultät, Molekularbiologisches Zentrum Ostbau, Berlin, Germany: Approximately 250 mg of bone material was used for each extraction. External surfaces of bones were removed by abrasion to minimize environmental contaminations. Each sample was ground to powder with a freezer mill and incubated in 0.45 M EDTA (pH 8.0) and 0.25 mg/ml Proteinase K overnight at room temperature under rotation. After centrifugation for 5 min at 4,000 rpm in a Universal 320 centrifuge (Hettich), DNA was purified from the supernatant using a silica based method as previously described [25,26].

### DNA amplification and sequencing

Primers for archeological samples were designed to amplify a total of up to 328 base pairs of the hypervariable region of wild ass mitogenome using short, overlapping fragments. A list of primers used and product sizes is given in Table B in S1 File.

Purified DNA was amplified by qPCR, the extract making up 5–20% total reaction volume (10–20μl). Inhibition characteristics were determined for failed samples indicating possible inhibition. For evaluation of the inhibition, three parameters were taken into consideration: (i) delay of the threshold value Ct (crossing point at threshold), (ii) the kinetics of the synthesis of the PCR product, and (iii) the efficiency of the PCR. The quantity of aDNA extract amplified by PCR was adjusted according to the results of the inhibition tests to minimize interference of the inhibitors with the PCR.

QPCR was performed using Lightcycler 1.5 or Lightcycler 2 (Roche Applied Science, Mannheim, Germany). To protect against cross-contamination, the UQPCR method was used [21,27] in which uracil was substituted for thymidine for all PCRs, and incubation with uracil N-glycosylase (UNG, extracted from *G*. *morhua*; ArcticZymes, Trømsø, Norway) was performed prior to each reaction. Mock extraction blanks were performed with each extraction and amplified to control for contamination. QPCR reactions varied slightly depending on the sample, but a typical reaction included 1.77 μL of LC FastStart DNA MasterPLUS mix 1b and 0.23 μL of either FastStart DNA MasterPLUS mix 1a or FastStart Taq (Roche Applied Science, Mannheim, Germany), 1 μM of each primer (Sigma Aldrich, St. Louis, USA) and 1U of UNG and water to 10 μL total volume. QPCR programs consisted of a UNG incubation step at 37°C for 15 minutes, followed by polymerase activation at 95°C for 8 minutes, 60 to 80 two-step cycles of denaturation at 95°C for 10 seconds, primer annealing and extension at 58–62°C for 40–60 seconds, and finally a temperature increase of 0.1°C/1 second from the annealing temperature to 95°C with continuous fluorescence measurement to generate melt-curves of the products.

For each ancient sample, at least two independent extractions were performed, separated in time. Each extract was amplified with several primers and each PCR product was obtained at least twice. An average of one non-template control (NTC) was run for every 6.6 samples (including mock extracts). No DNA was amplified in either NTCs or mock extracts, indicating no detectable exogenous equid DNA was introduced during sample or PCR preparation, or was present in reagents.

Products were purified using QIAquick PCR purification kit (Qiagen, Hilden, Germany) and both strands were sequenced by capillary electrophoresis at Eurofins/MWG Operon (Ebersberg, Germany) using an ABI 3730xl DNA Analyzer (Life Technologies). Sanger electropherograms were visually inspected and sequences manually curated, assembled and aligned using the Geneious software suite [28].

PCR analyses of the samples replicated at Humboldt University in Berlin were performed using an overlapping set of primers as described [29]. Using these primers, 713 bp (15.468–16.181 nps) were amplified with a two-step multiplex PCR. Overlapping PCR products, including primers, varied in length between 108 bp and 178 bp. PCR conditions were as described [29]. Multiple negative extraction controls and negative PCR controls were performed. Amplification products were visualized on agarose gel and sequenced on an ABI PRISM 3730 capillary sequencer using the BigDye Terminator v3.1 cycle sequencing kit (Applied Biosystems). The resulting sequences were aligned with those obtained in Paris and found to be identical over their overlapping lengths.

DNA amplification from blood samples originating from individuals at the Hai-Bar Yotveta breeding core, Israel, was performed at the Jacob Blaustein Institutes for Desert Research, Ben-Gurion University of the Negev, Israel. The 20 μl reactions contained 20 ng DNA, 0.25 μM of each of the primers EA1 and EA63, 500 μM dNTPs, 2 mM MgCl2 and 1 U Taq DNA polymerase (Hylabs, Rehovot, Israel). PCR conditions were: 5 minutes at 95°C followed by 40 cycles of 10 seconds at 95°C, 30 seconds at 62°C and 15 seconds at 68°C, and a final elongation step of 10 minutes at 72°C. PCR products were purified using Exosap IT (USB, Cleveland, OH), and sequenced by ABI 3730XL DNA analyzer (Applied Biosystems).

### Phylogenetic and phylogeographic analyses

Phylogenetic analyses were performed on the sequences obtained from 26 archeological samples, 9 museum specimens, 53 modern samples, and supplemented with 33 sequences from public databases.

#### Spatial genetics (sPCA)

To investigate the potential relationship between geographic distances and genetic variation, a spatial principal component analysis (sPCA) was carried out on a 229-bp-long sequence of 102 archeological, museum and modern georeferenced samples (Table D in S1 File) using the R packages ade4 and adegenet [30–32].

While with PCA the optimization criterion only deals with genetic variance, sPCA aims at finding independent synthetic variables that maximize the product of the genetic variance and spatial autocorrelation measured by Moran's *I* [33]. This is accomplished by the eigenvalue decomposition among individuals via a neighboring graph (in this study a Delaunay triangulation was chosen) connecting the individuals on the geographical map to model spatial structure. Resulting eigenvalues can be either positive or negative reflecting respectively either a global or local spatial pattern. A global structure implies that each sampling location is genetically closer to neighbors than randomly chosen locations. Conversely, a stronger genetic differentiation among neighbors than among random pairs of entities characterizes a local structure. To evaluate the consistency of the detected geographical structures versus a random spatial distribution of the observed genetic variance, a Monte-Carlo based test was applied [32]. This test simulated a random distribution of the genetic variability (null hypothesis) on the Delaunay triangulation connection network and calculated a *p*-value depending on the dataset. The simulated distribution represents the correlation of the randomized genetic variables with the vectors of the Moran’s *I* predicting for the global or local structure. If the value associated to the observed pattern is higher than the *p*-value, the spatial distribution of the genetic variance is not random and the null hypothesis can be rejected. We applied the test with 10,000 iterations.

For representing the sPCA scores of each individual we used the colorplot function implemented in the R-software package adegenet [31]. This function visualizes up to three scores at the same time by translating each score into a color channel (RGB for red, green, and blue). The values obtained were expressed as a single color under the RGB system as a parameter of the individual genetic constitution and projected on a world map. The sPCA scores, RGB and GPS values for each georeferenced individual shown in the phylogenetic tree are listed in Table D in S1 File. The sPCA scores correspond to the first three axes, as well as the last (36^th^) one. The RGB values are derived from the first three axes of the analysis. The graphical representation of the various results of the sPCA analysis are represented in Figs C-G in S2 File.

**Fig 2:**
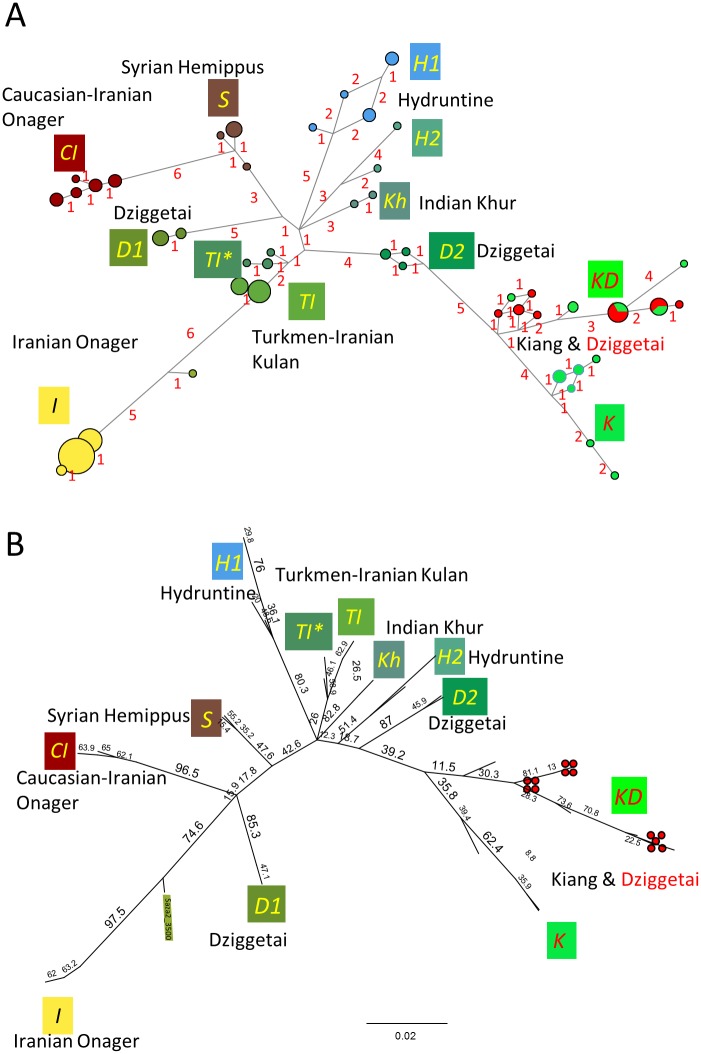
Median joining network analysis (A) and maximum likelihood analysis based on PHYML (B). The names of the deduced clades are indicated in italics. The colors of the box with the clade name follow the same convention as in the sPCA analysis displayed in Fig 1.

**Fig 3:**
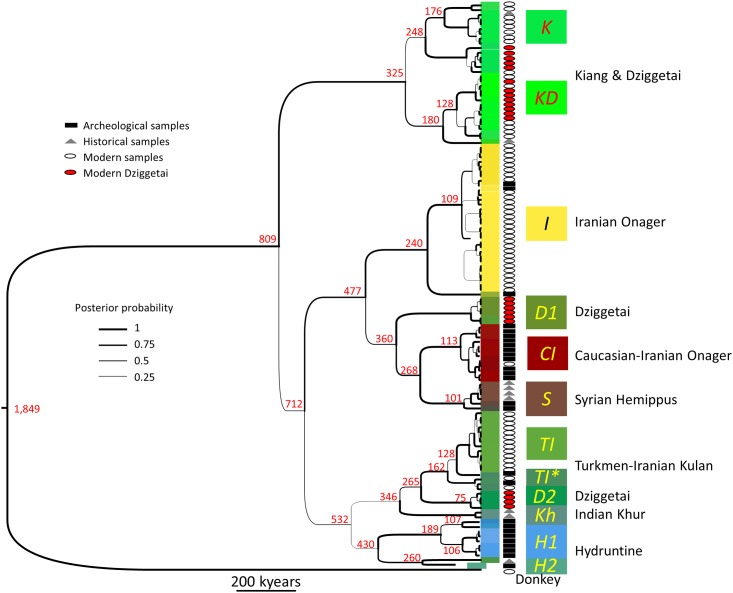
Phylogenetic tree of the mitochondrial control region of the Eurasiatic wild ass constructed through BEAST analysis. The corresponding *E*. *asinus* DNA region was used as an outgroup. The estimated median height of the nodes is indicated in red, in kiloyears (kyears), and the thickness of the lines is proportional to the posterior clade probability (the scale is represented). The mean substitution rate averaged across the whole tree is 8.5 E-8 substitutions per site per million years (95% HPD interval: 2.1–18.8 E-8). The colors of the box surrounding each individual sequence follow the same convention as in the sPCA analysis displayed in Fig 1. The names of the deduced clades are indicated in italics. The symbols following each sequence name indicated the origin of the sample (Square: Archeological; Triangle: Historical; Circle: Modern), and the red circles indicate the modern dziggetais (see text). For an enlarged representation of the tree containing the names of the sequences, the 95% HPD of the node height values, the posterior probabilities of the nodes and their bootstrap values by ML analysis, see Fig H in S2 File.

Each successfully analyzed sample was placed on the map as closely as possible to its geographical location and colored using the attributed RGB values representing the sPCA results (Fig 1B). The color obtained from the sPCA scores was also manually assigned to each sequence on the median joining network (Fig 2A), the maximum likelihood tree (Fig 2B) and the Beast tree (Fig 3).

#### Median joining network

The median joining network of mitochondrial HVR (Fig 2A) was constructed using the 229-bp-long sequences and the Network 4.6.0.0 program with a maximum parsimony post processing step (http://www.fluxus-engineering.com) [34].

#### Maximum likelihood analysis

**Fig 4:**
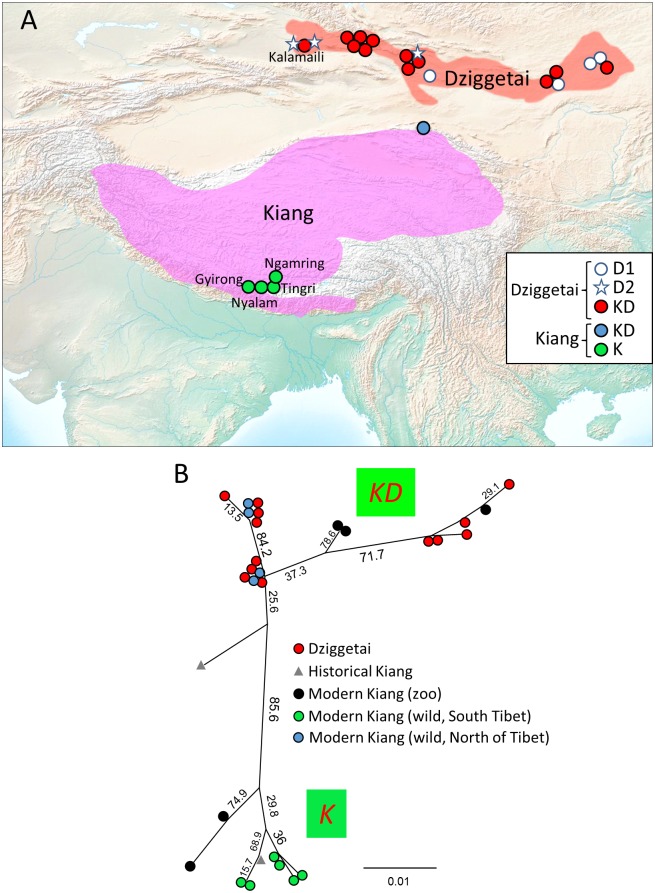
Geographic distribution of the analyzed kiangs and dziggetais and maximum likelihood phylogeny of the *K/KD* clades. **(A)** The map indicates the current areas of distribution of kiangs (pink) and dziggetais (orange) as determined by IUCN [3,4], as well as the location of the collected wild samples. Symbols and colors schemes are used to represent the various samples and populations. The southern Tibetan reserves populated by kiangs (Nyalam, Gyirong, Ngamring and Tingri; [54]), are represented by green circles, whereas the blue circles indicate the kiang samples collected north of Tibet [49]. The dziggetai samples analyzed herein, as well as those from the Kalamaili natural reserve [54], whose mitogenomes belong to the *D1* and *D2* clades are indicated by white circles and stars, respectively, whereas those belonging to the *KD* clade are represented by red circles. Differences in the East-West distribution of the Dziggetai *D1* and *D2* clades are observed, although it is unclear how these correlate with the East-West distributions observed for microsatellite markers in [76]. **(B)** The ML phylogeny of the kiang and dziggetai sequences of the *KD* and *K* clades was performed with PHYML [35] using the full contiguous 295-bp-long HVR sequence. The bootstrap values of the nodes are indicated (1000 bootstraps). The red, green and blue circles indicate the geolocalized dziggetais, northern Tibetan kiangs, southern Tibetan kiangs, respectively, as in panel A. The modern kiang from zoos are represented with black circles whereas the historical kiang specimens are represented with a grey triangle. See also Table E in S1 File for the summary statistics of the kiang and dzigettai populations analyzed here.

Maximum likelihood (ML) analyses were computed using either the 229-bp-long HVR sequences of both ancient and modern samples (Fig 2B) or the contiguous 295-bp-long HVR sequence to analyze both historical and present-day kiangs and dziggetais (Fig 4B) with the software PHYML 3.0 [35,36]. Since *E*. *hemionus* mitochondrial sequences harbor a specific 28-bp-deletion that distinguishes them from all other equid sequences, and since the phylogenetic information of a deletion is not properly accounted for by substitution models, we performed ML analyses of the hypervariable regions using only sequences with this deletion. Based on model comparison criteria performed with jModelTest [37], we considered a TN93 model for the nucleotide substitution model [38] and a gamma-distributed rate of variation among sites (+G) with four rate categories (i.e., TN93+G model). We used 1000 bootstraps to estimate node robustness. We also used RaXML 8.2.3 [39] with the bootstrap convergence criterion autoMRE, which performed about a 1000 bootstraps, to determine the ML bootstrap support of the nodes of the maximum clade credibility tree of the Bayesian analysis presented in Fig H in S2 File.

#### BEAST analysis

Phylogenetic analyses were conducted under the Bayesian framework implemented in the program BEAST v. 1.7.5, which allows estimation of mutation and population history parameters simultaneously from temporally spaced sequence data [40]. We used the 229-bp-long HVR sequence and the corresponding mitochondrial reference sequence of the domesticated donkey as an outgroup, and enforced monophyly to all sequences harboring the specific 28-bp-deletion. We used a strict molecular clock with a uniform prior over the (10^−12^, 10^−6^) interval for the substitution rate measured in substitutions per site and per year. Based on model comparisons criteria performed with jModelTest [37], we further considered a TN93 model for the nucleotide substitution model [38] and a gamma-distributed rate of variation among sites (+G) with four rate categories (i.e., TN93+G model). Default priors were used for the seven parameters (alpha, kappa1, kappa2 and the four nucleotide frequencies) of the TN93+G nucleotide substitution model. Finally, a standard coalescent model was considered for the tree prior with a constant population size [41,42] and a log-normal prior distribution for the tree prior height with a mean of 13.5, a standard deviation of 0.843 and an offset of 1,500,000 years. This prior distribution is centered on 2,230,000 years, which integrate the various assumed and estimated divergence times between the ancestors of African and Asiatic wild asses [17,43,44], but within a relatively large range of possible values as the 95% credibility interval covers 1,640,000 to 5,300,000 years. The simplifying assumption of a constant population size overall was made to avoid overfitting of the data with too many parameters because populations must have expanded and contracted locally in a complex manner given the very wide spatial and temporal ranges considered in this study.

To estimate the posterior distribution of each parameter of interest, we used the Markov Chain Monte Carlo algorithm implemented in the BEAST software. We ran ten independent chains with initial values sampled as described above and an input UPGMA tree constructed using a Juke-Cantor distance matrix. Each of these chains was run for 10,000,000 iterations and for each parameter of interest, 4,500 samples (one every 2,000 generated ones) were drawn after discarding a 10% burn-in period. The BEAST output was analyzed with the software TRACER v. 1.5.0 [45]. Visual inspection of the traces and the estimated posterior distributions suggested that each MCMC had converged on its stationary distribution. In particular, effective sample size (ESS) values varied from around 300 to around 3,000 (most being over 600). Using Logcombiner, we further combined all the results from the 10 independent chains leading to combined ESSs ranging from 3,500 to 34,000. The maximum clade credibility tree with the median height of the nodes was finally calculated using TreeAnnotator v. 1.7.5 and visualized using FigTree v.1.4.0 http://tree.bio.ed.ac.uk/software/figtree/webcite.

#### Summary statistics

The various summary statistics were computed using DNASP v5.1 [46] and Arlequin v3.5.1.3 [47] and are presented in Tables 1–3 using the 229-bp-long HVR sequences and in Table E in S1 File using the 295-bp-long HVR sequence of the historical and present-day kiangs and dziggetais.

**Table 1:** Population pairwise F_st_ values. Population pairwise F_st_ values: Distance method pairwise difference.

	Gobi	Tibet	Iran (ancient)	Iran-Turkmenistan (modern)	Caucasus	Anatolia-Balkans	Syria
Gobi	**0**	0.00901 ± 0.0091	0.00 ± 0.00	0.00 ± 0.00	0.00 ± 0.00	0.00 ± 0.00	0.00 ± 0.00
Tibet	**0.14212**	**0**	0.00 ± 0.00	0.00 ± 0.00	0.00 ± 0.00	0.00 ± 0.00	0.00 ± 0.00
Iran (ancient)	**0.34288**	**0.52891**	**0**	0.00 ± 0.00	0.08108 ± 0.0212*	0.00 ± 0.00	0.00 ± 0.00
Iran-Turkmenistan (modern)	**0.42786**	**0.54144**	**0.29414**	**0**	0.00 ± 0.00	0.00 ± 0.00	0.00 ± 0.00
Caucasus	**0.54529**	**0.73930**	0.10069*	**0.57956**	**0**	0.00 ± 0.00	0.00901 ± 0.0091
Anatolia-Balkans (with Artenac)	**0.42281**	**0.60104**	**0.50186**	**0.55531**	**0.79964**	**0**	
Syria	**0.45511**	**0.68097**	**0.33034**	**0.55005**	**0.82065**	**0.77230**	**0**

F_st_ values are indicated in bold letters in the lower diagonal, whereas the F_st_ P values (number of permutations: 110) are indicated in the upper diagonal.

The F_st_ value marked with a * is not significant (Pval>0.05). For Iran, sequences from ancient and modern specimens are treated separately, as indicated.

**Table 2:** Pairwise distance between population.

	Gobi	Tibet	Iran (ancient)	Iran-Turkmenistan (modern)	Caucasus	Anatolia-Balkans	Syria	India
Gobi		*0*.*00618*	*0*.*02551*	*0*.*02678*	*0*.*04816*	*0*.*02288*	*0*.*03182*	*0*.*03025*
Tibet	0.03861		*0*.*04194*	*0*.*03585*	*0*.*06341*	*0*.*03309*	*0*.*04793*	*0*.*04369*
Iran (ancient)	0.06694	0.07538		*0*.*01501*	*0*.*00532*	*0*.*03328*	*0*.*01822*	*0*.*03200*
Iran-Turkmenistan (modern)	0.06071	0.06179	0.04995		*0*.*04163*	*0*.*01771*	*0*.*03648*	*0*.*04013*
Caucasus	0.07234	0.08330	0.03050	0.05932		*0*.*05687*	*0*.*03012*	*0*.*05241*
Anatolia-Balkans (with Artenac)	0.05564	0.05785	0.06705	0.05806	0.07338		*0*.*04230*	*0*.*02289*
Syria	0.05467	0.06279	0.04208	0.05283	0.03672	0.05748		*0*.*03632*
India	0.05265	0.05809	0.05679	0.05604	0.05857	0.03762	0.04115	

The lower half of the diagonal of the table represents the pairwise nucleotide divergence (average number of nucleotide substitution per site) with Jukes and Cantor correction, Dxy(JC), whereas the upper half (italicized) represents the net pairwise nucleotide divergence (net average number of nucleotide substitution per site) with Jukes and Cantor correction, Da(JC). Analyses were conducted using DNAsp [46].

**Table 3:** Measurements of intrapopulation diversity.

Population	Nind	Nhap	Haplotype diversity	Nucleotide diversity, Pi	Theta(k)	Theta(S)	Theta(Pi)	Theta (per site)
Gobi	23	12	0.93 ± 0.03	0.041 ± 0.021	9.41	6.23± 2.36	9.40 ± 5.00	0.028
Tibet	17	11	0.95 ± 0.03	0.026 ± 0.014	12.40	4.44 ± 1.88	5.93 ± 3.33	0.019
Iran (ancient)	12	9	0.94 ± 0.06	0.043 ± 0.024	14.69	9.27 ± 3.89	9.89 ± 5.49	0.040
Iran-Turkmenistan (modern)	45	7	0.76 ± 0.05	0.028 ± 0.015	2.08	5.49 ± 1.87	6.51 ± 3.48	0.024
Caucasus	5	2	0.60 ± 0.18	0.008 ± 0.006	0.69	1.44 ± 1.02	1.80 ± 1.44	0.006
Anatolia-Balkans with Artenac	9	5	0.83 ± 0.10	0.018 ± 0.011	3.83	4.42 ± 2.16	4.11 ± 2.56	0.019
Anatolia-Balkans without Artenac	8	4	0.79 ± 0.11	0.012 ± 0.008	2.50	2.31 ± 1.31	2.79 ± 1.87	0.010
Syria	6	3	0.60 ± 0.21	0.005 ± 0.004	1.70	1.31 ± 0.91	1.20 ± 1.02	0.006

Nind: number of individuals, Nhap: number of haplotypes. The ± indicates the standard deviation. These summary statistics were calculated using Arlequin and DNAsp.

## Results

In order to characterize the ancient and extant genetic diversity and population structure, we studied the mitochondrial lineages of the wild asses from Europe and Asia, over the last 100,000 years from 70 sites in Europe and Asia. We targeted a 295-bp-region in the *E*. *hemionus* mitogenome that encompasses a specific 28-bp-deletion, absent in other equids, which is a useful barcode for this taxonomic group of *Equus*. Although this choice restricted our analyses to a single marker of the maternal lineage, thus limiting the phylogenetic resolution and information that can be obtained [17], making use of this high copy marker allowed us to include a large number of important ancient samples from warm environments, which would otherwise have been excluded having extremely poor DNA preservation. We obtained DNA sequences from (i) 57 out of the 189 archeological samples analyzed that had been attributed on morphological grounds to *E*. *hemionus*, *E*. *kiang*, or *E*. *hydruntinus*: (ii) all of the 11 historical museum specimens analyzed (between 60 and 180 years old) of onagers, hemippi, khurs and kiangs; (iii) 53 present-day samples, 94% of which originated from wild individuals, coming from the Gobi Desert (dziggetais) and from protected nature reserves in Iran and Israel. These sequences were enriched by 32 sequences of present-day individuals and one sequence of an ancient specimen published in GenBank. Since several of the archeological samples yielded only partial coverage of the full 295-bp-region (see Fig A in S2 File), we searched for the optimal combination balancing sequence length with sample representation. To satisfy these criteria, we selected a 229-bp sub-region for phylogenetic analyses that allowed for the inclusion of 27 archeological samples, 9 museum specimens, and all modern samples.

### Population structure of the wild ass in Asia and Europe

To investigate the potential relationship between geographic distances and genetic variation we used a spatial principal component analysis (sPCA; [32]) that integrates genetic data and georeferenced positions of the samples and displays the three principal component relationships as a color code, allowing a visual characterization of the sPCA results (Fig 1B; see also Material & methods and Table D in S1 File). These sPCA-derived color codes were further used to represent the samples in the other genetic and phylogenetic analyses presented in this study, such as the representation of the relationships between sequences with the median joining network (MJN, Fig 2A), as well as maximum likelihood (ML, Figs 2B and 4B) and Bayesian (Fig 3; Fig H in S2 File) phylogenies.

The sPCA reveals a clear phylogeographic structure of the data. The MJN and phylogenetic analyses show the sequences recovered belonging to eleven clades that we named with letters coding for either their geographical origin or their taxon (Figs 2B and 3). Each of the eleven clades is essentially dominant in a distinct geographical territory, i.e., Anatolia-Balkans-western Europe, Syria, the Caucasus, the Tibetan Plateau, modern Iran-Turkmenistan and northwest India, apart from ancient Iran and the Gobi, which contain three different clades each. In the following, we will consider only the most robust phylogenetic relationships between clades that were consistently observed irrespective of the phylogenetic analysis method used (MJN, ML, and Bayesian).

We used summary statistic approaches to analyze the genetic diversity within and between each territory (Tables 1–3). The analysis of the genetic distance between the populations as expressed through the fixation index F_ST_ is reported in Table 1. While there is moderate genetic differentiation between the modern populations from the Gobi and the Tibetan plateau (F_ST_ = 0.104), and between the ancient Caucasian and Iranian populations (F_ST_ = 0.1), the other populations including the modern wild asses from Iran and Turkmenistan, as well as the ancient populations from Syria, Anatolia and the Balkans are highly differentiated with F_ST_ values between 0.33 and 0.82, a differentiation with high statistical support (Table 1).

Several measures of molecular genetic variability within populations were used, namely nucleotide diversity Pi (Π) and the population parameter Theta (ϴ) as estimated using several methods (Table 3). The highest intra-population genetic diversity was detected in the ancient Iranian and extant Gobi populations followed by the extant population of the Tibetan plateau (Table 3). The other populations are less diverse, the least diverse being the ancient, extinct populations from Anatolia and the Balkans, the Caucasus, and Syria (Table 3).

In the following we describe the various clades grouped by larger geographical regions and correlate them with present-day subspecies.

***I*, *CI* and *TI* clades: onagers and kulans.** The ancient (9,000 to 3,000-year-old) and modern Iranian specimens were found to belong to three clades (Figs 2 and 3) named *I*, *CI* and *TI* that are distributed over a large portion of the phylogenetic trees (Figs 2 and 3). The *CI* clade includes the ancient samples from the Caucasus and Iran and shows a high diversity. All but one of the ancient Caucasian samples belong to this clade (Fig 1B). The Caucasian population has since disappeared and the *CI* clade is presently poorly represented in Iran with only a single present-day sequence in the database [44].

A few ancient (9,000 to 3,000-year-old) and most present**-**day Iranian onagers (*E*. *h*. *onager*) belong to the *I* clade whereas the present-day Turkmen kulans (*E*. *h*. *kulan*) belong to the *TI* clade (Figs 2 and 3). A small number of both ancient (8,000 to 3,000-year-old) and modern Iranian onagers also belong to the *TI* clade, albeit to a sub-clade (TI*) that diverged from Turkmen kulans at an early stage of the radiation of the *TI* clade. Consistent with the above findings, the wild ass colony that was established in Israel in the 1970s from 6 Iranian and 5 Turkmen wild asses belong to both the *I* and *TI* clades.

***Kh* clade: khurs.** The wild asses of northern India, or khurs (*E*. *h*. *khur*), are at present found in the reserve of the Rann of Kachchh (Kutch) and its surroundings. We analyzed 19^th^ century museum specimens of the khur originating from northwest India. Their sequences form the *Kh* clade (Figs 2 and 3). HVR sequences (240 bp) from the present-day khurs of Rann of Kachchh [48] share with the historic khur samples the characteristic SNPs of the *Kh* clade (Fig B in S2 File).

***K*, *KD*, *D1* and *D2* clades: kiangs and dziggetais.** Mongolia and Tibet host two populations of wild asses: dziggetais (also called “kulans”; *E*. *h*. *hemionus*), and kiangs (*E*. *kiang*). Our findings show the intrapopulation nucleotide diversity of the Mongolian dziggetais to be the highest of all present-day wild ass populations, similar only to that found in the ancient Iranian population (Table 3). This high diversity suggests that these populations have not been subject to the severe bottlenecks that appear to have affected other modern hemione populations. All dziggetais studied correspond to present-day individuals and belong to either the *KD*, *D1 or D2* clades (dziggetais are indicated by a red dot in Figs 2–4). The 17 analyzed kiangs, corresponding to museum specimens from the 19^th^ century as well as present-day individuals, belong to the *KD* and *K* clades. The branches of the *K*, *KD*, *D1 and D2* clades emerge at different locations in the phylogenetic trees (Figs 2 and 3). The *K* and *KD* clades are phylogenetically closely related (Figs 2 and 3). The *K* clade encompasses only kiangs, in particular the wild kiangs from the southern part of Tibet (Fig 4). The present-day dziggetais from the *KD* clade have sequences that are closely related to those of kiangs found outside of southern Tibet, which include 60% of the kiangs originating from zoos as well as those from an area north of Tibet [49] (Fig 4).

**Clade *S*: hemippi.** The smallest of the Eurasiatic wild asses, the Syrian wild ass, or hemippus (*E*. *h*. *hemippus*), which stood only one meter at the withers, is now extinct. The last recorded animal was captured in the desert of Alep and died in 1929 in Vienna (Fig P in S2 File) [50]. Expectedly, the mitochondrial lineages from four museum samples of the 19^th^ and 20^th^ century cluster together but, strikingly, these same lineages are also found in ancient specimens from Tall Munbāqa (Syria), located on the Middle Syrian Euphrates and dating to ca. 1,500–1,200 BCE. Together, they form the *S* clade that is related to the *CI* and *D1* clades (Figs 2 and 3). The paternal lineage of the hemippus based on the analysis of the Y chromosomes of both ancient and museum samples is also distinct from that found in other wild asses (Bennett et al., in preparation), indicating a continued reproductive isolation of this group.

Our observation of the genetic continuity over 5,000 years of the Syrian wild ass population led us to revisit the observation that Syrian wild asses from the site of Shams ed-Din Tannira (6^th^ millennium BCE) were larger than the modern hemippi [51]. We thus compared osteometric data from the Shams ed-Din Tannira specimens with those of this study: the 19^th^-20^th^ century hemippi, and Bronze Age Syrian wild asses from Tell Munbāqa (2^nd^ millennium BCE). For this comparison osteometric data (provided by L. Gourichon and D. Helmer) from 10^th^-9^th^ millennia BCE Tell Mureybet, located in the middle Syrian Euphrates valley, were also used, and, as an outgroup, individuals from Göbekli Tepe (10^th^-9^th^ millennia BCE), about 150 km further north in southeastern Anatolia, where genetically determined individuals belonged to clade *CI*. The comparison of various measurements from post-cranial skeletal parts reveals that the prehistoric Syrian individuals range within the upper part of the size variation of their modern descendants or even surpass these in size (Supporting Information, section III.1.4. in S2 File; Table H in S1 File and Fig K-L in S2 File). This analysis showed a difference in average bone size between the prehistoric Syrian and Anatolian sub-populations: the wild asses hunted near Göbekli Tepe were clearly heavier than those living further south (Tell Mureybet and Munbāqa). The difference from these two areas both in bone size and of the two corresponding mitochondrial clades point to two distinct morphotypes. Thus, even though the prehistoric Syrian wild asses were already smaller than their neighboring Anatolian relatives, they were still of a more robust build compared to their modern descendants.

***H1* and *H2* clades: hydruntines.** After observing inconsistencies between our preliminary genetic data and the initial taxonomic assignment of certain remains, we subjected all available bone and tooth samples from which we obtained DNA sequences to a “cross-determination” performed as a blind test by several of the osteologists participating in this research. In a number of cases, substantial disagreement among them was observed (Table K in S1 File). As emphasized previously ([9] and citations therein), this demonstrates the difficulties of assigning equid remains to (sub)species level based on osteomorphology and/or osteometry. For this reason, paleontologists tend to include non-morphological criteria in their taxonomic assessments, such as the time period and the geographical area from which the specimen in question originated, a strategy which can lead to circular reasoning (see discussion in [9]). Accurate taxonomic identification of hydruntine (*E*. *hydruntinus*), known only from fossil remains for which osteological and odontological diversity have not been well characterized, proved to be particularly problematic. In our study, 40% of the bones and teeth assigned on osteomorphological grounds to hydruntine by a subset of the osteologists involved yielded sequences clustering with *E*. *caballus*. Some of the caballine sequences obtained from these ancient specimens are either at present extinct or have not yet been found in modern horses (data not shown). Thus, for this taxon we adopted a ‘specificity index’ based on the degree of agreement among the specialists of equine osteomorphology that allowed us to consider archeological specimens most confidently assigned as hydruntine (see discussion in the Supporting Information section III.3 in S2 File, Fig O in S2 File and Table K in S1 File). This procedure enabled us to characterize the distinct genetic structure of these ancient populations that has eluded previous attempts.

This approach revealed an extinct clade, *H1*, comprising sequences from archaeological specimens dated from 5,000 to 8,000 years BP, from bones and teeth that achieved the highest specificity index in our classification of morphological determination of hydruntine (Table K in S1 File; Fig O in S2 File). This group comprises all remains that were identified as hydruntine with a specificity index >0.5 including the three remains all osteologists unanimously agreed upon. Nearly all specimens found in Anatolia and the Balkans belong to the *H1* clade (Figs 2 and 3). Despite their geographical proximity, the *H1* clade is genetically distant from the *S*, *CI* and *I* clades and appears more closely related to the *TI and Kh* clades suggesting a different phylogenetic history than that of its geographic neighbors. Additionally, a metapodial from the Early Upper Pleistocene cave of Artenac, France, with a stratigraphic age of roughly 100,000 years was determined as clearly belonging to *E*. *hydruntinus* with a specificity index of 1 (Table K in S1 File; Fig O in S2 File). This sample yielded a mitochondrial sequence that was assigned to a distinct *H1*-related clade, *H2*. Surprisingly, this clade was also found in an early 20^th^ century museum specimen originating from Iran. This allowed us to identify two mitotypes: *H1* and *H2*, most likely representing the extinct hydruntine. All presumed hydruntines that belonged genetically to another wild ass clade had an average specificity index at least twice as low (Fig O in S2 File). The phylogenetic proximity of the *H1* and *H2* clades with the *TI* and *Kh* clades (Figs 2B and 3) indicates the hydruntine to be a subspecies of *E*. *hemionus*, to the same degree as kiangs, dziggetais, hemippi, kulans, khurs, extinct and extant onagers, altogether forming a group that can be referred to as the Eurasiatic wild ass.

## Discussion

### Origins

Equids evolved in North America before dispersing into Asia around 2.6 million years ago (Mya) [52]. Genomic data indicate that caballoid and stenonid equids diverged from each other ca. 4.5 Mya, and that gene flow between them ceased around the beginning of the Asian dispersal [14]. Among stenonids, the separation of asses and zebras has been estimated at ca. 2 Mya while that of African and Eurasiatic asses has been estimated at ca. 1.7 Mya ([14], see Fig I in S2 File). Our data, based on the mitochondrial HVR, are consistent with these estimates (Fig 3, Figs H-I in S2 File). Since the mitochondrial genomes of all Eurasiatic wild asses are characterized by a 28-bp deletion that has a very low probability of being homoplasic, they most likely constitute a monophyletic group. Where did this group emerge? All present-day stenonids are African, except for the Eurasiatic wild asses, and the earliest datable fossil evidence of *Equus* in Africa occurs ca. 2.33 Mya [53]. Thus, it could be hypothesized that the last common ancestor of all present-day stenonids was African. In this scenario, the Eurasiatic wild ass could have emerged ca 1.7 Mya from an ancestral ass-like population living in northern Africa, the Arabian peninsula and the Levant. Since all Eurasiatic wild ass mitogenomes evolved from an ancestral stenonid mitogenome after a single 28-bp-deletion event has occurred, the ancestral population of the Eurasiatic wild ass must have gone through a severe bottleneck before or during its migration into Eurasia. Alternatively, the ancestral stenonids population could have evolved in the plains of central Asia, from where at least two independent migration waves into Africa gave rise first to the zebras around 2 Mya, and second to the African asses ca. 1.7 Mya. In this scenario, a severe bottleneck must have affected the population that remained in Asia leaving only the descendents of the deletion-harboring lineage that emerged between about 700 and 800 kiloyears ago ([14, 44] and Fig 3). The mitogenome sequence of a ca. 45,000-year-old Siberian *Sussemionus (E*. *ovodovi)* is in favor of this latter hypothesis since it reveals an extinct Asian lineage that has diverged from other stenonids around the time of separation of the zebras and the asses ([44]; Fig I in S2 File). Genomic data do not yet enable us to decide which of the two hypotheses is more likely, and fossil evidence in certain regions is lacking, due in part to taphonomic reasons, but also to the rarity of detailed morphological description, consistent analyses and rigorous comparison [53]. Following divergence from other stenonids, the ancestral population of hemiones would have dispersed on the Eurasiatic continent where the populations would have further evolved and phylogeographic stratification taken place.

The phylogenetic relationships between the various mitogenome clades do not reveal a simple relationship with geographical distance but rather suggest a complex phylogeographic history with back-and-forth migrations. It is important to note that we base our conclusions only on those differences between clades that are found irrespective of the phylogenetic methods used and that have the strongest support. Our data suggest that Eurasiatic wild asses harboring the *KD/K* clade mitogenomes may have migrated during the Middle Pleistocene into the Gobi and Tibet where they evolved independently. Eurasiatic wild asses with either of the *I*, *CI*, *S*, *D1* clade mitogenomes may have evolved in Southwest Asia where most of them were found in the Holocene, from where some of them (*D1* clade) migrated to the Gobi, presumably not before the end of the Middle Pleistocene. Central Asia, where clade *TI* is still found, may also have allowed evolution of the Eurasiatic wild asses that are related to the *TI* clade and that have spread into Europe (*H1* and *H2* clades), India (*Kh* clade) and the Gobi (*D2* clade). Since the *H1* and *H2* clades are more distantly related to the clades established in Southwest Asia (*I*, *CI*, *S*) than to the *TI* clade (Figs 2 and 3, see also Tables 1 and 2), we hypothesize that they have colonized Europe during the Pleistocene through a route skirting Southwest Asia, for example through a northern route involving the Pontic-Caspian Steppes, and that they arrived later in Anatolia coming from Europe at times when the Bosporus was a land bridge. Such a scenario would explain the strong differentiation of the *H1* clade in Anatolia with respect to the geographically neighboring populations of the Syrian hemippi *S* and the Iranian onagers *I* and *CI* (Tables 1 and 2) as well as their closer relatedness to the Turkmen kulans *TI*, the Indian khurs *Kh* and the Mongolian dziggetais *D2* (Figs 2 and 3).

The colonization of Northeast Asia was likely to have involved several waves. Since the *K/KD* clades are the most distantly related to the other clades (Figs 2 and 3) and the Bayesian analysis (Fig 3) indicates that they were the earliest to diverge from the other hemiones, they may descend from the initial population that established itself in northeast Asia and adapted to the high altitude of Tibet. The sampling of the present-day wild populations is biased in favor of the southern Tibetan population from the natural reserves of Nyalam, Gyirong, Ngamring and Tingri ([54] and Fig 4A). The corresponding samples belong to the *K* clade alongside a 19^th^ century museum sample and two zoo specimens (Fig 4B). All other kiang sequences belong to the *KD* clade and correspond to zoo and museum specimens of various origins as well as to samples from wild animals collected north of Tibet (Fig 4B; Tab. C in S1 File). This suggests that the *KD* and *K* clades correspond to the northern and southern Tibetan kiang populations, respectively, and the two corresponding populations may have had limited exchange due to the Himalayan chain.

In contrast, three phylogenetically distant mitochondrial clades, *KD*, *D1* and *D2*, are found in the dziggetai populations from Mongolia, which suggests that they may have resulted from three colonization waves: the first corresponded to the initial population giving rise to the kiang that could have extended from Mongolia to northern Tibet (*KD*). The later colonization waves introduced the *D2* clade that is most closely related to the Turkmen *TI* clade, as well as the *D1* clade that is most closely related to the Caucasian (*CI*) and Syrian (*S*) clades. The different phylogenetic affiliations of these latter two clades suggest independent colonization events. Since a large part of the diversity of the mitogenomes of the *KD* clade is found among both kiangs and dziggetais, including recently evolved haplotypes (compare the distribution of the dziggetais indicated by a red dot with that of the kiangs on the phylogenetic trees shown in Figs 2–4) and that very similar sequences are found in the two populations, there must have been multiple admixture events in the more recent past. These must have been asymmetric, because 10 dziggetais belong to the *D(1+2)* and 13 to the *KD* clade, but none of the 17 kiangs belonged to either the *D1 or D2* clade. A Fisher exact test indicates that there is a probability of only 0.2% that such an unequal distribution would be observed in the absence of asymmetric gene flow. Different scenarios could account for this asymmetry. In the first scenario, asses of the *D1* and *D2* clades would have arrived in Mongolia already occupied by kiangs, whose range extended from Mongolia to Tibet. The various ass populations would have interbred in Mongolia giving rise to the present-day dziggetais. The later arriving asses of the *D1* and *D2* clades would not have pursued their migration to Tibet, maybe because they were not adapted to high altitude. An alternative scenario would be that the members of the northern Tibetan kiang population migrated regularly from the Himalayas to the Mongolian plain and interbred with the Mongolian asses. Whatever the scenario, the interbred Mongolian population does not appear to have migrated back to the highlands of Tibet. The regular introgression of mitochondrial genomes from northern Tibetan kiangs to dziggetais must have occurred rather recently, in the Late Pleistocene or early Holocene at the latest, given the similarity between the shared HVR sequences. These recent and multiple admixtures question the validity of the classification of the kiang as a separate species (e.g., [55]).

### Taxonomy and conservation biology

Conservation programs aim to preserve the evolutionary potential of a species using the classification of populations by their evolutionary significance based on ecological, morphological, geographic and genetic criteria [56,57]. The characterization of clades presented in this study thus provides a helpful guide for taxonomy and conservation biology. Our dataset reveals events of past and recent mitochondrial introgression between populations that are now considered separate species, such as kiang (*E*. *kiang*), or subspecies, such as onager (*E*. *h*. *onager*) and kulan (*E*. *h*. *kulan*) [55]. Poor genetic differentiation between kiangs and dziggetais (*E*. *h*. *hemionus*) has also been observed in a microsatellite study of equid diversity involving a smaller sample size (6 kiangs and 3 dziggetais) [58], indicating that our observation is not a peculiarity of the mitogenome transmission. We believe it may be more appropriate to consider the kiang as a distinct population or perhaps even a metapopulation [59] of *E*. *hemionus*, with specific adaptations to the high-altitude climate and vegetation of the Tibetan plateau.

The designation of onagers and kulans as separate evolutionary significant units has been questioned [60]. Among the three clades that had representatives in Iran during the last 8,000 years, the *I* clade remained centered in Iran and is the prevalent clade in present-day onagers; the *CI* clade shows a cline towards the Caucasus, where the corresponding population is now extinct, and the *TI* clade shows a cline towards Turkmenistan. All three clades coexisted in the past at a single location near present-day Tehran (Sagzabad, 3,500 years ago), and members of these clades are still interbreeding, showing that these clades do not define true diverging allopatric lineages. Currently, Iranian and Turkmen wild asses kept in the Hai-Bar Yotveta reserve in Israel are reported to interbreed and hybrids thrive without showing signs of outbreeding depression [23,61]. Given the fact that the endemic relict populations in Iran and Turkmenistan are shrinking rapidly, it is worth considering that in a not so distant past, when they occupied large interconnected areas, crosses between neighboring populations allowed gene flow events that have only recently been interrupted, enhancing the risk of inbreeding depression. Ensuring the survival of the Asiatic wild ass is a challenge that may justify managing the last remaining populations as components of a viable metapopulation [62].

### Palaeoecology of the Eurasiatic wild ass

The repeated glaciations alternating with warmer phases throughout the Pleistocene had major impacts on the fauna, flora and the environment (e.g., [63,64]). These climatic oscillations were likely to also affect the distribution, speciation and population size of the wild asses. In Western Europe, hydruntines were present only during the warmer and more humid interglacial periods of the Pleistocene (e.g., [11,65]). This Western European ecomorphotype was apparently adapted to milder climatic conditions and hilly landscapes (e.g., [11,65]). Indeed, the analyzed specimens from the caves of Artenac and Quéroy in western France were dated to ca. 100,000 (the Eemian interglacial) and ca. 12,700 years ago, respectively, periods characterized by a milder climate corresponding to Marine Oxygen Isotope Stages 5 and 1 (e.g., [66–68]). The populations of *E*. *hydruntinus* adapted to the warmer and more humid climate in Europe during the interglacial stages were probably repeatedly separated from each other and/or went locally extinct during subsequent glacial periods [6], a process that was possibly accelerated through competition with cold-adapted horses [69]. For example, the historical onager we identified as belonging to the same *H2* clade as the ca. 100,000-year-old individual from the Artenac cave in France might be a descendant of the populations that retracted to the northern Middle East during the Lower Pleniglacial cold period roughly 70,000 years ago (Fig 3). Correspondingly, during the cold periods of the Pleistocene, the European wild ass likely withdrew to Southwest Asia, solely or in addition to the southern European glacial refuges, a behavior we also observed for the European bison [64].

The genetic structure of the Asiatic population of the wild ass seems conditioned by geographical and climatic factors: the Asiatic steppe belt, the Iranian highlands and the Kara Kum desert in Turkmenistan, the mountainous Armenian highlands (Caucasus and western Iran), the arid lowlands of Syria-Mesopotamia, the Anatolian highlands and the Balkans. Each of these ecogeographical units harbored a genetically distinct population, which therefore can be considered to be different ecomorphotypes. During the last glacial maximum, Anatolia’s forests and woodlands disappeared and were replaced by cold steppe vegetation [70], climatic conditions that could be compared to those of present-day Tibet where kiangs live today. Thus, it may have been a favorable habitat for the Asiatic wild ass. Colder periods in the Pleistocene and the beginning of the Holocene, when the sea level was low, would have allowed exchange between the European and the Anatolian populations until around 10,600–7,600 BP, the presumed date of the filling of the Black sea (e.g., [71]). The steppe vegetation remained dominant in Anatolia even after 10,000 BP, when humidity increased, slowly being replaced by woodlands, a change that reached its peak around 8,000 BP, followed by a last regression until 6,800 BP [70]. Unstable climatic conditions which began after 8,200 BP were marked by climatic oscillations and fluctuating hydromorphological conditions, leading to drought and heavy floods and to the increase of swamps in central Anatolia [72]. This, alongside anthropogenic pressure, could have been one of the reasons for the gradual disappearance of the Anatolian-Balkans population. The most recent specimens from the Anatolian plain harboring the *H1* mitotype date from 4,200–4,000 BP setting a lower limit for the date of disappearance of the hydruntine in Anatolia.

The differentiation of the Syrian population with respect to the Iranian and Anatolian populations could be explained by a long independent evolutionary history of these wild ass populations caused by the ecological barriers of the Zagros and the Taurus mountain ranges, which separate the Syrian plain from the plateaus in Anatolia and Iran. South of the Taurus Mountains, landscape and vegetation near the headwaters of the Upper Balikh received more precipitation than the Middle Syrian Euphrates Valley near Tell Mureybet and likely offered more favorable living conditions to *E*. *hemionus* [73]. This may explain the larger size of the Early Neolithic *E*. *hemionus* from southeastern Anatolian Göbekli Tepe compared to the contemporaneous specimens from Middle Syrian Tell Mureybet and Late Bronze Age Tell Munbāqa. Following the main vegetation zones [73], the larger subspecies was associated with the xerophilous deciduous forest steppe, while the smaller one was confined to the Mesopotamian steppes. Drought, desertification, overgrazing by livestock and increased anthropogenization of the landscape combined with hunting pressure may explain the decline and extinction of the hemippus. Despite genetic continuity through time, the Syrian wild ass population witnessed a significant and previously unreported reduction in body size preceding its extinction. The new genetic evidence presented here supports the hypothesis that the animal became smaller in the relatively recent stages of its evolution preceding its extinction [51]. This might be the result of impoverished living conditions and/or increasingly arid conditions in historical times [74] pushing it into an ecological pessimum or the consequence of inbreeding depression due to a decrease in population size (see Supporting Information section III 1.4.1 in S2 File).

The Asiatic populations of the northeast (kiangs and dziggetais) and of the southeast (onagers, Turkmen kulans, khurs and hemippi) have been continually separated by the Central Asian massif, which was covered by glaciers during the cold periods [75]. Nevertheless, at least limited gene flow between populations of Eurasiatic wild asses could have taken place via the Irtych valley and the Dzungarian basin, which were accessible owing to a very dry climate [6]. The observation that the extant dziggetai *D1* and *D2* clades cluster with the ancient and present-day onagers and kulans argues in favor of the existence in the rather recent past of a population continuum with introgression from Iran and/or Central Asia to the Gobi.

In summary, in the past, the Eurasiatic wild ass was able to adapt to changing climatic conditions through population range shifts, which has become increasingly difficult due to habitat destruction and fragmentation, preventing the animals from reacting according to their natural behavior and local habitat conditions [76].

### *H1* and *H2*, the hydruntine mitotype

It was formerly assumed, based on geographic distribution through time, rate of speciation and capacity for sympatry deduced from morphological features of fossil remains, that stenonid and caballoid horses of the genus *Equus* differed ecologically, the stenonids being more specialized and therefore adapted to narrower niches [77]. In contrast, the ecological flexibility in the caballoids was considered to be the consequence of behavioral versatility rather than increased morphological variation [77]. The present results question this conclusion since fossils with presumed stenonid features were found during the course of this study to show a caballine mitotype. Further genetic analysis of three of these samples revealed SNPs of a Y-chromosome marker specific for *E*. *caballus* (Table A in S1 File), thus ruling out the possibility that these animals were F1 hybrids. This result rather indicates that the morphological plasticity of past equids appears to be higher than previously assumed and that some criteria used to determine species within this genus are in fact pleiomorphic.

**Fig 5:**
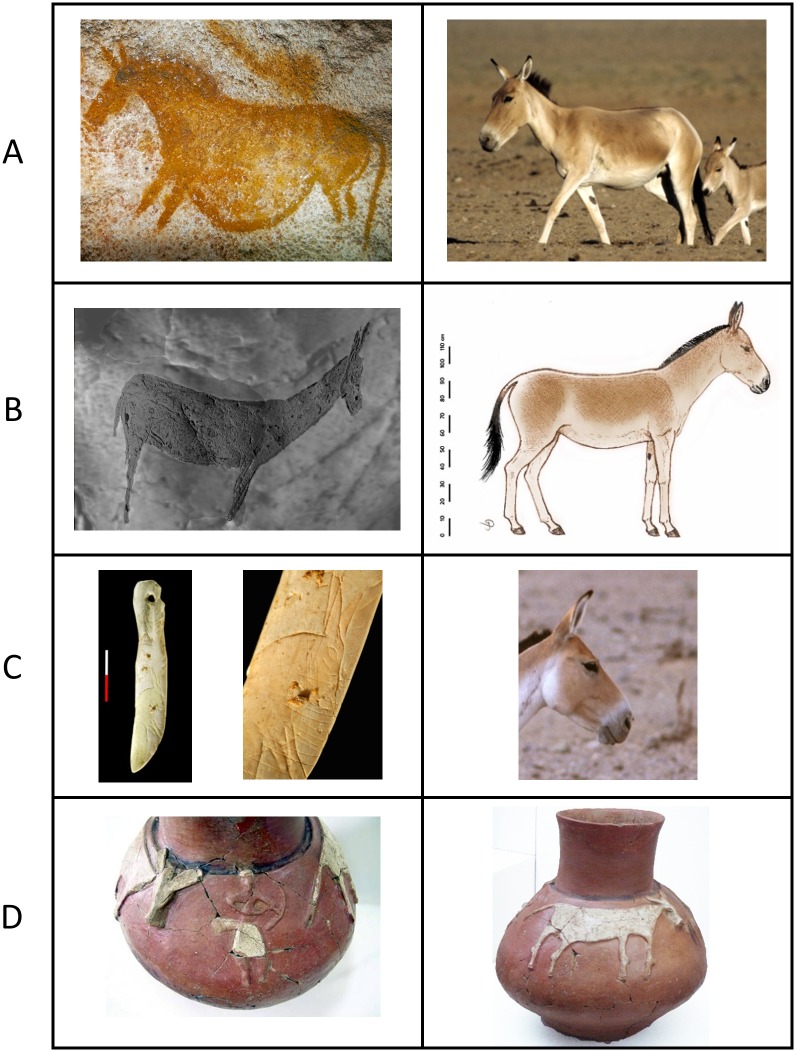
Ancient representations of the Eurasiatic wild asses. **A:** Comparison of the “Panel of the hemione” from the Lascaux cave, France (photo & copyright*: J.-M. Geneste, Centre National de Préhistoire-MCC) with a Dziggetai from the southeastern Gobi in Mongolia (photo & copyright*: P. Kaczensky). **B:** Engraving of a presumed hydruntine in the cave “Les Trois Frères”, Grottes des Volpes, France (photo modified from an original of Robert Begouën by selective contrasting to enhance the visibility of the carving; original photo & copyright*: R. Begouën), and graphical reconstruction of a hydruntine based on prehistoric engravings as well as the morphological and genetic results presented here (drawing and copyright*: Erich Pucher). **C**: Comparison of an engraving of a presumed hydruntine on a pendant in the cave of Putois, France (photo & copyright*: Ph. Jugie, Musée National de Préhistoire MNP, Les Eyzies, Dist.RMN) with an onager from Touran, Iran (photo & copyright*: Gertrud & Helmut Denzau). **D**: Depiction of a hunt scene of presumable hydruntines on a vessel from the Late Neolithic site of Köşk Höyük in Anatolia (photo & copyright*: Aliye Öztan). *Reprinted under a CC BY license, with permission from the copyright holders mentioned above.

Perhaps due to incorrect taxonomic identification of samples, no hydruntine-specific mitochondrial signature had been found in previous paleogenetic studies, which considered the hydruntine to be an onager-like wild ass [16,17]. Despite these identification difficulties, we could obtain data from a sufficient number of consensually assigned hydruntine bones to establish that the *E*. *hemionus H1* and *H2* clades correspond to the paleontological species of *E*. *hydruntinus* (see section III.3 in S2 File and table K in S1 File). The co-occurrence of an *E*. *hemionus* mitotype signature with a set of distinctive morphological features found in this study argues in favor of the hydruntine being a particular morphotype or ecomorphotype of *E*. *hemionus*. Since the separation of the *H1+2* clades from other mitotypes of *E*. *hemionus* is not as ancient as that separating mitotypes of interfertile populations, like the *KD*, *D1* and *D2* clades of the dziggetais, our data do not support the classification of the hydruntine as a distinct species. Instead, it is probably more appropriate to consider it a subspecies (*Equus hemionus hydruntinus*), as has been proposed for other current Eurasiatic wild ass populations, even though it is not clear whether in biological terms this level of taxonomic differentiation corresponds to something more than naming a population. The identification of the hydruntine as a Eurasiatic wild ass finds additional support in contemporary cave art representations, such as in Lascaux cave (Fig 5). The presence of *E*. *hemionus* in Europe when these works were created challenges the assumption that, due to a previously presumed absence of this species in Europe during the Upper Paleolithic, these images must therefore represent “deformed” horses [78,79]. Representations of the hydruntine in other French caves (Engraving in the cave “Les Trois Frères”, Grottes des Volpes, France; engraving on a pendant in the cave of Putois, France) resemble present-day hemiones (Fig 5B and 5C) but show even longer ears. Strikingly, long ears are also a distinct feature of the wild asses represented in hunt scenes on Late Neolithic vessels excavated from the Anatolian site of Kösk Höyük (Fig 5D). In this site we found eight equid bones with H1 haplotype suggesting that these depictions are representations of the local hydruntine since there is no evidence for donkeys in Anatolia until the 4th millennium BC at the earliest (e.g., [80,81]). Altogether these representations suggest that the animals’ ears were characteristic enough to be depicted. Their similarity lends further support to our finding that the hydruntine populations from Anatolia and Europe were closely related.

The surprising recovery of the *H2* subgroup known from a 100,000-year-old specimen from Western Europe, which may represent an older hydruntine population, from an early 20^th^ century museum specimen from Persia, suggests the possibility of ancient interbreeding events between hydruntines and other Eurasiatic wild asses. Two other specimens yielded incomplete sequences that were nevertheless sufficient to assign them to the *H1* subgroup, one from the Quéroy cave in western France, 12–13,000 years BP, and one from Scladina cave [16] in Belgium, estimated to be between 30,000 and 40,000 years old (D. Bonjean, pers. comm.). Later specimens from Romania and Turkey dated at 8,000 to 4,000 years BP belong to the *H1* subgroup. Thus, all specimens belonging to the H1 subgroup, which shows a low level of diversity, are between 40,000 to 4,000 years old (see Table A in S1 File). The distance from the *H1* to the *H2* mitotype suggests that the hydruntine population could have gone through a bottleneck during the last two glacial periods and that Europe could have been recolonized from a refugial population, as proposed earlier on morphological grounds [11]. Notably, “hydruntine” specimens post-dating 4,000 years BP do not contain the genetic signature of hydruntines (Table A in S1 File), but rather can be ascribed to other hemione clades. Our results are indicative of the disappearance of the hydruntine-type of Eurasiatic wild ass around the end of the Bronze Age, presumably following habitat fragmentation and human exploitation as proposed recently [12].

This characterization of a past population of Eurasiatic wild ass in Europe should be noted when considering low-intervention conservation management strategies of abandoned rural areas such as “rewilding”, where an abundant wild large herbivore population was concluded to have been instrumental in maintaining biodiversity of vegetation structures under a temperate climate in the absence of human management [82]. Although the challenges of the reintroduction of species which have disappeared from Europe a few millennia ago are many [83], consideration of the Eurasiatic wild ass may be appropriate for such initiatives.

## Supporting information

S1 File All tables are presented as separate spreadsheets consolidated into a single Excel file. Table A Description of all samples analyzed
Table B Primers used to amplify mitochondrial sequences
Table C Description of published sequences used
Table D Sample location and results of sPCA analysis
Table E Summary statistics for the kiangs and dziggetais of the K and KD clades using the 296 bp HVR sequence
Table F Characteristics, measurements (mm) and zoological assignment of samples from Iranian archeological sites
Table G Comparison of measurements from the sample DAG2 from Daghestan-Velikent with those from other hemiones and a kiang
Table H Comparative measurements of skeletal parts of late and Bronze Age *E*. *hemionus hemippus*
Table I Measurements of the teeth of the hydruntines of Cheia
Table J Comparative measurements (mm) of the analyzed metacarpals of the caves of Artenac and Pair-Non-Pair
Table K Determination of the specificity index of the hydruntine bones
Table L Studbook register of the founder animals from the Hai-Bar-Yotveta Reserve
(XLSX)

S2 File Supplementary figures of the phylogenetic analyses followed by the detailed description of the archeological sites and the samples analyzed grouped in a single supporting document. Figure A: Global alignment of all sequences obtained and used for the various analyses
Figure B: Diagnostic SNPs of the various clades
Figure C: sPCA, distribution of the eigenvalues
Figure D: sPCA, spatial and variance components of the eigenvalues
Figure E: sPCA, distribution of the first three principal components of the sPCA for samples colored according to their origin
Figure F: sPCA, histogram of the simulation to test the significance of the global structure
Figure G: sPCA, histogram of the simulation to test the significance of the local structure
Figure H: Phylogenetic tree of HVR constructed through BEAST analysis
Figure I: Dating estimates of the equine phylogeny
Figure J. Specimens from Norşun Tepe
Figure K: Osteometrical data from Syrian hemiones
Figure L: Logarithmic Size Index method
Figure M: Simpson diagram of the Artenac metacarpal
Figure N: Occlusal view of inferior jugal tooth from *E*. *ferus* “Grotte de Rochefort”
Figure O: Histogram of specificity index and clade of ‘*E*. *hydruntinus’* bone samples
Figure P: *E*. *h*. *hemippus*
Figure Q: Dziggetais at a pothole in the Gobi
Figure R: Onager and kiang
(PDF)
