# Supplementary material for: Taming the late Quaternary phylogeography of the Eurasiatic wild ass through ancient and modern DNA
Source: PLoS One. 2017 Apr 19;12(4):e0174216. doi: 10.1371/journal.pone.0174216 (PMC5396879; doi:10.1371/journal.pone.0174216)
Supplement: S2 File — Figure A: Global alignment of all sequences obtained and used for the various analyses Figure B: Diagnostic SNPs of the various clades Figure C: sPCA, distribution of the eigenvalues Figure D: sPCA, spatial and variance components of the eigenvalues Figure E: sPCA, distribution of the first three principal components of the sPCA for samples colored according to their origin Figure F: sPCA, histogram of the simulation to test the significance of the global structure Figure G: sPCA, histogram of the simulation to test the significance of the local structure Figure H: Phylogenetic tree of HVR constructed through BEAST analysis Figure I: Dating estimates of the equine phylogeny Figure J. Specimens from Norşun Tepe Figure K: Osteometrical data from Syrian hemiones Figure L: Logarithmic Size Index method Figure M: Simpson diagram of the Artenac metacarpal Figure N: Occlusal view of inferior jugal tooth from E. ferus “Grotte de Rochefort” Figure O: Histogram of specificity index and clade of ‘E. hydruntinus’ bone samples Figure P: E. h. hemippus Figure Q: Dziggetais at a pothole in the Gobi Figure R: Onager and kiang (PDF) [file pone.0174216.s002.pdf]

# Supporting information

## Taming the Late Quaternary phylogeography of the Eurasiatic wild ass through ancient and modern DNA

E. Andrew Bennett[1]*, Sophie Champlot[1]*, Joris Peters[2, 3], Benjamin S. Arbuckle[4], Silvia Guimaraes[1], Mélanie Pruvost[2†], Shirli Bar-David[5], Simon J.M. Davis[6], Mathieu Gautier[7], Petra Kaczensky[8], Ralph Kuehn[9], Marjan Mashkour[10], Arturo Morales-Muñiz[11], Erich Pucher[12], Jean-François Tournepiche[13], Hans-Peter Uerpmann[14], Adrian Bălăşescu[15], Mietje Germonpré[16], Can Y. Gündem[14‡], Mahmoud-Reza Hemami[17], Pierre-Elie Moullé[18], Aliye Ötzan[19], Margarete Uerpmann[14], Chris Walzer[8], Thierry Grange[1¤§], Eva-Maria Geigl[1¤§]

1, Institut Jacques Monod, UMR7592, CNRS-Université Paris Diderot, 15 rue Hélène Brion, 75013 Paris, France

2, Institute of Palaeoanatomy, Domestication Research and the History of Veterinary Medicine, Ludwig-Maximilian University, Munich, Germany

3, SNSB, Bavarian State Collection of Anthropology and Palaeoanatomy, Munich, Germany, Kaulbachstr. 37, 80539 München, Germany

4, Department of Anthropology, University of North Carolina at Chapel Hill, Chapel Hill, 104 Airport Dr. CB 1350, Suite 2200, Chapel Hill, N.C. 27599-135, USA

5, Mitrani Department of Desert Ecology, Jacob Blaustein Institutes for Desert Research, Ben-Gurion University of the Negev, Sede Boqer Campus, 84990 Midreshet Ben-Gurion, Israel

6, Laboratório de Arqueociências, (DGPC), Ajuda, Rua da Bica do Marquês 2,1300-087 Lisbon, Portugal

7, Centre de Biologie pour la Gestion des Populations CBGP, 755 avenue du campus Agropolis, CS30016, 34988 Montferrier-sur-Lez, France

8, Research Institute of Wildlife Ecology, University of Veterinary Medicine, Savoyenstrasse 1, A-1160 Vienna, Austria

9, Technische Universität München, AG Molekulare Zoologie/Lehrstuhl für Zoologie, Hans-Carl-von-Carlowitz-Platz 2, 85354 Freising, Germany

10, Muséum national d'Histoire naturelle, UMR 7209, Archéozoologie, archéobotanique: sociétés, pratiques et environnements, Département Ecologie et Gestion de la Biodiversité, 55, rue Buffon, 75005 Paris, France

11, Laboratory of Archaeozoology, Dept. Biologia, Universidad Autonoma de Madrid, C. Darwin 2, 28049 Madrid, Spain

12, Naturhistorisches Museum, Burgring 7, 1010 Vienna, Austria

13, Musée d'Angoulême, 1 rue Friedland, 16000 Angoulême, France

14, Eberhard-Karls-Universität Tübingen, Institut für Ur- und Frühgeschichte und Archäologie des Mittelalters, Abteilung Ältere Urgeschichte und Quartärökologie, Zentrum für Naturwissenschaftliche Archäologie, Rümelinstrasse 23, 7207 Tübingen, Germany

15, National History Museum of Romania, National Centre of Pluridisciplinary Research, Victoriei, no 12, Bucureşti 7041, Romania.

16, Royal Belgian Institute of Natural Sciences, Earth and History of Life, Vautierstraat 29, 1000 Brussels, Belgium

17, Department of Natural Resources, Isfahan University of Technology, Isfahan, Iran

18, Musée de Préhistoire Régionale, 8 Avenue Boyer Palais de l'Europe, 06500 Menton, France

19, Ankara Üniversitesi Dil ve Tarih-Coğrafya Fakültesi, Arkeoloji Bölümü 06100 Sıhhiye, Ankara, Turkey

*These authors contributed equally to the study

¤co-last authors

†Present address: Institut Jacques Monod CNRS-Université Paris Diderot, 15 rue Hélène Brion, 75013 Paris, France

‡Present address: Fen Edebiyat Fakültesi, Batman Üniversitesi, Batman, Turkey

§Corresponding authors: Eva-Maria Geigl and Thierry Grange, Institut Jacques Monod, UMR 7592, 15 rue Hélène Brion, 75013 Paris, France; eva-maria.geigl@ijm.fr; Thierry.grange@ijm.fr

# Table of Contents


Foreword ... 3

List of tables ... 3

List of figures ... 3

I. Phylogenetic analyses: Figures ... 4

II. Short description of the samples that yielded results ... 12

III. Archaeological samples ... 15

1. Southwest Asia ... 15
2. Europe ... 25
3. Specificity Index of samples initially determined as *E. hydruntinus* ... 31

IV. Historical specimens ... 33

1. E. hemippus ... 33
2. E. hemionus ... 33
3. E. kiang ... 34

V. Samples from present-day animals ... 35

1. Dziggetais ... 35
2. Onagers and kiang from the Sigean zoo ... 35
3. The Asiatic wild ass in Hai-Bar Yotveta breeding core, Israel ... 35
4. E. hemionus onager from the Bahram-e Goor reserve (Iran) ... 36
5. E. hemionus onager from the Khartouran reserve (Iran) ... 36

VI. References ... 37

## Foreword

This supplementary material document contains materials of interest to diverse scientific fields. To navigate smoothly within it, we recommend using the navigation pane to directly reach sections of interest to the reader. All 14 supplementary tables are presented in a single separate Excel file with 14 spreadsheets.

## List of tables

Table A Description of all samples analyzed
Table B Primers used to amplify mitochondrial sequences
Table C Description of published sequences used
Table D Sample location and results of sPCA analysis
Table E Summary statistics for the kiangs and dziggetais of the *K* and *KD* clades using the 296 bp HVR sequence
Table F Characteristics, measurements (mm) and zoological assignment of samples from Iranian archeological sites
Table G Comparison of measurements from the sample DAG2 from Daghestan-Velikent with those from other hemiones and a kiang
Table H Comparative measurements of skeletal parts of late and Bronze Age *E. hemionus hemippus*
Table I Measurements of the teeth of the hydruntines of Cheia
Table J Comparative measurements (mm) of the analyzed metacarpals of the caves of Artenac and Pair-Non-Pair
Table K Determination of the specificity index of the hydruntine bones
Table L Studbook register of the founder animals from the Hai-Bar-Yotveta Reserve

## List of figures

Figure A: Global alignment of all sequences obtained and used for the various analyses. 4
Figure B: Diagnostic SNPs of the various hemione clades. 6
Figure C: sPCA, distribution of the eigenvalues. 6
Figure D: sPCA, spatial and variance components of the eigenvalues (screeplot). 7
Figure E: sPCA, distribution of the first three principal components of the sPCA for samples colored according to their origin. 8
Figure F: sPCA, histogram of the simulation to test the significance of the global structure 8
Figure G: sPCA, histogram of the simulation to test the significance of the local structure 9
Figure H: Phylogenetic tree of Hemione mitochondrial control region constructed through BEAST analysis. 10
Figure I: Dating estimates of the equine phylogeny. 11
Figure J. Specimens from Norşun Tepe. 21
Figure K: Osteometrical data from Syrian hemiones. 24
Figure L: Logarithmic Size Index method for Syrian hemiones. 25
Figure M: Simpson diagram corresponding to the comparative measurements of the Artenac metacarpal. 29
Figure N: Occlusal view of inferior jugal tooth from the “Grotte de Rochefort”, attributed to *E. ferus* 30
Figure O: Histogram of specificity index and clade of bone samples considered to be *E. hydruntinus* 32
Figure P: *E. h. hemippus* 33
Figure Q: Dziggetais at a pothole in the Gobi. 35
Figure R: Onager and kiang 35

# I. Phylogenetic analyses: Figures

**Figure A: Global alignment of all sequences obtained and used for the various analyses.**

Differences from the consensus sequences are represented by colored vertical bars. Dark grey blocks represent missing sequences. Gaps are represented by thin horizontal lines. The locations of the primers used are represented alongside the *E. asinus* reference sequence that was used as an outgroup.

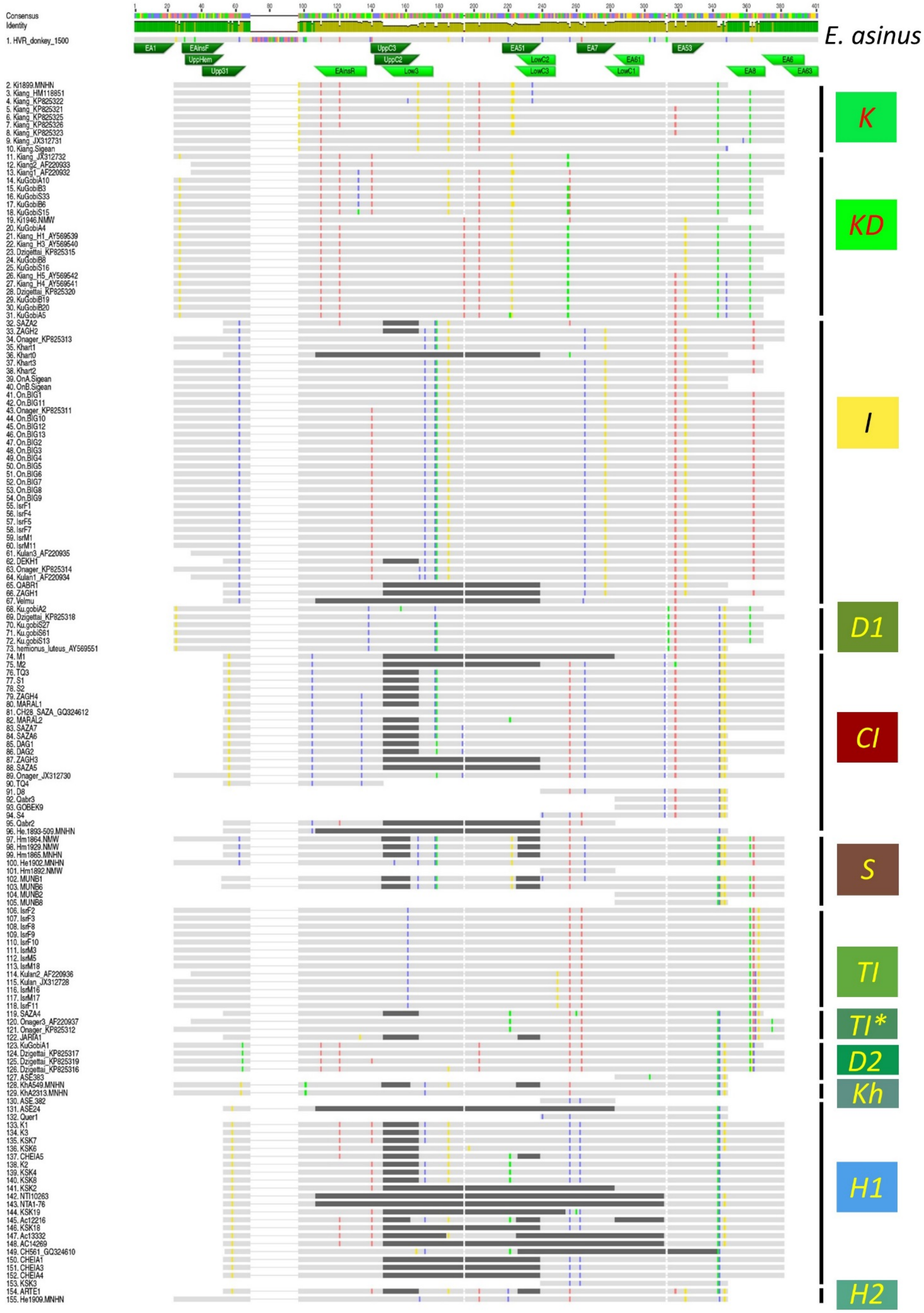
Consensus
Identity
1. HVR_donkey_1500
E. asinus
K
KD
I
D1
CI
S
TI
TI*
D2
Kh
H1
H2

**Figure B: The diagnostic SNPs of the various clades are represented below, using as a reference the full length mitochondrial genome of *Equus hemionus kulan* NC_018782.**

```
        111111111111111111111111111111111111111111111111111111111
        555555555555555555555555555555555555555555555555555555555
        445555555555555555555556666666666666666667777777777777778
        881111122334566688899900122244444678888990334466777888899 0
        021378948278915704845845201967890512890138939890233789020
                                  i
Ref     AAAATACACCGGATTGTCCATTTCAT-GTCAATGCATATTATAGACTAATTTAACGC
K       .......G..AR......TG....G..A..RRYA.G.G.....R.T.......GTA.
KD      .G........AAM..A..T.....R.aR..GR.AYR.G.....RRT...Y...GTA.
I       ....C..........R..T..CCTG........A.G.G.YG..AG......C..TA.
D1      G.............C..YT...CY.........A.G.G....TA..C.G....GTA?
CI      ..G......C...Y....T...YT.Y.......A...G.C.C.A..CGG..C.GTA.
S       ....Y...........C.TC..CT......G..A...G.C.....TCGG..Y..TA.
TI      .................................R....................Y..
TI*     ..................T..........T...A...........TC.........Y
D2      ......T...AA......T........A.....A...........TC.G...GCTA.
Kh      .....G..T.........T..C..R........A...G.......TC.G.???????
H1      ??.G.......R...R????.C.YG........A.CCG.......TC.R..C.GTA.
H2      ...R..............T.Y...R..AC....A...G.....RGYC.G.???????
```

**Figure C: sPCA, distribution of the eigenvalues.**

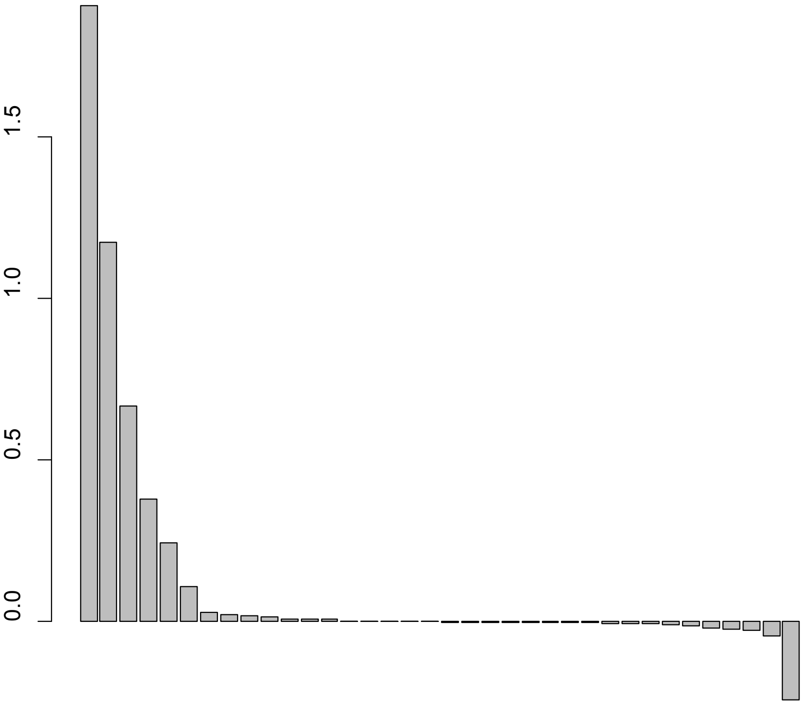

**Figure D: sPCA, spatial and variance components of the eigenvalues (screeplot).**

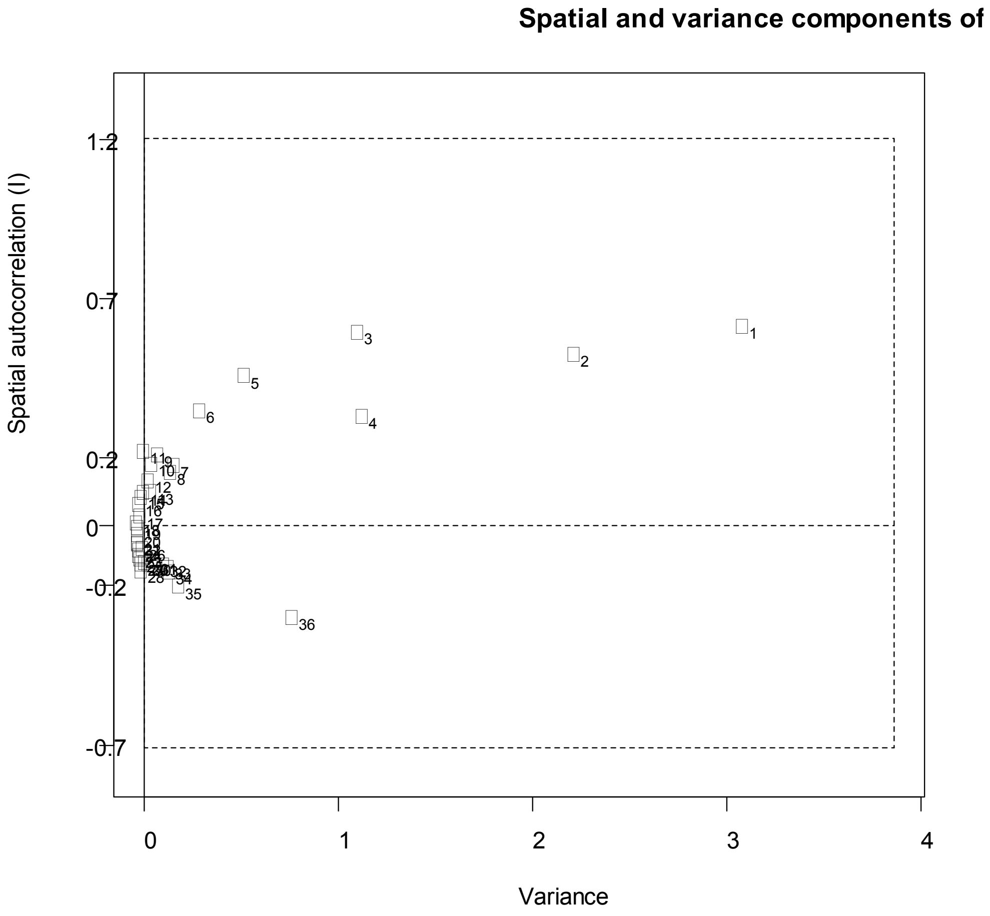

**Figure E: sPCA, distribution of the first three principal components of the sPCA for samples colored according to their origin.**

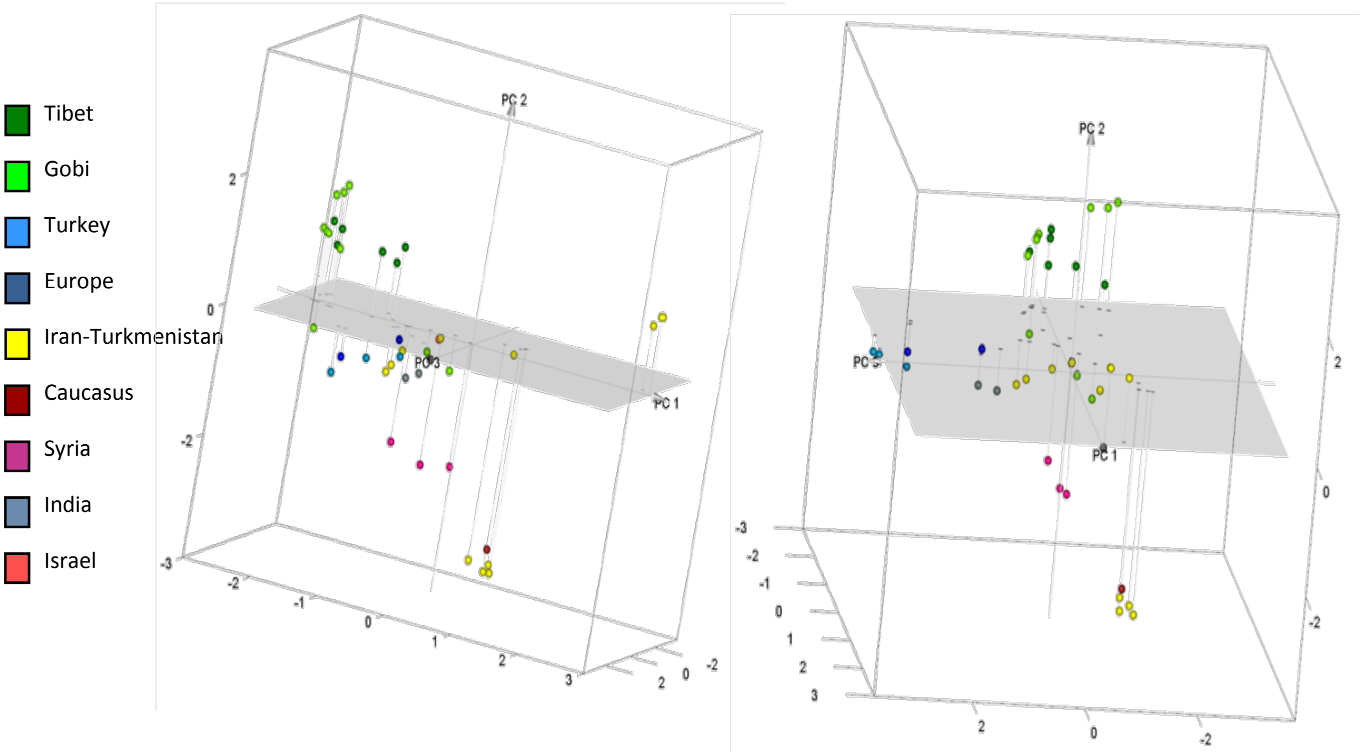


**Figure F: sPCA, histogram of the simulation to test the significance of the global structure** (Monte-Carlo test: global.rtest; based on 9999 replicates; Simulated p-value: 1e-04; Observation: 0.07736011; Std. Obs. 1.320706e+01; Expectation 2.286128e-02; Variance 1.702795e-05).

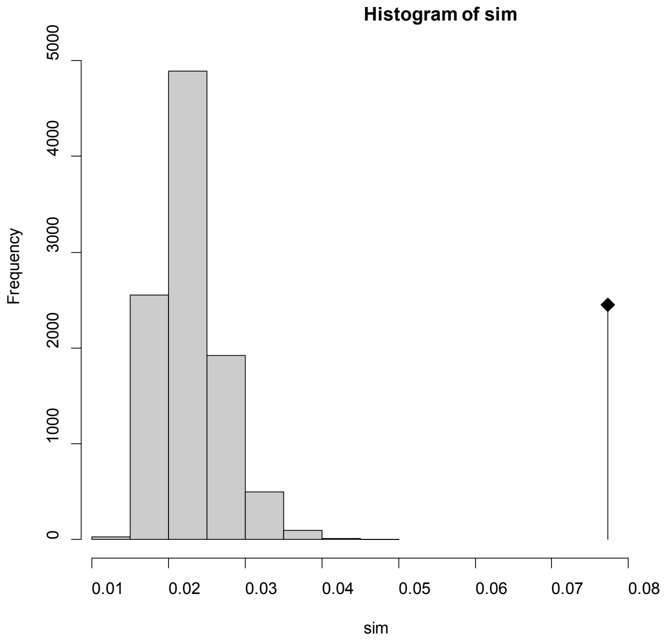

**Figure G: sPCA, histogram of the simulation to test the significance of the local structure**

(Monte-Carlo test: local.rtest; based on 9999 replicates; Simulated p-value: 1e-04; Observation: 0.03913148; Std. Obs. 3.618507e+00; Expectation 2.391413e-02; Variance 1.768558e-05). The local structure detected, even though not as reliable as the global structure, results mostly from the Mongolian and Tibetan hemiones that show the highest dispersion of the sPCA values in the last (36[th]) axis (see Table D).

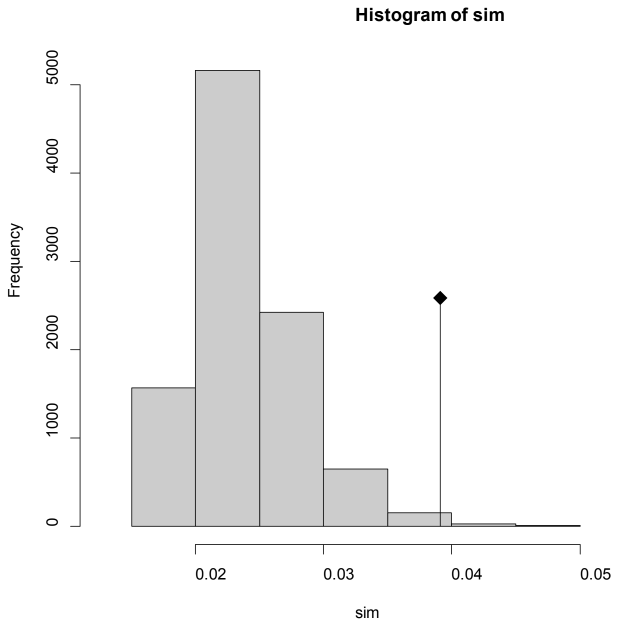

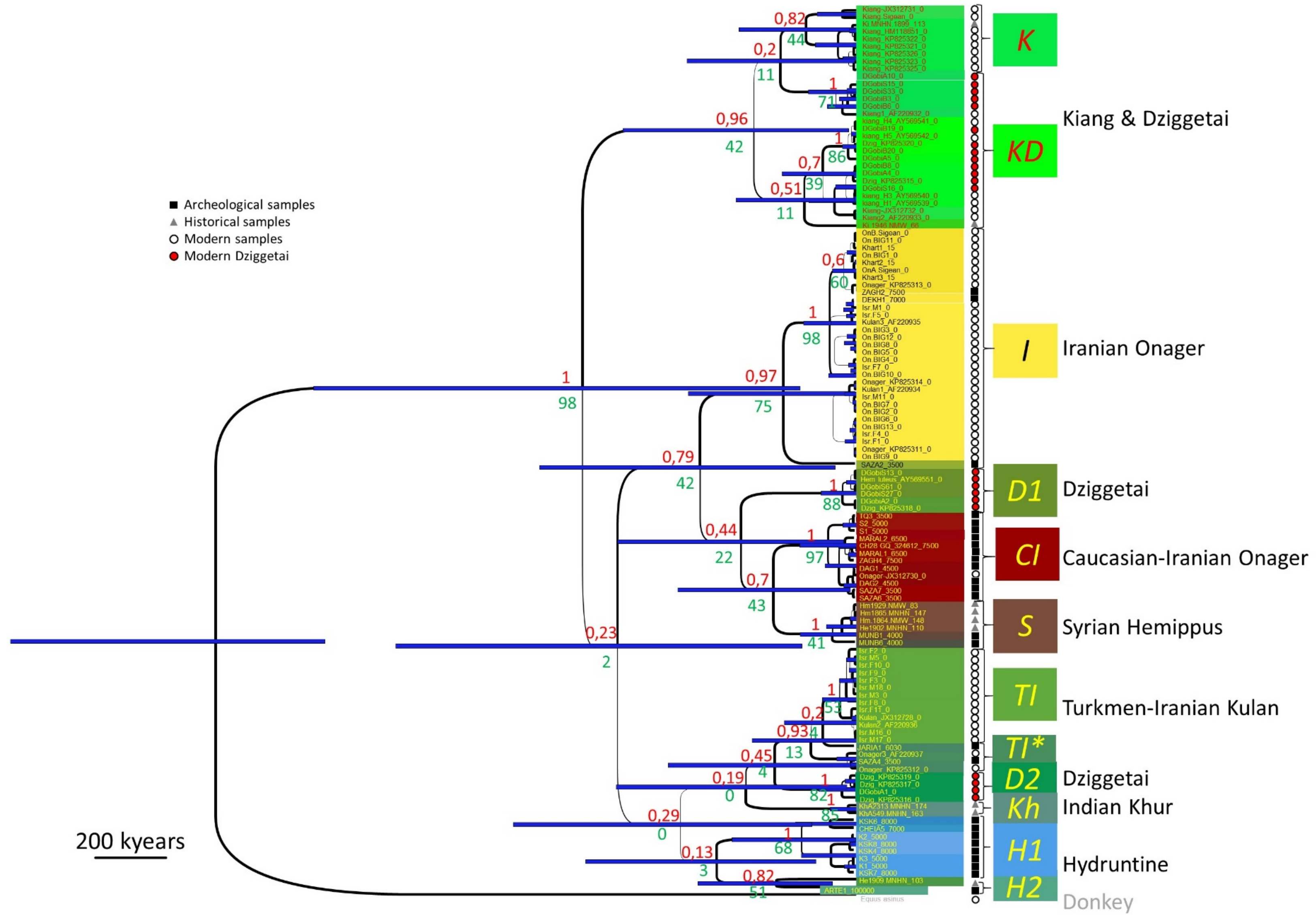


**Figure H: Phylogenetic tree of Hemione mitochondrial control region constructed through BEAST analysis.**

Enlarged view of Figure 3 with sequence names. The 95% highest posterior density (HPD) intervals of the node heights are indicated by blue bars. Posterior probabilities of the nodes are indicated in red, and the bootstrap supports of these nodes as determined by an ML analysis perfomed with RaXML [1] are in green.

| | Date (Genome) | | Date (Mitogenome) | | Date (HVR) | |
|---|---|---|---|---|---|---|
| Nodes | Median | 95% HPD | Median | 95%HPD | Median | 95%HPD |
| A | 4500 | 4500-4500 | 4270 | 3970-4730 | | |
| B | | | 2920 | 2790-3100 | | |
| C | 1982 | 1967-1995 | 2920 | 2810-3110 | | |
| D | 1733 | 1720-1745 | 2620 | 2490-2800 | 1889 | 1539-2430 |
| E | 1572 | 1556-1586 | 1750 | 665-2490 | | |
| F | 745 | 730-755 | 672 | 649-688 | 831 | 193-1570 |

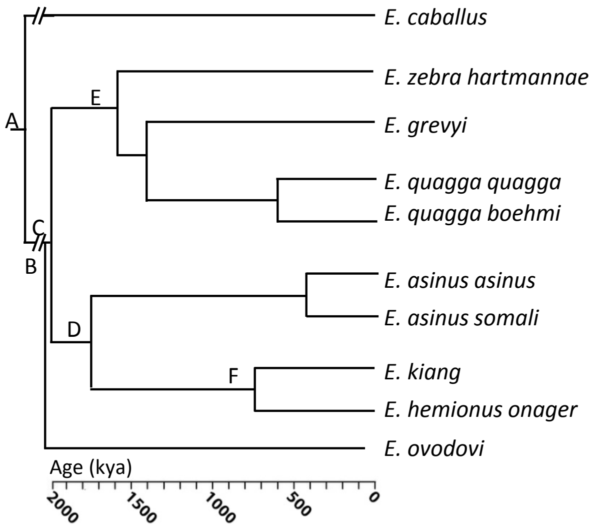


**Figure I: Dating estimates of the equine phylogeny.**
The equine phylogenetic tree as established using genomic [2] and mitogenomic data [3]. The phylogenetic position of *E. ovodovi*, a ca. 45,000-year-old Siberian *Sussemionus*, was established based only on a near-complete mitogenome sequence, since no genomic data are available. The table presents the date estimates, in kya, of the nodes (A-F) established from protein-encoding genomic data (Relaxed molecular clock, [2], mitogenomes (without outgroup, [3] and the hypervariable region (HVR) of mitochondrial DNA (this study).

## II. Short description of the samples that yielded results

**Description of the samples that constitute the clades in the phylogenetic tree of Fig. 2-4. The nucleotide positions on which the assignment to clades is based are presented in Figure A-B.**

*KD clade:*

- Sequences in GenBank of modern kiangs: AF220932 (unknown origin); AF220933 (studbook studbook 39, captured in China); AY569539, AY569540, AY569541, and AY569542, (feces from the north of Tibet); JX312732 (studbook Riga27; born in the zoo of Riga) [3-7]
- A kiang that was born in Amsterdam, died in the Viennese zoo in 1946 and was conserved in the Natural History Museum,Vienna: NMW1946
- Samples A4, A5, A10, B3, B6, B19, B20, S15, S16, S33 from extant dziggetais from the Gobi desert (feces and carcass remains, [8])
- Modern *E. hemionus hemionus* (feces from China, Xinjiang, Kalamaili): sequences in GenBank KP825315, KP825320 [7]

*K clade:*

- Modern kiangs: sequences in GenBank HM118851 (*E. hemionus luteus* from Kunlun mountains, southern Tibet); JX312731 (studbook Riga31; born in zoo of Riga; Tierpark Berlin); KP825321-825326 (feces from southern Tibet, Nyalam, Gyirong, Ngamring and Tingri reserves, [3-7])
- Kiang specimen of the 19th century conserved at the Museum of Natural History, Paris: MNHN1899-610
- Kiang from the « Réserve africaine de Sigean », France

*D1 clade:*

- Modern *E. hemionus luteus/hemionus* sequences in GenBank: AY569551 **(**feces from southwest Mongolia) [6]; KP825318 (feces from China, Xinjiang, Kalamaili, [7])
- Samples A2, S13, S27, S61 from extant dziggetais from the Gobi desert (sampled in Gobi A and small Gobi as described [8])

*D2 clade:*

- Modern *E. hemionus hemionus*: sequences in GenBank: KP825316, KP825317, KP825319 (feces from China, Xinjiang, Kalamaili [7])
- Sample A1 from extant dziggetai from the Gobi A (sampled as described [8])

*I clade*:

- Archaeological samples from Zagheh (Iran, 7-6th millennium BCE)
- Archaeological samples from Deh Kheir (Iran, Late Neolithic)
- Archaeological samples from Sagzabad (Iran, 2nd millennium BCE)
- Samples from three modern Iranian onagers collected in 1997 at Khartouran (province of Khorassan, Iran; stored at the University of Teheran)
- Samples from 13 feces of modern Iranian onagers collected in the Bahram-e Goor Protected Area (province of Fars, Iran)

- Feces samples from extant onagers from the zoological park "Réserve africaine de Sigean" (France) stemming from Iranian onagers (studbook of the onagers: parents of individuals Pers10 (#10), Stell1 (#23), Pers17 (#17), Pers23 (#44) and Pers24 (#45))
- Sequences in GenBank from Turkmen kulans kept in the zoo of San Diego, USA [9]: AF220934 (*E. hemionus* isolate kulan 1 studbook 68 SU37/Ko2, born in Kolmarden zoo from mother captured in Turkmenistan) and AF220935 (*E. hemionus* isolates kulan 3 studbook 192 SU60/Be35; born in Tierpark Berlin from mother captured in Turkmenistan)
- Sequences in GenBank from modern *E. hemionus onager* (feces from the Turan and Bahram-e Goor reserves, Iran): KP825311, KP825313, KP825314 [7]
- Samples from the wild extant Iranian hemiones reintroduced in the Hai-Bar Yotveta breeding core, Israel, stemming from wild Iranian parents, sampled between 1989 and 1991 (studbook Iran5, 7, 17, 18, Tilb2, Tilb 4)

*CI clade*:

- Archaeological samples from Shengavit (Armenia, ca. 3,000 BCE)
- Archaeological samples from Tqisbolu Gora (Georgia, ca. 500 BCE)
- Archaeological samples from Velikent (Dagestan, ca. 1,800 BCE)
- Archaeological samples from Zagheh (Iran; 7-6th millennium BCE)
- Archaeological samples from Maral Tepe (Iran, Chalcolithic to Bronze Age)
- Archaeological samples from Sagzabad (Iran, 2nd millennium BCE)
- Archaeological sample from Sagzabad that was published previously: GQ3246 [10]
- Sequence in GenBank JX312730 [3]: onager from the Reepark Ebeltoft, Denmark, born in Montpellier in 2007 from a mother captured in Iran, and died in 2012 in Reepark; studbook 818 Montpel. 33.

*S clade:*

- Archaeological samples from Munbāqa (Syria, Bronze Age)
- Sample from a 19th century specimen (*E. hemionus*) conserved in the Natural History Museum of Paris: MNHN1902
- Sample of a *E. hemippus* specimen from the 19th century conserved in the Natural History Museum of Paris: MNHN1865, C.G. 1977 n°3
- Samples of three *E. hemippus* specimens from the 19th century and the early 20th century conserved in the Natural History Museum,Vienna: NMW1864, NMW1892, NMW1929

*TI clade:*

- Sequence in GenBank AF220936 from a Turkmen kulan kept in the zoo of San Diego, USA (*E. hemionus* isolate kulan 1 from an individual born in Tierpark Berlin from mother captured in Turkmenistan; studbook 28 W SU16/Be10 [9])
- Sequence in GenBank JX312728 from an extant Turkmen kulan (specimen from the zoological garden of Kolmarden, Sweden, studbook J-Lo 1652) [3], 5th generation stemming from Turkmen ancestors (parents of SU56/Be31, studbook 188, and SU59/Be34, studbook 191)
- Samples from hemiones stemming from wild Turkmen parents reintroduced in the Hai-Bar Yotveta breeding core, Israel, sampled between 1898-1991 (studbook SU51/Be26-30)

*TI* clade:*

- Archaeological sample from JariA (Iran, ca. 4,000 BCE)
- Archaeological sample from Sagzabad (Iran, 2nd millennium BCE)
- Sequence in GenBank AF220937 from an onager kept in the zoo of San Diego, USA (*E. hemionus* isolate of an onager born in Hamburg from a mother captured in Iran; studbook Pers18; [9])
- Sequence in GenBank KP825312 (feces from modern *E. hemionus onager* from the Turan and Bahram-e Goor reserves, Iran, [7])

*Kh clade:*

- Two Indian khurs from the Rann of Kachchh (Kutch) captured in 1838 and 1849; specimens of the Natural History Museum, Paris: MNHN A2313 and MNHN A549

*H1 clade:*

- Archaeological samples from Norşun Tepe (Late Chalcolithic)
- Archaeological samples from Kösk Höyük (Turkey, 6,200 - 5,400 BCE)
- Archaeological samples from Küllüboa (Turkey, Bronze Age)
- Archaeological samples from Acem Höyük (Turkey, Bronze Age, 2,000 BCE)
- Archaeological samples from Cheia (Romania, 5,020 cal BCE)

*H2 clade:*

- Archaeological sample from the Artenac cave (France, layer dated to ca. 100,000 years ago [11])
- Sample of an Iranian onager that was born in the Paris zoo and died in 1909: MNHN1909

# III. Archaeological samples

*Foreword: Morphological determination of equid remains. Joris Peters*

Osseous and dental remains of Equids are regularly found in Holocene archaeological sites of Eurasia, but their specific identification poses a problem, particularly in regions such as the Near East where, following the Younger Dryas cold spell, the distributions of *E. ferus, E. hydruntinus, E. hemionus* and *E. africanus* showed considerable overlap. Domestication efforts during the 5$^{th}$/4$^{th}$ millennium BCE (Before Common Era) moreover gave way to two domestic forms that became widespread in Eurasia, namely the horse (*E. caballus*) and the donkey (*E. asinus*). In addition, crossbreeding between these domesticates produced two kinds of hybrids, i.e. mules (*E. asinus* ♂ x *E. caballus* ♀) and hinnies (*E. caballus* ♂ x *E. asinus* ♀), of which the first in particular was a much-esteemed beast of burden going back at least to Roman times [12]. Dental and osteological criteria combined with metrical data allow separating morphologically the different wild and domestic forms in cases where tooth or bone preservation is excellent, e.g., in Equid burials, but when dealing with heavily fragmented specimens, such as in settlement refuse, many of these remains can only be identified to the genus level.

## 1. Southwest Asia

### 1.1. Iran

The taxonomic determination of the analyzed equid remains from Iran was based on the measurements and morphological characteristics listed in Table F.

#### 1.1.1. Maral Tepe

*Marjan Mashkour*

Maral Tepe is one of the five archaeological sites of the Ozbaki archaeological zone located in the Savojbolagh plain, approximately 90 km northwest of Tehran. Human occupation of Maral Tepe dates back to the end of the 5th millennium BCE and is allocated to the Chalcolithic due to the presence of a specific pottery style know as Plum ware [13], and continued through the Bronze Age. The site seems to have been an important settlement with a developed architecture. The single existing radiocarbon date made on a bone sample provides the following measure: Gif-11837 5,275±65 BP (4,249-3,966) cal BCE 2 sigma [14]. The faunal remains of the site comprise mainly wild and domestic caprines, gazelle and equids. The equid species in the site, identified on the basis of morphometrical analysis, are *E. hemionus*, *E. asinus* and *E. caballus.*

#### 1.1.2. Deh kheir

This site is located in the northeastern Iran in an arid zone. It dates to the Chalcolithic and the Late Neolithic [15] and is one of the rare sites of this period evidencing links with southern central Asia, such as Tappeh Sang e Caxmaq [16]. Eight occupational periods were identified [17]. The general preservation state of the faunal remains is poor, being fragile and often demineralized. As a consequence, too few remains are identifiable to reliably asses the taxonomic distribution. The highest number of faunal remains is preserved in trench 4, the source of the samples analyzed genetically in the present study. According to the morphometric analyses, the equids appear to correspond to *E. hemionus* [14,17].

#### 1.1.3. Tall-i JariA and B

The sites are located in the Marvdasht Plain in southwestern Iran, in the province of Fars approximately 10 km southwest of Persepolis [18]. Mount B is a Late Neolithic site [19]. Mount A was occupied during the Chalcolithic and the Bronze Age according to radiocarbon dates obtained from

bones (Mashkour, unpublished data). The site is very rich in equid remains [20], identified at both sites as *E. hemionus*.

#### 1.1.4. Qaleh Bozi

The site is localized 25 km southwest of Isphahan and comprises two shelters and a cave on a steep slope of the Qaleh Bozi mountains at an altitude between 1750 and 1810 m overlooking the wide alluvial plain of the Zayandeh river. The site harbors Mousterian assemblages within a stratigraphic context. The faunal remains give access to the Pleistocene fauna of the Iranian Plateau. The bones show cut and percussion marks and their fragmentation is compatible with the extraction of bone marrow [21]. At least three different equid species have been identified: *E. hemionus*, *E. hydruntinus*, *E. caballus*, and another small equid with a distinct tooth morphology.

#### 1.1.5. The sites of the Qazvin plain

Three Iranian sites from the microregion Bu'in-Zahra, in the south of the Qazvin plain 140 km northwest of Teheran were studied, which are geographically close: Zagheh (formerly Tapeh Zagheh), Qabrestan, and Sagzabad [22,23]. The Qazvin plain, an arid or semi-steppe region at 1300 m altitude, is geographically limited by the Zagros mountains in the west and northwest and by the Alborz mountains in the southeast. Little is known about the faunal distribution in this area and in particular about equids, in contrast to the well studied Zagros mountains, which are not a habitat of equids, especially hemiones. This latter is the species with the highest frequency on the Qazvin plain, particularly in the three archaeological sites from the 4th millennium onwards. The high morphological diversity of the prehistoric equids in this regions gave rise to several in-depth studies [24,25]. Most of the equid species are wild and most belong to *E. hemionus*.

##### 1.1.5.1. Zagheh

The periods of occupation of the site of Zagheh delivered radiocarbon dates between the 7$^{th}$ and the 6$^{th}$ millennium BCE (between 5,212 and 4,918 BCE) and showed evidence for continuous occupation during the archaic period of the Central Plateau culture [26]. *E. hemionus* and horse (*E.* cf *ferus*) were identified in the faunal remains ([27], pp.214-219; 273-275).

##### 1.1.5.2. Sagzabad

The site is a mound with an oval shape and stretches over 350 m north to south and 400 m from east to west. Radiocarbon dates indicate an occupational period from the first to the second millennium BCE (1,264 – 1,035 BCE, Late Bronze Age to Iron Age) [26]. Wild and domestic equid species (*E. heminous*, *E. caballus*, *E. asius*, *E.* cf *hydruntinus*) were found among the faunal remains [28]. Equids represent the majority of the wild species hunted in Sagzabad ([26], pp. 273-275).

##### 1.1.5.3. Qabrestan

This Chalcolithic site dates to the first quarter of the 4$^{th}$ to the first quarter of the 3$^{rd}$ millennium BCE (radiocarbon dates of 3,782 to 3,361 BCE; [26]). The former Tell, a proto-urban site, is now flattened due to erosion [29]. Nineteen archaeological levels have been identified that correspond to four cultural periods. The faunal remains are composed of wild and domestic species, 10% of which are equid remains being leftovers of meals. Among the equid remains, the majority can be attributed to *E. hemionus*. Another more gracile morphotype was also identified in this area that is different from the present-day onager, which was classified as *E. h. binagadensis* [30]. Wild horses, donkeys and *E. hydruntinus* were also identified.

## 1.2. Caucasus

### 1.2.1. Daghestan

#### 1.2.1.1. Velikent

*Arturo Morales-Muñiz*

The site of Velikent is made up of five separate mounds 5-7 m high, situated 25 km northwest of the city Derbent (Daghestan, Russia) and 12 km west of the present-day shoreline of the Caspian Sea. The Institute of History, Archaeology, and Ethnography of the Daghestan Scientific Center of the USSR Academy of Sciences conducted excavations at Velikent from 1977 to 1979 and from 1982 to 1984. The Daghestan-American Velikent Expedition (DAV) conducted a preliminary field season in 1994 and later two field seasons in 1995 and 1997. From 1998 through 2000, yearly seasonal digs have been undertaken by the Dagesthanis [31,32].

Two mounds have been excavated. Mound II, the older one, evidenced a chronocultural sequence that spans from 3,300 to 2,800 BCE. The sequence of Mound I ranges from 2,700 to 1,800-1,700 BCE (Magomedov, pers. com.). In both mounds, a series of excavated trenches, superimposed over a square grid, uncovered a complex stratigraphy, revealing stratified fill from domestic areas arranged along a sequence of three building horizons [31,32]. These two mounds reveal two different occupation stages that have been labelled "Velikent I" and "Velikent II" since they can most properly be considered separate sites. The animal remains were systematically hand-collected rather than recovered through sieving. Only 78.5% of the Velikent I samples and 25.8% of the Velikent II samples were analyzed (in the case of the latter only samples from the two most representative collections, those from 1995 and 1998, were chosen). Despite striking sample size differences, the overall composition of faunal groups is essentially identical at both sites. In this way, mammals, domestic for the most part, constitute the overwhelming majority at Velikent I (98%) and Velikent II (95%) while birds and fish constitute marginal components. Such consistent patterns stress the homogeneous nature of the faunal remains despite the chronological, spatial and methodological heterogeneity detected. The *E. hydruntinus* samples were first identified on the basis of size and period.

In addition, the phalanx DAG2 was subject to palaeontological analysis by Véra Eisenmann (see measurements in Table G). The conclusion drawn from the measurements was that the animal was an *E. kiang* or an *E. hemionus hemionus*, although the phalanx was smaller than the average for these two species.

### 1.2.2. Armenia

*Hans-Peter Uerpmann*

Bone finds from two archaeological sites in Armenia were analyzed that belong to the Kura-Araxes cultures during the Bronze Age, when the region of the Southern Caucasus was densely inhabited.

The site of **Mokhrablur** is located in the Plain of Ararat, a region favorable for agriculture, at the left bank of the ancient bed of the river K'asakh on an artificial hillside south of Echmiadzin. The city was constructed during the second half of the 4$^{th}$ millennium BCE and experienced growth and extension from the end of the 4$^{th}$ to the beginning of the 3$^{rd}$ millennium BCE, but was abandoned during the second half of the 3$^{rd}$ millennium [33]. There is evidence of the society becoming more complex at the beginning of the Kura-Araxes period since both surface of the urban area and of the individual houses grew. Specialization of labor, mainly concerning the extraction of metal from ore and the production of ceramics, also took place during this period [33].

Similarly, **Shengavit**, situated on a hill on the right bank of Yerevan Lake, is a fortified city during the Bronze Age and was occupied during four distinct phases from the end of the 4th to the beginning of the 2nd millennium BCE [34]. The site gave its name to the corresponding culture.

#### 1.2.3. Georgia

*Hans-Peter Uerpmann and Margarete Uerpmann*

##### 1.2.3.1. Didi Gora

This site in the province of Kachetia is located on the plain of the Alazani in Eastern Georgia between the Black and the Caspian Seas which may have been a former river bank. It comprises a history of three millennia, from the Early Bronze Age (c. 2,800 BCE) to the Early Iron Age (c. 1,000 BCE) and is one of the largest hill settlements during the Neolithic and Bronze Age in this region. Archaeological evidence points to a semi-nomadic, seasonal lifestyle of the inhabitants, to the exploitation of ore deposits and to extensive trading contacts with other regions in the Near East. The Bronze Age layers are poor in archaeological artifacts except for obsidian flints, but very rich in faunal remains.

These faunal remains, which were analyzed in the present study, are very well preserved suggesting a rapid burial. Two of the genetically analyzed equid remains had been attributed to *E. hydruntinus* and three to *E. caballus* based on the relative size of the bones and, in the case of *E. caballus*, on their weight since the bones were heavily fragmented [35].

##### 1.2.3.2. Tqisbolu Gora

The site of Tqisbolu Gora was occupied between 1,800 and 800 BCE and was thus partly contemporaneous to Didi Gora. The youngest layers of Didi Gora correspond to Iron Age layers in Tqisbolu Gora. Three equid species were identified on the two sites. The bigger remains were attributed to the domestic horse (*E. caballus*) while the smaller remains could belong to *E. hemionus*, *E. hydruntinus* or to the domestic donkey *E. asinus* [35]. In the present study, six remains were analyzed genetically. Four of them had been attributed to *E. hydruntinus* and two to *E. caballus* based on the relative size of the bones.

### 1.3. Turkey

#### 1.3.1. Göbekli Tepe

*Joris Peters*

The site of Göbekli Tepe is located about 15 km northeast of the city of Şanlıurfa in southeastern Anatolia, Turkey. It is situated in the anti-Taurus foothills on a prominent hill (c. 800 m above sea level) that forms part of a range overlooking the Harran Plain. Human occupation of the Göbekli Tepe area started in the course of the Pre-Pottery Neolithic A (9,500-8,700 cal. BCE) and ended early in the Middle Pre-Pottery Neolithic B, around 8,000 cal. BCE. Architecture at the site is distinctive, consisting of large curvilinear (earlier phase) to rectangular (later phase) structures with numerous megaliths in the form of T-shaped stone pillars. The latter stand over 5 m high and have been positioned in a symmetrical arrangement (Schmidt 2006). Of interest is the fact that a fair number of megaliths show animal motifs in bas-relief, often in an almost naturalistic style. Snake, red fox and wild boar are the taxa most commonly represented, but the repertoire also includes aurochs, goitered gazelle, mouflon, equid (probably Asiatic wild ass), hare, leopard and the common crane (e.g., [36]). Apart from the representations on the pillars, large sculptures and statues of animals, mainly carnivores, have been found as well. In sum, given the monumental aspect of the site's architecture and its megalithic art, it can be safely assumed that Göbekli Tepe served a ritual purpose [37].

In the second half of the 9th millennium cal. BCE, much of Göbekli Tepe's architecture was deliberately covered with human occupation debris, a mixture of soil, lithics, stones, and animal teeth and bones. Some of the animal bones recovered from the western part of Göbekli Tepe (GTW) during the 1995 excavation campaign directed by Prof. Dr. K. Schmidt were attributed to Equids. Based on their size and morphology, the majority of the remains collected could be classified as asinine, dental or osseous remains exhibiting a caballine morphotype could not be identified in the equid assemblage. On zoogeographical grounds, von den Driesch and Peters [38] decided to classify the remains as pertaining to the Asiatic wild ass, *E. hemionus*.

### 1.3.2. Aşıklı Höyük

*Joris Peters*

Aşikli Höyük is an aceramic Neolithic settlement mound located next to the Melendiz River. It covers an area of 3.5-4 ha, rising 15.35 m above the Melendiz Ülain at the north and 13.16 m at the south. Four levels, designated as 1-4 from top to bottom respectively, were documented in a deep trench (4 GH) on the north slope. The earliest settlement known thus far at the site, represented by Level 4, has been radiocarbon dated to the second half of the 9$^{th}$ millennium BCE. Level 3 also dates to the 9$^{th}$ millennium cal BC, whereas Level 2 comprises the 8$^{th}$ millennium BCE settlement [39]. Currently, the faunal remains of the site are analyzed by a team of zooarchaeologists including H. Buitenhuis, N. Munro, J. Peters, N. Pöllath, and M. Stiner. A most important finding is the fact that sheep management was already practiced by the site inhabitants [40]. Several species of equids are present at the site. A small part of this assemblage pertains to *E. ferus*, whereas the majority belongs to hemione-like individuals, likely *E. hydruntinus* [41].

### 1.3.3. Küllüoba (Eskişehir, Turkey)

*Can Yümni Gündem, Margarete Uerpmann and Hans-Peter Uerpmann*

Küllüoba is located in the highlands of northwest Anatolia. The mound lies some 1300 m south of the village of Yenikent and the nearest city is Eskişehir. The mound is flat (9.5 to 10 m in height) and 150 by 250 m in dimension. The excavation has been ongoing since 1996 under the direction of T. Efe and the Eskişehir Archaeological Museum. The animal remains were mainly analyzed by C. Y. Gündem with contributions from H.-P. Uerpmann and M. Uerpmann. Approximately 9,800 remains (c. 92.6 kg) were identified as mammal. The material derives from four different periods, a Transition Period (3300-3000 BCE) from the Copper Age to the Early Bronze Age (EBA), EBA I (3,000-2,700 BCE), EBA II (2,700-2,400 BCE) and EBA III (2,400-2,000 BCE). A general picture from the animal remains indicates that the number of wild animals decreased over time (presumably as the occupants became less dependent on game due to the availability of livestock), while the number of sheep remains increased over time (possibly for economic reasons like the usage of milk and wool), and that cattle served as the principal meat source (while also being used as labor animals). Almost the entire array of the identified wild animals (wild horse, European wild ass, wild sheep) prefers a steppe environment with flat terrain and sparse trees/small woods (especially preferred by fallow deer) in a temperate to cold climate. The remains of European wild ass (*E. hydruntinus*) were recovered from every period. They were identified as *E. hydruntinus* by H-P. Uerpmann primarily using dental remains since bones show close similarity to the other wild ass populations.

Determination of remains in Küllüoba

AE19.81: Scapula

| | |
|---|---|
| 2 – maximum length of Processus articularis | 66.5 mm |
| 3 – length of joint surface | 48.0 mm |

4 – breadth joint surface 43.5 mm

EQHY.1: Tibia
1 – distal breadth 59.6 mm
2 – distal depth 42.1 mm

*E. hydruntinus* was determined on the basis of size and slenderness. It is likely that other hydruntines and wild horses are among the undetermined equid remains (as is the case at the neighboring site of Keskaya).

### 1.3.4. Kösk Höyük, Nigde

*Benjamin S. Arbuckle*

In the late Pleistocene and early to middle Holocene, *E. ferus* and *E. hemionus hydruntinus* have been identified [42]. For this project, loose maxillary and mandibular teeth were identified as belonging to *E. hydruntinus*, the most abundant equid in central Anatolia in the early Holocene, based on the morphological and metrical characteristics of their occlusal surfaces (following [43-45]).

Although identifying isolated equid teeth to the species level is difficult, mandibular cheek teeth of *E. hemionus hydruntinus* can often be distinguished from those of horses based on the morphology of the metaconid-metastylid valley (lingual sulcus or double knot) and the degree of penetration of the ectoflexid (external sulcus) [43-47]. In hydruntines, the lingual sulcus is generally 'V-shaped' (rather than 'U-shaped' as in horses) and the ectoflexid penetrates deeply, often coming into contact with the lingual sulcus, especially in molars. For the maxillary dentition, the shape of the protocone is the best discriminant [43]. Hydruntines are characterized by a short, triangular protocone compared to the elongate and asymmetrical protocone of horses [28,30]. In addition, the so-called caballine fold (or pli caballine), which is often present on the posterior margin of the ectoflexid in horses, is generally absent in hydruntines.

Köşk Höyük represents the remains of a small farming settlement located on the eastern margin of the broad Ereğli-Bor Plain, Niğde, and Central Turkey. Excavated between 1980 and 2009 by Ankara University archaeologists Uğur Silistreli and then Aliye Öztan in collaboration with the Niğde Museum, Köşk Höyük represents the most important Final Neolithic/Early Chalcolithic sequence (LN/EC) (levels V-II) in central Anatolia (dating from 6,200-5,400 BCE) [48-52].

The subsistence economy at LN/EC Köşk was based on agriculture and pastoralism, the latter focusing on sheep and goat husbandry and secondarily cattle herding [50,53]. In addition, wild equids were regularly hunted, as were aurochs, deer and occasionally wild boar. Barley and emmer wheat were grown, as were legumes including pea, lentil and vetch. The LN/EC occupation is characterized by crowded, small and irregular domestic structures made of both mudbrick and stone with internal hearths, platforms, and bins. The ceramic corpus is unique to this region of Cappadocia and includes primarily red and black burnished wares with prominent relief decorations including spectacular images of animals as well as humans engaged in dancing, harvesting, milking cattle, and hunting large mammals including hydruntines [49].

### 1.3.5. Norşun Tepe

*Joris Peters*

Equid remains are preserved in Late Chalcolithic and Bronze Age levels of Norşun Tepe in the Altinova plain of Eastern Anatolia, a steppe-like region characterized by continental climate. Since they have been found to be of a more robust build than Przewalski horses [54], they were assigned to *E. ferus*.

Three specimens from the Late Chalcolithic level were analyzed in the present study, a distal tibia, a distal radius and a phalanx 2 (Figure J).

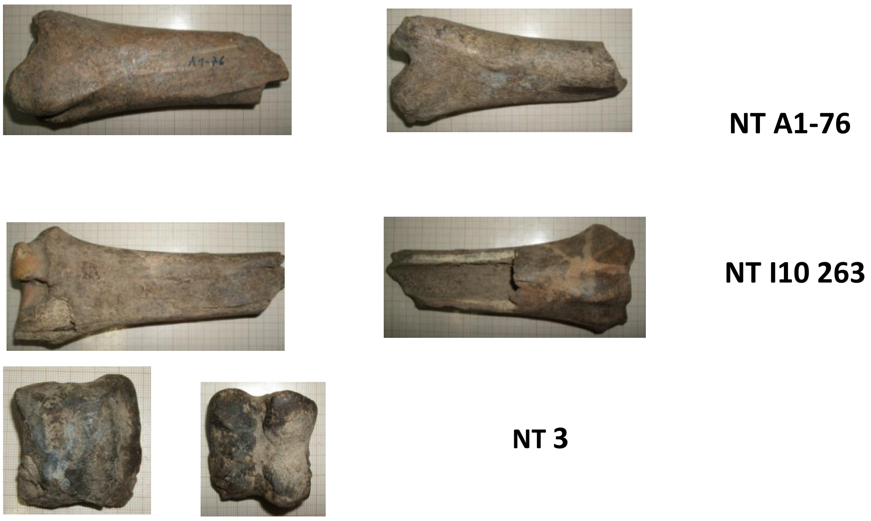


**Figure J. Specimens from Norşun Tepe.**

### 1.3.1. Acemhöyük

*Benjamin S. Arbuckle*

Acemhöyük is a large mound site located in the Aksaray province of central Turkey representing an important urban center in the Early and Middle Bronze Age. It sits at an elevation of 950 m above sea level on the alluvial fan of the Melendiz river near the southwestern tip of a large salt lake (Tüz Gölü). In addition to its location in proximity to arable alluvial soils, the site is situated on the eastern edge of a series of semi-arid connected basins consisting of the Konya-Eregli-Bor plains - a region known as the Axylon (treeless steppe) in the Classical period [55,56]. The central mound is approximately 800 x 600 meters in dimension rising 20 meters above the surrounding Yesilova plain. The site has been excavated since 1962 by N. Özgüç, DTCF, Ankara University in association with the Turkish Historical Society and the General Directorate of Antiquities and Museums and by A. Öztan of the same institution since 1989 [55-57].

Acemhöyük consists of twelve levels with deposits representing Chalcolithic to Medieval occupations [58]. Equid material for this project derives from levels XI and V. In Level XI, Acemhöyük represents an early urban center of the late Early Bronze Age and is radiocarbon dated primarily to the second half of the 3rd millennium BCE. Level V represents the early Middle Bronze Age and is radiocarbon dated to c. 2,000 BCE.

### 1.3.2. Troy

*Hans-Peter Uerpmann*

The excavation of the site of Troy, located close to the Dardanelles under the hill of Issarlik in western Turkey, has identified a succession of 9 towns and 46 occupational levels. The complex stratigraphy extends over 20 m. The bones used for the present study originate from three different levels, Troy I, Troy IV and Troy VI. Troy I is a small fortified village and corresponds to the first human occupation

of the site during the Early Bronze Age. Troy IV is dated to the Middle Bronze Age and corresponds to the phase of decline of the town whose subsistence mode seems to have been based on agriculture and handcraft rather than trading. Troy VI corresponds to the golden age of the town at the end of the Bronze Age as witnessed by the construction of palaces.

Only a fraction of the animal remains found during the ongoing excavations have been studied. Nevertheless, the archaeozoological data of more than 120,000 bone finds are stored in a database [59].

#### 1.3.3. Lidar Höyük

*Joris Peters*

Southwest of the village Lidar (southeastern Anatolia, Turkey) lies the impressive settlement mound of Lidar Höyük, measuring 200 by 240 m at the base and rising 23 m above the Euphrates River terrace. Situated near a major passage across the Euphrates River, the site of Lidar Höyük was already inhabited in the Early Bronze Age. Although occupation lasted until Medieval Times, the archaeological record shows the site was not permanently occupied.

Excavations carried out between 1979 and 1986 under the direction of Prof. Dr. H. Hauptmann produced a rich archaeofauna totalling 62,453 vertebrate remains [60]. 3.8% of this assemblage pertains to equids, remains of donkeys outnumbering those of horses and hemiones. Difficulties arose to assign some larger specimens either to (small) horse, mule or Asiatic wild ass [60]. The 1987 excavation produced another sample of equid bones, from which specimens were selected for ancient DNA analysis, including a Bronze Age horse radius collected in Area S47.

### 1.4. Syria

#### 1.4.1. Tall Munbāqa/Ekalte

*Joris Peters*

The site of Tell Munbāqa/Ekalte (36° 22 N, 38°13 E) is situated on the east bank of the Middle Euphrates in northern Syria. Its location in an area with important trade roads and Euphrates passages connecting the Mediterranean region with the Middle East may explain why people have settled here since prehistoric times. Excavations carried out by Prof. Dr. D. Machule in the ruins of Tall Munbāqa/Ekalte revealed that site occupation started in the Early Bronze Age (c. 2,500 BCE) and continued well into the Late Bronze Age (1,600-1,200 BCE), when the city prospered. In 1,458 BCE, Tell Munbāqa/Ekalte was burned down during the military campaign of the Egyptian King Thutmose III.

Excavations in the 1980s produced numerous faunal remains (>20,000). The bulk of the archaeofauna dates to the Late Bronze Age. So far, detailed accounts have been published only for two smaller assemblages [61,62]. From these it can be seen that equid remains are quite common in the assemblage. Most of these pertain to donkeys, but there is evidence for horses and hemiones, too. The equid bones selected for ancient DNA analysis originate from Late Bronze Age layers. They had been collected in different areas of the excavation site, so it is likely that these specimens represent different individuals.

**Morphological characterization of Late Bronze Age *E. hemiones* from Syria and historical *E. hemippus* specimens**

Osteometrical comparison was performed based on breadth measurements of postcranial skeletal elements of historical *E. hemionus hemippus*, including the individual housed in the National Museum

of Natural History in Paris (MNHN), measured by Véra Eisenmann and analyzed genetically in the present study, and the Late Bronze Age specimens collected at Tall Munbāqa/Ekalte on the Middle Syrian Euphrates, measured by the author (Table H). The specimen from the MNHN, a female skull (No. 1865-101), died on May 8, 1865 in the "menagerie" of Paris [63]. Its skull measurements have been compared with those of four other known specimens housed respectively in the MNHN in Paris (1863-20; female), the Natural History Museum London (1867-12-3-1; female), the Peabody Museum, Yale University, New Haven, Connecticut (No. 1637, male) and the Museum of Comparative Zoology, Cambridge, Massachusetts (6345, female). Several characteristic features distinguish the skulls of the hemippi from those of donkeys. Morphometrically they resemble more closely the kulan and the khur from Turkmenistan and India respectively, and less the Iranian subspecies *E. hemionus onager* [63].
In the present study, the specimens He1863-20 and He1902 from the MNHN in Paris and three specimens from the Natural History Museum in Vienna (Hm1929.NMW 5493/B 3625; Hm1892.NMW 1308/B4690; Hm1864.NMW 6048/ST345) were analyzed genetically.

In the ancient Near East, equids were once widely distributed. At least three species have been evidenced, of which the wild horse (*E. ferus*) can be recognized quite easily due to its comparably large size and its particular dental and postcranial morphology. Distinguishing African wild ass (*E. africanus*) from broadly similar-sized Asiatic wild ass (*E. hemionus*) is clearly more complicated, but some morphological and osteometrical criteria can be found in the literature (e.g. [25,64,65]). However, specimens retrieved from archaeological contexts often have been intentionally fragmented to access the bone marrow or have been damaged after being deposited or during excavation, complicating a specific identification.

The issue addressed here centers on bone (and hence body size and weight) in *E. hemionus* in order to detail the species' size development in prehistoric as opposed to modern times. We have compared osteometrical data from hemiones hunted during the 10$^{th}$-9$^{th}$ millennia BCE in the middle Syrian Euphrates valley at Tell Mureybet (data kindly provided by L. Gourichon and D. Helmer) and at the headwaters of the Balikh at Göbekli Tepe, 150 km further north in southeastern Anatolia with measurements obtained on Bronze Age (2$^{nd}$ millennium BCE) specimens from Tell Munbāqa on the middle Syrian Euphrates valley (this study) and their homologues in the modern Syrian onager, *E. hemionus hemippus* (published by Eisenmann). Based on the distal articular breadth (BFd) of the radius and the 3$^{rd}$ metacarpal as well as the greatest breadth (GB) of the astragalus, it can be seen that the *E. hemionus* hunted at Tell Mureybet were on average smaller than their relatives living near Göbekli Tepe. The single Munbāqa specimen considered here, a distal radius, fits the median size of the hemiones from nearby Tell Mureybet. As to the modern Syrian *E. hemionus hemippus*, its bone size either corresponds to the lower range or smaller of the animals from Tell Mureybet and Göbekli Tepe.

For the Logarithmic Size Index analysis, the following breadth measurements of the female *E. h. hemippus* No.6345 (Museum of Zoology, Cambridge) were considered: Humerus Bd, Radius BFd, Metacarpal BFd, Metatarsal BFd, and Astragalus GB. The values obtained in No.6345 served as a baseline (= 0-value in the graph) for comparison with those in early Neolithic *E. hemionus* from Göbekli Tepe and Tell Mureybet (Figure K-L).

Applying this method, the difference in average bone size of the two sub-populations implies that the hemiones hunted near Göbekli Tepe were on average clearly heavier than their relatives living further south. In this respect, landscape and vegetation near the headwaters of the Upper Balikh likely offered more favorable living conditions to *E. hemionus* since this area received more than 400 mm of precipitation per year compared to ca. 200 mm for the Middle Syrian Euphrates Valley near Tell Mureybet. It is therefore possible that we are dealing with two subspecies. Following actual vegetation

zones, the larger subspecies were associated with the xerophilous deciduous steppe-forest of *Quercetea brantii*, whilst the smaller one was confined to the Mesopotamian steppes of the *Artemisieta herbae-albae mesopotamica* ([66], Map 7).

The small size of *E. h. hemippus* dates presumably to the historical period. Destruction of vegetation by overgrazing of livestock and the increased anthropogenization of the landscape in historical times certainly caused fragmentation and reduction in numbers of the hemione population. Their survival under poor conditions may be the reason for the small size observed in animals captured prior to the subspecies' extinction in the early 20$^{th}$ century AD.

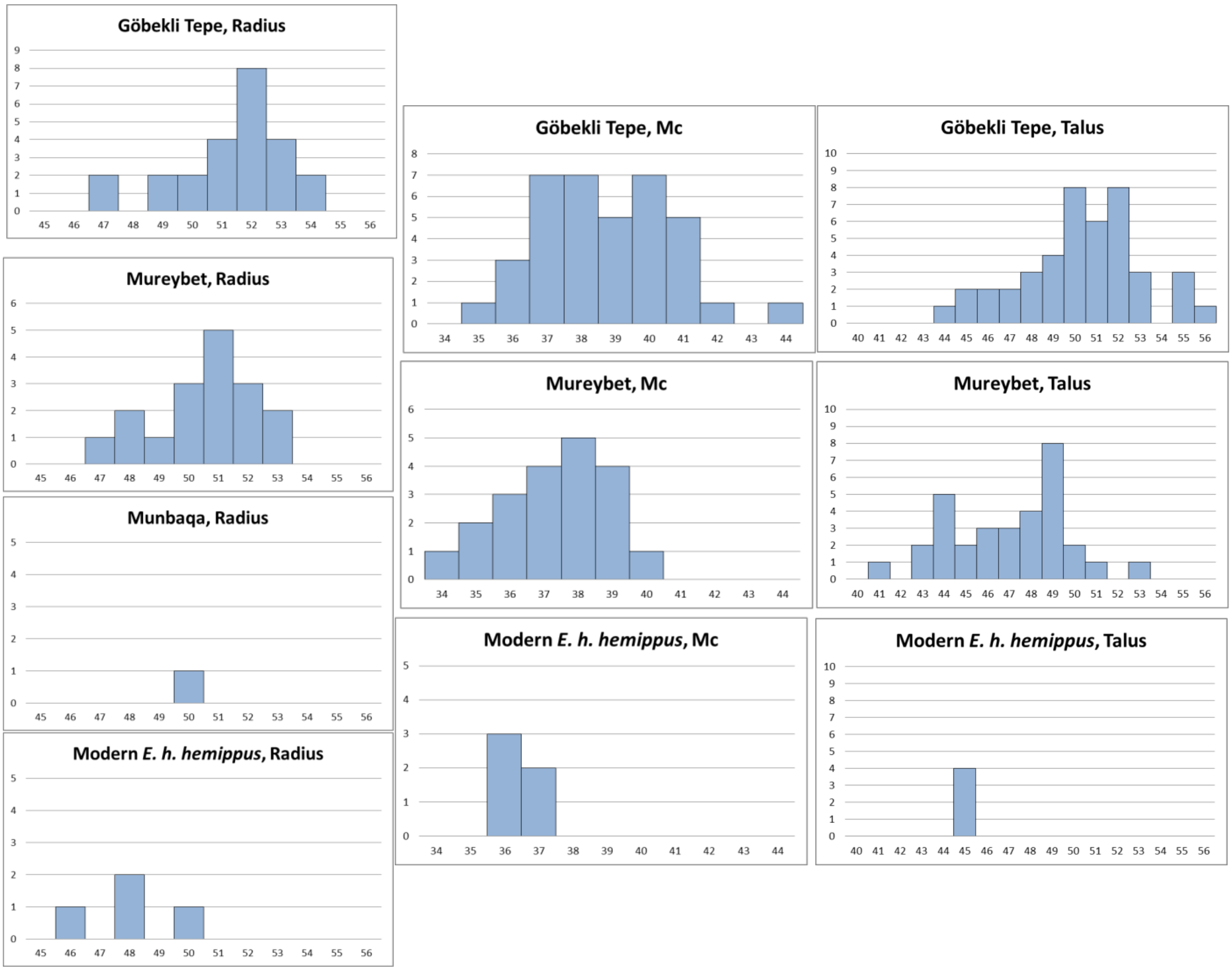


**Figure K: Osteometrical data from Syrian hemiones.**
Osteometrical data from hemiones hunted during the 10$^{th}$-9$^{th}$ millennia BCE in the middle Syrian Euphrates valley at Tell Mureybet (data kindly provided by L. Gourichon and D. Helmer) and at the headwaters of the Balikh at Göbekli Tepe in SE Anatolia have been compared with measurements obtained on Bronze Age (2$^{nd}$ millennium BCE) specimens from Tell Munbāqa in the middle Syrian Euphrates valley and their homologues, the modern Syrian onager, *E. hemionus hemippus* (published by V. Eisenmann). BFd=Distal articular breadth of the radius and the 3$^{rd}$ metacarpal (McIII); GB=greatest breadth of the talus. X axis = values (mm); Y-axis = frequency (number of bones).

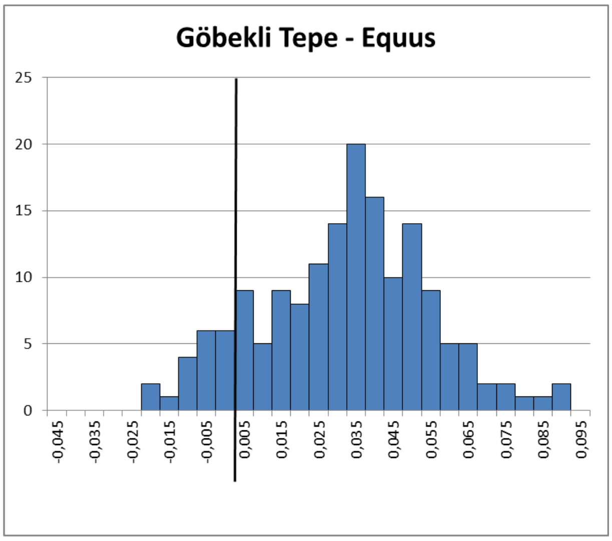


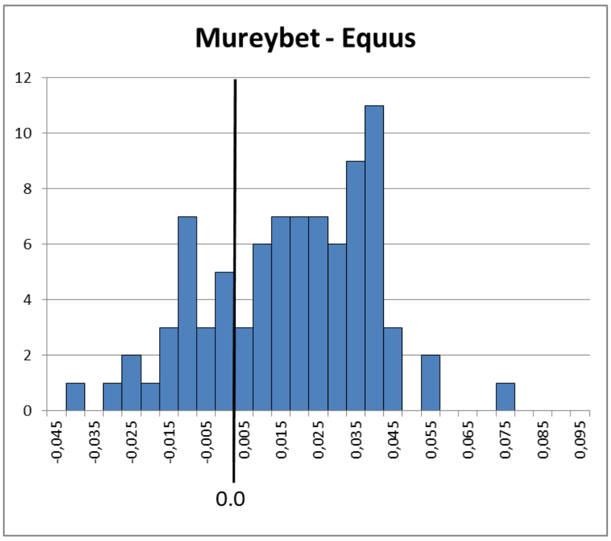


**Figure L: Logarithmic Size Index method for Syrian hemiones.**
Measurements included are: distal articular breadth of the humerus, distal articular breadth of the radius, distal articular breadth of the metacarpal, distal articular breadth of the metatarsal, and greatest breadth of the astragalus. Logarithmic size comparison between *E. hemionus* from Göbekli Tepe and Tell Mureybet. 0-value = the measurements from the female *E. h. hemippus* No.6345 (Museum of Zoology, Cambridge).

## 2. Europe

### 2.1. East Europe

#### 2.1.1. Cheia (Romania)

*Adrian Bălăşescu*

The site of Cheia (44º30'51.22"N; 28º25'59.77"E; alt. 48 m) was discovered in 2001 in the center of the village of Cheia (dep. Constanta), on the left bank of the river Casimcea on a terrace [67]. The site is located in a microregion with a diversified landscape: the grasslands of the Casimcea River, and the karstic zone of the central hills of Dobrodgea ("Cheile Dobrogei"). The extension of the site is limited to a terrace plateau with a height of 20 m at the side of the river, 500 m on the east of Casimcea, 600 m northeast of the cave "La Izvor" and 600 m south of the valley of the Sabla. A radiocarbon date yielded $^{14}$C - cal BCE 5,020- 4,797 (2 sigma) [67].

The faunal remains are numerous (around 4,000 remains of mammals) and diversified. The remains of domestic animals predominate (89%) which suggests that husbandry was an important activity. Among the domesticates, cattle represent 49.4% and caprines 39.5%. No pigs were found [68-71]. Hunting was of minor importance since only 11% of the remains are from wild animals representing 15 species, among them are red deer (2.22%), hydruntine (1.91%), fox (1.63%), aurochs (1.61%) and wild boar (1.45%).

The analyzed specimens all come from a single stratigraphic unit (US 3094) representing a habitat structure that was rich in ceramics, faunal remains and artifacts (flint and bone tools etc) [71]. The fauna that was analyzed shows the characteristics of the debris of a neolithic community: butchering marks, burning marks, carnivore marks, etc.

The analyzed specimens, three teeth and four long bones were determined as *E. hydruntinus* according to [72]. The jugal teeth were measured at the level of their occlusal surfaces. The two superior molars were measured in the middle of their height since they show very little tooth wear. The teeth were assigned to *E. hydruntinus* on the basis of the measurements shown in Table I**.** The teeth are small, the protocone of the upper teeth is short while the ectoflexid of the lower teeth is profound. The dimensions are in agreement with the assignments to this species of bones from sites in other regions of Romania (Dobroudja) [73,74]), France, Azerbaïdjan, Crimea and Iran (e.g., [30,75,76])

#### 2.1.2. Svodin (Slovakia)

*Hans-Peter Uerpmann*

The archaeological site of Svodin lies 16 km north of the Danube in the region of the Hron river and was excavated from 1979-1983 [77]. It was a settlement of the Lengyel- and the Baden cultures dating to ca. 4,900-4,300 BCE and 3,500-2,800 BCE, respectively. Other stratigraphic layers and remains concern the Palaeolithic, the Bronze Age, Hallstatt and Slavic settlements. The site lies in a deep Loess layer up to 40 m thick.

The Bronze Age on the territory of Slovakia went through three stages of development, stretching from 2,000 to 800 BCE. Major cultural, economic, and political development can be attributed to the significant growth in production of copper, especially in central Slovakia (for example in Špania Dolina) and northwest Slovakia. Copper became a stable source of prosperity for the local population. After the disappearance of the Čakany and Velatice cultures, the Lusatian people expanded the building of strong and complex fortifications, with large permanent buildings and administrative centers. Excavations of Lusatian hill forts document the substantial development of trade and agriculture at that period.

The richness and the diversity of tombs increased considerably. The inhabitants of the area manufactured arms, shields, jewelry, dishes, and statues. The arrival of tribes from Thrace disrupted the people of the Calenderberg culture, who lived in the hamlets located on the plain (Sereď), and also in the hill forts located on the summits (Smolenice, Molpí). The local power of the "Princes" of the Hallstatt culture disappeared in Slovakia during the last period of the Iron Age after strife between the Scytho-Thracian people and the Celtic tribes, who advanced from the south towards the north, following the Slovak rivers.

The victory of the Celts marked the beginning of the late Iron Age in the region. Two major Celtic tribes living in Slovakia were Cotini and Boii. Cotini were probably identical or made significant part of so-called Púchov culture. The Celts built large oppida in Bratislava and Liptov (the Havránok shrine). Silver coins with the names of Celtic kings, the so-called Biatecs, represent the first known use of writing

in Slovakia. Celtic dominance disappeared with the Germanic incursions, the victory of Dacia over the Boii near the Neusiedler See, and the expansion of the Roman Empire.

In Central Europe, the Iron Age is generally divided into the early Iron Age Hallstatt culture (HaC and D, 800-450 BCE) and the late Iron Age La Tène culture (beginning in 450 BCE). The Iron Age ends with the Roman Conquest.

The specimen SVOD-EQ01 SVLE5104-61 was $^{14}$C-dated ($^{14}$CHRONO center, Queens University, Belfast, Northern Ireland) to 3,042±25 BP (2 sigma 95.4%: 1395 – 1218 cal BCE) in the framework of the present study.

#### 2.1.3. Gomolova (Serbia)

*Hans-Peter Uerpmann*

Gomolova, a prehistoric and early historic dwelling mound, is located on the left bank of the Sava river ca. 60 km northwest from Belgrade [78]. The stratigraphy was not clearly established, and the economy, stockbreeding, hunting, agriculture and ecology were not well studied since the main goal of the excavation was the establishment of the sequence of the cultures [78]. The oldest occupational layer belongs to the Vinča period, the youngest to a Medieval settlement and necropolis [78]. The large houses indicate that the Vinča farmers were sedentary due to an agriculture and stock-breeding-based economy [78]. Most of the faunal remains were found in pits representing garbage, and only a small proportion were found in the houses [78]. The well-preserved but heavily fragmented faunal remains from the Vinča period were studied by A. Clason and H.-P. Uerpmann. Most faunal remains belong to domestic livestock such as cattle, sheep/goat, pigs, but there are also remains of red deer, wild boar, roe deer, and aurochs [78]. A small number of equid remains were found in layers of both periods, the Vinča and the La Tène period [78]. The bones were often hacked into small pieces or partly devoured and the epiphyses gnawed away by dogs, who were the main scavengers [78].

Radiocarbon dating ($^{14}$CHRONO center, Queens University, Belfast, Northern Ireland) of specimen "GOMO-EQ01 Gomlav29/77" yielded 2,157±25 BP (2 sigma 95.4%: 356 – 106 cal BCE). Attempts to radiocarbon date the specimen "GOMO-EQ02 Gom12-278/71" failed.

### 2.2. North West Europe

#### 2.2.1. Belgium

##### 2.2.1.1. Spy

*Mietje Germonpré*

The Pleniglacial Spy Cave is one of the richest Palaeolithic sites in Belgium. It is located about 18 m above the Orneau (a tributary of the Sambre) in the Meuse basin [79]. The cave was first excavated in 1879 [80] and in 1885 two Neanderthal skeletons were discovered [81]. Since then, many excavations have been carried out [82]. Three main archaeological and bone-bearing horizons are recognized [80,81,83], although each level probably represents a palimpsest. The most important Palaeolithic industries can be assigned to the Gravettian, Aurignacian and Mousterian, including the Lincombian-Ranisian-Jerzmanowician techno-complex [84].

A wide spectrum of Pleistocene species is present. Caballine horse, cave hyena, mammoth, woolly rhino and reindeer are the most common taxa. Hyena scavenging activities are indicated by the gnawed mammoth and rhino postcranial bones and cervid antlers. Bears used the cave to hibernate evidenced by vestiges of cubs, and female and male adult bears. Indications of human manipulation (cut marks, ochre traces, worked bone/tooth) occur especially on remains of foxes, mammoth and deer. The age profile of

the mammoth is dominated by calves younger than five years. This selective mortality suggests that they were hunted by prehistoric people. AMS dates range from ca. 23,700 BCE to ca. 42,400 BCE. The Spy bone assemblage has therefore been accumulated through a series of agents over a long period of the Pleniglacial [85].

At Spy, *E. ferus* is present with 4494 Number of Identified Specimens (NISP), corresponding to 39% of the total quantity of the faunal remains. The shoulder height of the Spy caballine horses was around 140 cm, typical of the Belgian Pleniglacial horses (Germonpré et al., 1993). *E. hydruntinus* occurs with only 39 NISP, or 0.34% of the total fauna. A tooth from *E. hydruntinus* discovered in the Intermediate Level has an AMS age of ca. 32,600 BCE [86,87].

Four teeth from Spy yielded a genetic result: one permanent incisor ($I_3$) and three jugal teeth ($P_2$, $P_3$/$M_2$, $M_3$). The identification of the teeth, which are much smaller than the teeth of the contemporaneous *E. ferus*, is based on the description, figures and measurements in Stehlin and Graziosi [75,88,89].

### 2.2.2. France

#### 2.2.2.1. Artenac (Saint-Mary, Charente)

*Jean-François Tournepiche*

The cave of Artenac, North of the karst of La Rochefoucauld in the valley of the Bonnieure constitutes a thick layer of Pleistocene sediments (25 m) that is rich in palaeontological and archaeological remains [90]. Layer 10, which contains bones accumulated by cave hyenas, provided a metapodial which was attributed to *E. hydruntinus*. The age estimation of the deposition is based on a biochronological analysis of micromammals and U:Th dating of the stalagmite floor. The floor below yielded radiometric dates of 108,000 (+10,000/- 9,000) and 95,100 (+/- 900) BCE. The bone accumulation is therefore younger. The accumulation of the bones of large mammals is diversified and typical of hyena dens *(E. caballus cf. Germanicus, E. hydruntinus, Bos primigenius, Cervus simplicidens, Crocuta crocuta spelaea, Panthera spelaea, Lynx sp., Ursus sp., Canis lupus, Vulpes vulpes, Mustela putorius).* The association indicates a temperate environment but is lacking species that are associated with a temperate climate maximum (i.e., the interglacial *stricto sensu).* The chronological indications are small deer, a species known from the beginning of the Upper Pleistocene (isotope stade 5). The hyena remains show very small teeth compared to other European fossil hyenas, which might indicate that the temperate period was sufficiently long to have an effect on the dental morphology of these carnivores [91]. This reinforces the hypothesis that layer 10 is from the beginning of the Early Upper Pleistocene (the temperate phase at the beginning of the Würm close to the interglacial).

A metacarpal from this layer was subject to a palaeontological analysis performed by Véra Eisenmann (measurements reported in Table J) and resulted in a clear-cut assignment to *E. hydruntinus* (Figure M).

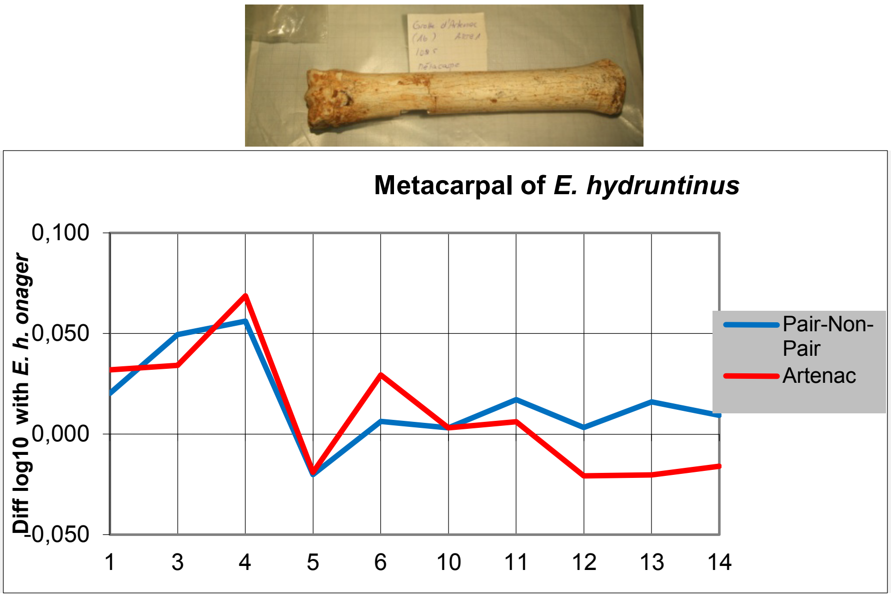


**Figure M: Simpson diagram corresponding to the comparative measurements of the Artenac metacarpal** (pictured above) given in Table J **[92].**

### 2.2.2.2. Le Quéroy (Chazelles, Charente)

*Jean-François Tournepiche*

The caves of the Quéroy constitute a large underground network that developed under a karstic plateau overlooking the valleys of the Bandiat and of the Echelle in France. The hall containing the remains is a sink-hole roughly 10 m deep, the access of which is a vertical shaft [91]. During the end of the Pleistocene, this sink-hole constituted a sink-trap. The fauna from the principal layer is homogenous and consists of gregarious herbivore species of the cold and dry Dryas I period. Two radiocarbon dates yielded 10,800 BCE and 10,590 BCE. The species present are *E. caballus arcelini* GUADELLI, *E. hydruntinus* REGALIA, *Rangifer tarandus* L., *Saïga tatarica* L., *Bovinae* ind., *Vulpes vulpes* L., *Alopex lagopus* L. The bones are often complete and well preserved, as often is the case in sink-traps. The remains of *E. hydruntinus* that were analyzed in the present study belonged to an adult and a juvenile animal. Several bones of the same individual were found, with an appendicular skeleton in loose anatomical connection.

### 2.2.2.3. The caves « Saulges » (« Grotte de Rochefort » and « Grotte de la Chèvre ») (Mayenne)

*Pierre-Elie Moullé*

These two caves are located in the Erve valley in the North West of France [93,94]. The calcareous environment ensured the preservation of skeletal remains. They contain a Palaeolithic sequence covering the Middle Paleolithic (100,000-40,000 years BCE), the Upper Paleolithic (Aurignacian, Gravettian, Solutrean and Magdalenian periods) from ca. 40,000 to 8,000 years BCE, and a Neolithic and historical sequence (galo-roman to medieval periods). The "Grotte de la Chèvre" also yielded mammal fossils from the Middle Pleistocene, although not in their stratigraphic context [95,96], as well as artifacts from the Lower Paleolithic. Five equid remains were analyzed in the course of the present study: (i) an upper molar (M1) from the “Grotte de la Chèvre” that was attributed to *E. altidens* for anatomical reasons and having a size that is similar to the molars of *E. altidens* from Venosa [97]. *E. altidens* is considered to be typical from the second half of the Early Pleistocene to the beginning of the Middle Pleistocene. A taxonomic assignment to *E. hydruntinus* from the Upper Pleistocene can be excluded. (ii) A lower molar

(M1 or M2) from "Grotte de la Chèvre" was also attributed to *E. altidens*. (iii) An upper premolar (P2) preserved in the trench “Maillard” of the “Grotte de la Chèvre” was attributed to *E. süssenbornensis*, an equid characteristic of the beginning of the Middle Pleistocene. (iv) The attribution of another upper premolar P3 or P4 from the “Grotte de la Chèvre” to *E. süssenbornensis* remains a possibility but could not be ascertained with certainty. (v) A lower molar (M1 or M2) from the “Grotte de Rochefort” was exhumed aseptically from the Solutrean layer 4.3 and immediately frozen. Its genetic analysis was performed on the unwashed remains, prior to the palaeontological analysis, which was carried out after the paleogenetic analysis and cleaning of the tooth with a damp brush. Its morphology, in particular of the double knot, corresponds to *E. ferus*.

Measurements (taken according to [98], see Figure N) of the inferior jugal tooth from the “Grotte de Rochefort” attributed to *E. ferus:*
Height (partial – since the lower part of the root was sampled for the paleogenetic analysis): 50 mm
Occlusal length (DMD): 26 mm (measurement on the enamel)
Occlusal width (DVL): 15.5 mm (measurement on the enamel)
Occlusal double knot length (DB): 14.5 mm
Occlusal postflexid length (PF): 10.5 mm

The dental measurements of the lower cheek tooth were taken according to [98]:

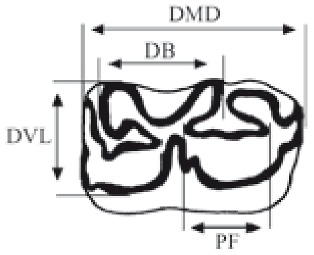


**Figure N: Occlusal view of inferior jugal tooth from the “Grotte de Rochefort”, attributed to *E. ferus*:** DMD: mesiodistal diameter (length); DV: vestibulo-lingual diameter (width); DB: double knot length ; PF: postflexid length.

### 2.3. South West Europe

#### 2.3.1. Spain

##### 2.3.1.1. Morra del Quintanar

*Arturo Morales-Muñiz*

Morra del Quintanar is a Bronze Age fortified town from SE Iberia close to the present-day city of Munera (province of Albacete, Spain). The fauna, totalling several thousand fragments, remains unpublished although a preliminary report providing an overview was published in 1991 [99]. Three stages have been detected on the basis of archaeological and $^{14}$C data. Of these, *E. hydruntinus* remains were retrieved in the defensive walls of the city (Muralla, Corte 1) that correspond to the earliest occupation stage (Phase I) dated to the middle of the 17th century BCE [UGRA 101: 3,610+/-130 cal. BP (1,660 +/-130 BCE)]. The accompanying fauna from this Corte 1 level, as is the case for all the samples from the wall sections, evidence a poor state of preservation, with extensive burning and few, if any, gnaw marks. These data convey the impression that the remains were rapidly discarded and buried after being burned. The remaining faunal elements at the Corte 1 include: horse (22), cattle (21), goat (5), domestic ovicaprines (25), pig (1), dog (2), rabbit (11), hare (2), wild boar (9), and red deer (23). This assemblage, where wild animals represent close to 40% of the sample (conceding that the horses

were domestic and the rabbits hunted and not intrusives) is quite distinct from both the area outside of the fortified town, where rabbits (probably intrusives) dominate, and the inner town, where domestic stock, mainly bovids, constitute the most common item. Also, because the state of preservation in the two later areas is excellent, it appears that the fauna from the defensive wall represents a different kind of assemblage, where hunting was common (i.e., a different sector of the population from the inner town such as soldiers).

The bones inferred to represent wild asses were taken so on account of three lines of evidence: (i) Size. Since all the bones derive from adult specimens, the small size of these equids contrasts with the larger size of the remaining equids, whose domestic status remains to be seen (in particular, the distal breadth, Bd, of the radius (55.5) of the presumed *hydruntinus* falls well below those documented for the horses (Bd: 68.5-73)). (ii) Chronology. The appearance of the domestic ass in the Iberian Early Iron Age (8th century BCE) precludes the possibility that the Morra de Quintanar equids of small size represent domestic asses. It should be mentioned, however, that there is a recent report of a single donkey tooth from the Chalcolithic [100]. (iii) Context. As stated, the retrieval of these remains in the only sector of this site where hunting seems to have been of any importance, lends support to the idea that the animals could represent wild specimens.

## 3. Specificity Index of samples initially determined as *E. hydruntinus*

Taxonomic identification of extinct species is a delicate art, relying on distinctive morphological and metrical characteristics of mostly isolated bones and teeth. Morphologists are also constrained to extrapolate past morphological diversity from present diversity. When complete skeletons are available, species identification can be based on firmer ground, but these are extremely rare in archaeological sites [72]. A few bones may have features that are characteristic enough to allow consensual assignment to an extinct species by different osteologists, while others may not be sufficiently distinct to allow specific assignment. Remarkably, roughly 40% of the bones and teeth in this study that were assigned on osteomorphological grounds to *E. hydruntinus* by a subset of the osteologists involved yielded sequences clustering with *E. caballus* (15 of the 36 samples initially determined as *E. hydruntinus*, 9 of the 23 samples subjected to cross-determination). Some of the caballine sequences obtained from these ancient specimens are at present extinct or have not yet been found in modern horses (data not shown). To test whether these could be hybrids between asses and horses, we also analyzed the Y chromosome of some of the specimens. These also carried Y-chromosome sequence of *E. caballus* (data not shown). The discrepancy between a specimen's osteomorphology and genetic signature highlights the underestimation of the past morphological diversity of horses and the limitations of assigning bones to the species level based on expected spatio-temporal distributions rather than characterizing only the subset of bones which have the most diagnostic morphological characters.

In an effort to remove improperly identified samples from the genetic analysis, and thus increase the likelihood of identifying genetic structures underlying true hydruntine populations, samples that were originally determined osteologically to be *E. hydruntinus* were submitted consecutively to several paleontologists/archaeozoologists (A – E) for taxonomic re-classification based on morphology. This was done in a blind test after the first contradictions between genetic and morphological results appeared. A specificity index between zero and one was attributed, "zero" representing no agreement between the various determinations, "one" representing 100% agreement (Table J). This approach was instrumental in reducing the experimental noise of inaccurately determined samples to allow the genetic identification of the hydruntine clades. All of the samples with a specificity index >0.5 were found to belong to the *H1* or *H2* clusters (which we propose as hydruntine), and all caballine sequences recovered were from samples equal or below this value (Figure O). Furthermore, samples originally labelled as

hydruntine which yielded sequences belonging to other clusters (*I* or *CI*), had values of <0.3. One author (SJMD) emphasizes that the pattern of enamel folds in the teeth of *E. hydruntinus* resembles closely that of the African zebras and thus expected specimens assigned to *E. hydruntinus* to cluster with zebras, but this was not the case.

The determinations allowing assignment of the specificity index of the hydruntine bones are presented in Table K.

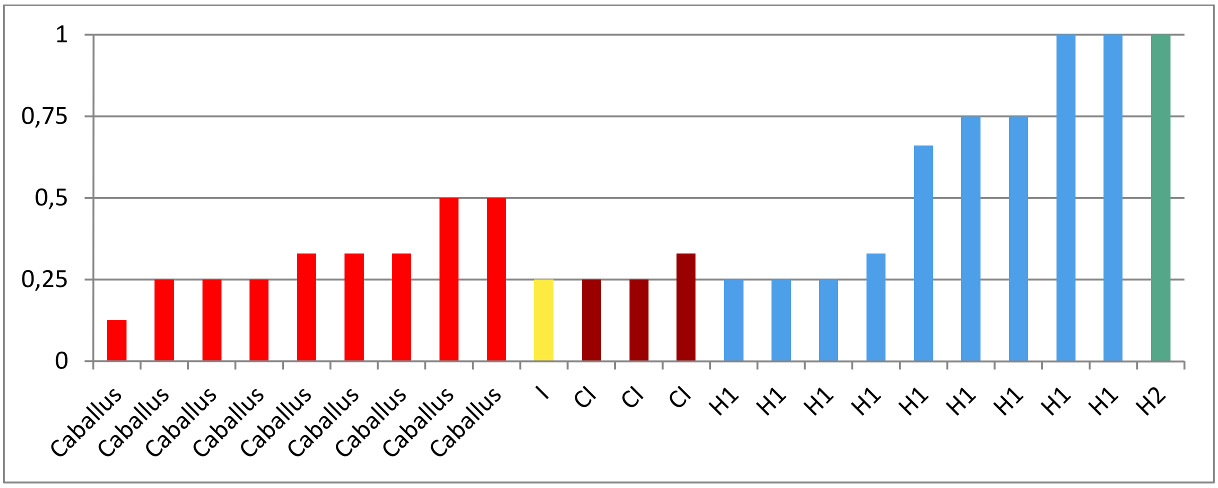


**Figure O: Histogram of specificity index and clade of bone samples considered to be *E. hydruntinus***
y-axis: specificity index, x-axis: results of the genetic analysis.

## IV. Historical specimens

### 1. E. hemippus

A root sample from a tooth (#MNHN C.G. 1977 n°3) extracted from the skeleton preserved in the National Museum of Natural History in Paris was analyzed, coming from the skull of one of the two animals that were offered to the Empress Josephine in 1855. The second specimen can be found in the main exhibition hall of the Gallery of Palaeontology and Comparative Anatomy of the National Museum of Natural History in Paris. Geoffroy St-Hilaire wrote in 1855 about these animals: "*These two wild horses ("chevaux sauvages"), both female and not yet fully adult, were sent by the Viceroy of Egypt under the name of onagers to Her Majesty the Empress*." In a note, he attempts to be more precise about the origin of these animals: "*The Viceroy of Egypt had received these animals from Seraskier Izzet-Pacha, governor of Syria, who in turn had taken them from the Arab chief Atha-Bey. They came originally, we are assured, from the desert of Syria, between Palmyra and Baghdad. This species had not yet been seen either in Egypt or even in Damascus*" [101].

The samples NMW1308/B 4690 (*E. o. hemippus*, Syria, 1892), NMW6048/ST 345 (*E. onager Boddaert*, Syria, 1864, shown in Figure P), and NMW5493/B 3625 (*E. o. hemippus*, desert of Aleppo, Syria, 1929) were provided by the Mammal Collection, National History Museum Vienna, Austria.

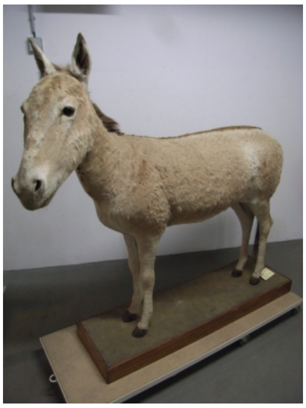

**Figure P: *E. h. hemippus***
NMW 6048/ST 345 (*E. onager Boddaert*, Syria, 1864), mounted specimen from the Natural History Museum, Vienna, Austria. Photo: E.-M. Geigl.

### 2. E. hemionus

The following equid samples originating from the "collection vertebrés supérieurs" of the National Museum of Natural History, Paris, France, were analyzed in the present study:

1. MNHN A2313: A tooth of an *E. hemionus* that entered the collection in 1838 thanks to a donation of Mr. Dussunier, who indicated "Kutch" as the country of origin
2. MNHN A.549: a piece of skin of an *E. hemionus* from the "pays de Kutch"
3. MNHN 1893-509: a tooth of an *E. hemionus* from the 19th century whose origin was indicated as Persia
4. MNHN1902-354: a tooth of an *E. hemionus* of unknown origin from the National Museum of Natural History described as onager from "Abyssinie" donated by the consul of France on August 12, 1851; died in 1867
5. MNHN 1909-308: a piece of cartilage of the ribs and a piece of hoof of an *E. hemionus* that was born in the Paris zoo on 11/6/1895 from hemionus parents (male *E. hemionus* born in the Paris zoo in 1888; the female *E. hemionus* could have been a wild individual caught in Persia and acquired by the "jardin d'acclimatation" in 1884, a female hemione born in the "menagerie" in 1886 or another born in the "ménagerie" in 1891)

## 3. E. kiang

The following equid samples originating from the "collection vertebrés supérieurs" of the National Museum of Natural History, Paris, France, and the Mammal Collection, National History Museum Vienna, Austria, were analyzed in the present study:

1. MNHN1899-610 n°24: a tooth of an *E. kiang* from 1899, a donation of Mr. de Jancourt
2. NMW BH'08: a hoof of an *E. kiang* from 1946 of the Tiergarten Schönbrunn

# V. Samples from present-day animals

## 1. Dziggetais

The mitochondrial hypervariable regions of 18 samples from feces and carcass remains of dziggetais from the Gobi desert in Mongolia were analyzed in the present study. These samples have been described previously and microsatellite markers have been analyzed by [8]. CITES 2007 AT 06 - E – 1888.

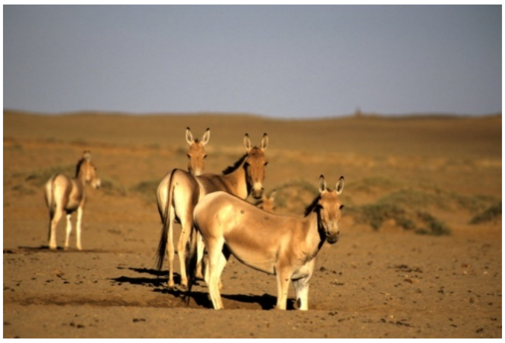

**Figure Q: Dziggetais at a pothole in the Gobi.** (Photo: P. Kaczensky)

## 2. Onagers and kiang from the Sigean zoo

Feces of onagers (*E. hemionus; Jihanne, no. 863*) and hair of a kiang from the Réserve Africaine de Sigean (Sigean, France) were provided by Dr. Elodie Trunet and analyzed in the present study.

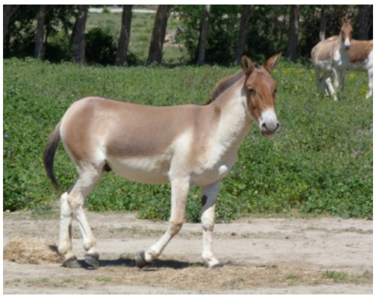

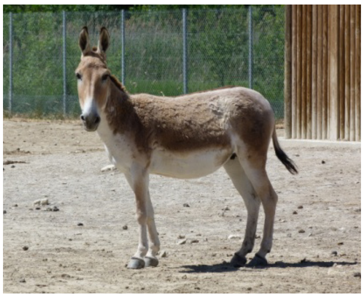

**Figure R: Onager (right) and kiang (left)** (Réserve Africaine de Sigean, France)

## 3. The Asiatic wild ass in Hai-Bar Yotveta breeding core, Israel

*Shirli Bar-David*

The Asiatic wild ass (*E. hemionus*) population in Israel originated from the Israel Nature and Park Authority (INPA) breeding core established at the Hai-Bar Yotvata Reserve, Israel, in 1968. The breeding core was founded from six Persian wild asses (*E. hemionus onager*, 3 males, 3 females), followed by the addition of five animals (2 males, 3 females) from the Turkmenian subspecies (*E. hemionus kulan*). The animals were kept at Hai-Bar and were allowed to interbreed. Hence the herd is considered as *E. hemionus* ssp. [102]. Blood samples were collected in 1989-1991 as part of the INPA management activities toward reintroduction (e.g. e.g. medical treatment, translocations between enclosures) [103], and DNA and blood samples have been preserved since then. We analyzed DNA of 17 of these animals (7 males, 10 females).

http://www.parks.org.il/sites/English/parksandreserves/haibaryotvata/Pages/default.aspx

The genealogy of the animals from which blood samples had been taken has been reconstructed for this study on the basis of the corresponding studbooks from the "Tierpark" Berlin, Germany. The Studbook register of the founder animals from the Hai-Bar-Yotveta Reserve is shown in Table L.

## 4. E. hemionus onager from the Bahram-e Goor reserve (Iran)

*Mahmoud-Reza Hemami*

Dung samples were collected from Qatrouiyeh National Park, the previous core zone of Bahram-e Goor Protected Area. The park is divided into three plains by mountains, namely Einol-Jalal, Deh-Vazir and Rig-Jamshid. The samples were collected opportunistically from the three plains.

## 5. E. hemionus onager from the Khartouran reserve (Iran)

*Marjan Mashkour*

Bones of four onagers from the Natural reserve of Khartouran in the province of Khorassan in Iran that had been collected in 1997 and stored in the University of Teheran, Iran, have been included in the present study.

# VI. References


1. Stamatakis A (2014) RAxML version 8: a tool for phylogenetic analysis and post-analysis of large phylogenies. Bioinformatics 30: 1312-1313.
2. Jonsson H, Schubert M, Seguin-Orlando A, Ginolhac A, Petersen L, et al. (2014) Speciation with gene flow in equids despite extensive chromosomal plasticity. Proc Natl Acad Sci U S A 111: 18655-18660.
3. Vilstrup JT, Seguin-Orlando A, Stiller M, Ginolhac A, Raghavan M, et al. (2013) Mitochondrial phylogenomics of modern and ancient equids. PLoS One 8: e55950.
4. Ryder OA, Chemnick LG (1990) Chromosomal and molecular evolution in Asiatic wild asses. Genetica 83: 67-72.
5. Luo Y, Chen Y, Liu F, Jiang C, Gao Y (2011) Mitochondrial genome sequence of the Tibetan wild ass (Equus kiang). Mitochondrial DNA 22: 6-8.
6. Beja-Pereira A, England PR, Ferrand N, Jordan S, Bakhiet AO, et al. (2004) African origins of the domestic donkey. Science 304: 1781.
7. Rosenbom S, Costa V, Chen S, Khalatbari L, Yusefi GH, et al. (2015) Reassessing the evolutionary history of ass-like equids: insights from patterns of genetic variation in contemporary extant populations. Mol Phylogenet Evol 85: 88-96.
8. Kaczensky P, Kuehn R, Lhagvasuren B, Pietsch S, Yang W, et al. (2011) Connectivity of the Asiatic wild ass population in the Mongolian Gobi. Biological Conservation 144: 920-929.
9. Oakenfull EA, Lim HN, Ryder OA (2000) A survey of equid mitochondrial DNA: implications for the evolution, genetic diversity and conservation of Equus. Cons Genet 1: 345-355.
10. Orlando L, Metcalf JL, Alberdi MT, Telles-Antunes M, Bonjean D, et al. (2009) Revising the recent evolutionary history of equids using ancient DNA. Proc Natl Acad Sci USA 106: 21754-21759.
11. Tournepiche JF (1987) L'occupation épipaléolithique de la grotte du Quéroy, Cne de Chazelles (Charente). Préhistoire de Poitou-Charentes, Problèmes actuels. Paris: C.T.H.S.
12. Peters J (1998) Römische Tierhaltung und Tierzucht. Eine Synthese aus archäozoologischer Untersuchung und schriftlich-bildlicher Überlieferung. . Rahden/Westf.: Leidorf.
13. Majidzadeh Y (2011) Ozbaki Archaeological Excavations. First volume. Art and architecture. Tehran: ICHHTO.
14. Mashkour M, Mohaseb Karimlu AF (2011) Hunting and husbandry in the Ozbaki archaeological Zone (Savojbolagh plain) from the 6th millennium until the Iron Age: Archaeozoological study of Jeiran Tepe, Maral Tepe, Doshan Tepe and Tepe Ozbaki. Tehran. 273- 302 and 597-601 p.
15. Roustaei K, Mashkour M, Tenberg M (in press) Tappeh Sang-e Chakhmaq and Beginning of the Neolithic Period in Northeastern Iran. Antiquity.
16. Masuda S (1994) Tappeh Sanghi- Chakhmaq. Tokyo Rokko.
17. Mashkour M, Mohaseb A (2005) Preliminary Report on Bone Remains of Tepe Deh Kheir, In Hasan Rezvani. Preliminary Report on the 2nd Season of Excavations at Deh Kheir. 18 p.
18. Egami N, Masuda S, Gotoh T (1977) Tal-i-Jarri A: A Preliminary Report of the Excavation in Marv Dasht, 1961 and 1971. Orient 13: 1-14.
19. Nishiaki Y, Mashkour M (2006) The stratigraphy of the Neolithic site of Jari B, Marv Dasht, southwest Iran. Orient-Express Notes et nouvelles d'Archéologie Orientale 3: 77-81.
20. Mashkour M, Mohaseb A, Debue K (2006) Chapter 10 - Towards a specialized subsistence economy in the Marydasht Plain. Preliminary zooarchaeological analysis of Mushki, JariB, Jari A and Bakun A and B. In: Alizadeh, editor. The Origins of State Organizations in Prehistoric Highland Fars Excavations at Tall-e Bakun. Chicago, Illinois: Oriental Institute Publications. pp. 101-105.
21. Biglari F, Javeri M, Mashkour M, Yazdi Y, Shidrang S, et al. Iran Palaeolithic. Test excavations at the Middle Paleolithic sites of Qaleh Bozi, Southwest of Central Iran, A preliminary report. . In: Otte M, Biglari F, Jaubert J, editors. BAR International Series 1968; 2009 Lisbon. pp. 29-38.
22. Negahban EO. Preliminary report of the excavations of Sagzabad; 1976. pp. 247-271.

23. Majidzadeh Y (1981) Sialk III and the Pottery Sequence at Tepe Ghabristan. The Coherence of the Cultures of the Central Iranian Plateau. Journal of Persian Studies 19: 141-147.
24. Dive J, Eisenmann V (1991) Identification and DIscrimination fo First Phalanges from Pleistocene and Modern Equus, WIld and Domestic. In: Uerpmann RHMaHP, editor. Equids in the Ancient World II (Beihefte zum Tübinger Atlas des Vorderen Orients, Reihe A, Nr 19/2). Wiesbaden: Dr. Ludwig Reichert Verlag. pp. 278-333.
25. Eisenmann V, Beckouche S. Identification and discrimination of metapodials from Pleistocene and modern Equus, wild and domestic. In: Meadow RH, Uerpmann HP, editors. Beihefte zum Tübinger Atlas des Vorderen Orients, Reihe A; 1986; Tübingen. Dr. Ludwig Reichert Verlag, Wiesbaden. pp. 117-163.
26. Mashkour M, Fontugne M, Hatte C (1999) Investigation on the evolution of subsistence economy in the Qazvin Plain (Iran) from the Neolithic to the Iron Age. Antiquity 73: 65-76.
27. Mashkour M (2001) Chasse et élevage du Néolithique à l'äge du Fer dans la plaine de Qazvin (Iran). Etude archéozoologique des sites Zagheh, Qabrestan et Sagzabad. Paris: Université de Paris I/Panthéon-Sorbonne.
28. Orlando L, Mashkour M, Burke A, Douady CJ, Eisenmann V, et al. (2006) Geographic distribution of an extinct equid (Equus hydruntinus: Mammalia, Equidae) revealed by morphological and genetical analyses of fossils. Mol Ecol 15: 2083-2093.
29. Majidzadeh Y (1976) The Early Prehistoric Cultures of the Central Plateau of Iran: An Archaeological History of Its Development During the Fifth and Fourth Millennia B.C. Chicago University of Chicago.
30. Eisenmann V, Mashkour M (1999) The Small Equids (Perissodactyla, Mammalia) of the Pleistocene of Binagady (Azerbaijan) and Quazvin (Iran): E. hemionus binagadensis nov. subsp. and E. hydruntinus. Geobios 32: 105-122.
31. Gadzhiev MG, Kohl PL, Magomedov RG, Stronach D, Gadzhiev SL, et al. (2000) Daghestan-American Archaeological Investigations in Daghestan, Russia, 1997-1999. Eurasia Antiqua 6: 47-124.
32. Gadzhiev MG, Kohl PL, Magomedov RG, Stronach D, Morales A, et al. (1997) The 1995 Daghestan-American Velikent Expedition. Excavations in Daghestan, Russia. Eurasia Antiqua 3: 181-222.
33. Areshyan G. Early Bronze Age settlement pattern in the plain of Ararat and its vicinity.; 2004.
34. Uerpmann M, Uerpmann H-P. Zug- und Lasttiere zwischen Maikop und Trialeti. In: Uerpmann M, Uerpmann, H.-P., Zug- und Lasttiere zwischen Maikop und Trialeti, editor; 2006; Berlin. Deutsches Archäologisches Institut Berlin
35. Uerpmann M, Uerpmann H-P (2008) Bronze and Iron Age animal economy at Didi-gora and Tqisbolo-gora (Kachetia, Georgia). Archäologische Mitteilungen aus Iran und Turan 40: 169-264.
36. Peters J, Schmidt K (2004) Animals in the symbolic world of Pre-Pottery Neolithic Göbekli Tepe, southeastern Turkey: a preliminary assessment. . Anthropozoologica 39: 179-218.
37. Schmidt K (2006) Sie bauten die ersten Tempel. München: Beck Verlag.
38. von den Driesch A, Peters J (1999) Vorläufiger Bericht über die archäozoologischen Untersuchungen am Göbekli Tepe und am Gürcütepe bei Urfa, Türkei. Istanbuler Mitteilungen 49: 23-39.
39. Özbaşaran M (2012) Aşıklı. In: Özdoğan M BNKP, editor. The Neolithic in Turkey. Istanbul: Archaeology & Art publications. pp. 135-158.
40. Stiner MC, Buitenhuis H, Duru G, Kuhn SL, Mentzer SM, et al. (2014) A forager-herder trade-off, from broad-spectrum hunting to sheep management at Asikli Hoyuk, Turkey. Proc Natl Acad Sci U S A 111: 8404-8409.
41. Buitenhuis H (1997) Aşikli Höyük: A "Protodomestication" site. Anthropozoologica 25-26: 655-662.
42. Martin L, Russell. N (2006) The equid remains from Neolithic Çatalhoyuk, central Anatolia: A preliminary report. In Horses and humans: the evolution of human-equine relationships. In:

Olsen SJ, editor. British Archaeological Reports International Series 1560. Oxford. pp. 115-126.
43. Davis SJM (1980) Late Pleistocene and Holocene Equid Remains from Israel. Zool J Linnean Soc 70: 289-312.
44. Payne S. Early Holocene Equids from Tall-i-Mushki (Iran) and Can Hasan III (Turkey). In: Meadow RH, Uerpmann HP, editors. Beihefte zum Tübinger Atlas des Vorderen Orients; 1991; Tübingen. Dr. Ludwig Reichert Verlag, Wiesbaden. pp. 132-165.
45. Bonifay M-F (1991) Equus hydruntinus Regalia minor n.ssp. from the Caves of Lunel-Viel (Herault, France). In: Meadow RH, Uerpmann HP, editors. Equids in the Ancient World II (Beihefte zum Tübinger Atlas des Vorderen Orients, Reihe A, Nr 19/2). Wiesbaden: Dr. Ludwig Reichert Verlag. pp. 178-216.
46. Baxter I. Species identification of equids from Western European archaeological deposits: methodologies, techniques and problems. In: Anderson S, editor. Current and Recent Research in Osteoarchaeology; 1998. Oxbow. pp. 3-17.
47. Uerpmann H-P (2006) Betrachtungen zum Verhältnis zwischen Wildpferd (Equus ferus) und Hydruntinus (Equus hydruntinus) im Jungpleistozän und Holozän auf der Iberischen Halbinsel. Munibe (Anthrpologie-Arkeologia) 57: 351-358.
48. Öztan A (2002) Köşk Höyük: Anadolu arkeolojisine yeni katkılar. TÜBA-AR 5: 57-72.
49. Öztan A (2007) Köşk Hoyuk: Nigde--Bor Ovası'nda bir Neolitik yerlesim. In: Ozdogan M, Basgelen N, editors. Turkiye'de Neolitik Donem: Yeni kazılar, yeni bulgular. Istanbul: Arkeoloji ve Sanat Yayınları:. pp. 223-236.
50. Öztan A. Archaeological investigations at Köşk Höyük, Nigde. In: d'Alfonso L, Balza ME, Mora C, editors; 2008; Pavia. Italian University Press. pp. 83-96.
51. Silistreli U (1985) Kösk Höyügü. Kazı Sonuçları Toplantısı 7: 129-141.
52. Silistreli U (1989) Les fouilles de Kösk Höyük. In: Emre K, Mellink M, Hrouda B, Özgüç N, editors. Anatolia and the ancient Near East: Studies in honor of Tahsin Özgüç. Ankara. pp. 461-463.
53. Arbuckle BS, Öztan A, S. Gulçur S (2009) The evolution of sheep and goat husbandry in central Anatolia. . Anthropozoologica 44: 129-157.
54. Boessneck J, von den Driesch A (1976) Pferde im 4./3. Jahrtausend v. Chr. in Ostanatolien. Säugetierkundliche Mitteilungen - Sonderdruck 24: 81-87.
55. Özgüç N (1979) Some contributions to early Anatolian art from Acemhöyük. Belleten 43: 288-310.
56. Öztan A (1995) 1994 Acemhöyük kazıları. Kazı Sonuçları Toplantısı 17: 209-213.
57. Özgüç N (1980) Seal impressions from the palaces at Acemhöyük. In: Porada E, editor. Ancient art in seals,. Princeton: Princeton University Press. pp. 61-99.
58. Joukowsky MS (1996) Early Turkey: An Introduction to the archaeology of Anatolia from prehistory through the Lydian period. Dublin: Kendall/Hunt.
59. Uerpmann M (2006) Von Adler bis Zahnbrassen - Der Beitrag der Archäozoologie zur Erforschung Troias. In: Korfmann M, editor. Troia Archäologie eines Siedlungshügels und seiner Landschaft. Mainz: Philipp von Zabern. pp. 283-296.
60. Kussinger S (1988) Tierknochenfunde vom Lidar Höyük in Südostanatolien (Grabungen 1979-86). Munich: Ludwig Maximilians University.
61. Boessneck J, Driesch Avd (1986) Tierknochen und Molluskenfunde aus Munbāqa. Mitteilungen der Deutschen Orient-Gesellschaft 118: 147-160.
62. Boessneck J, Peters J (1988) Tierknochen- und Molluskenfunde aus dem Grabungsbereich 'Kuppe' in Tall Munbāqa. Mitteilungen der Deutschen Orient-Gesellschaft 120: 51-58.
63. Eisenmann V (1985) Découverte d'un crâne non répertorié d'hémippe Equus hemionus hemippus (Perissodactyla, Equidae). Mammalia 49.
64. Uerpmann HP (1986) Halafian Equid Remains form Shams ed-Din Tannira in Northern Syria. In: Meadow RH, Uerpmann HP, editors. Equids in the Ancient World (Beihefte zum Tübinger Atlas des Vorderen Orients, Reihe A, Nr 19/1). Wiesbaden: Dr. Ludwig Reichert Verlag. pp. 246-265.

65. Vila E (1994) L'exploitation des animaux en Mésopotamie aux IVe et IIIe millénnaire av. JC; 21 MdC, editor. Paris: CNRS Editions.
66. Zohary M (1973) Geobotanical Foundations of the Middle East; Fischer G, editor. Stuttgart: Swets & Zeitlinger / Amsterdam
67. Voinea V, Dobrinescu C (2003) Aşezarea Hamangia III de la Cheia. Campaniile 2001-2002 (Raport preliminar). 9-22 p.
68. Bălăşescu A, Radu V (2003) Studiul arheozoologic preliminar al materialului faunistic de la Cheia (jud. Constanţa). Campania 2001. . Pontica 35-36: 25-32.
69. Bălăşescu A, Radu V, Moise D (2005) Omul si mediul animal intre mileniile VII-IV i.e.n. la Dunarea de Jos. Biblioteca Muzeului Naţional. Bucuresti: Editura Cetatea de Scaun. pp. 404
70. Bălăşescu A, Radu V (2004) Oameni şi animale. Strategii şi resurse la comunităţile preistorice Hamangia şi Boian. In Biblioteca Muzeului Naţional. Bucuresti: Editura Cetatea de Scaun. pp. 310
71. Bălăşescu A (2008) Consideraţii asupra exploatării mamiferelor de la Cheia (cultura Hamangia),. Pontica XLI: 49-55.
72. Eisenmann V (1986) Comparative Osteology of Modern and Fossil Horses, Half-asses, and Asses. In: Meadow RH, Uerpmann HP, editors. Equids in the Ancient World (Beihefte zum Tübinger Atlas des Vorderen Orients, Reihe A, Nr 19/1). Wiesbaden: Dr. Ludwig Reichert Verlag. pp. 67-116.
73. Necrasov O, Haimovici S (1959) Sur la présence d'une espèce Pléistocène d'Équidés Equus hydruntinus. Reg. dans le Néolithique Roumain,.
74. Necrasov O, Haimovici S (1960) Nouvelle contribution a l'etude de Equus (Asinus) hydruntinus Reg.
75. Eisenmann V, G.F. B (1995) Equus cf taubachensis et E.hydruntinus de la Grotte de Prolom 2 (Crimee, Ukraine). Bulletin du Museum national d'Histoire naturelle 4 serie 16: 329-347.
76. Burke A, Eisenmann V, Ambler G (2003) The systematic position of Equus hydruntinus, an extinct species of Pleistocene equid. Quat Res 59: 459-469.
77. Nmejcova-Pavukova N, Nmejcova-Pavukova V. Siedlung und Kreisgrabenanlage der Lengyel-Kultur in Svodin (Südslowakei). In: Wien AIdsAdWiNuIfU-uFdU, editor; 1986; Nové-Vozokany. Nitra Wien. pp. 177-183.
78. Clason AT (1979) The Farmers of Gomolava in the Vinca and la Tène Period. Palaeohistoria 21: 42-81.
79. Jungels C (2009) La grotte de la Bètche-aux-Rotches à Spy. In: Di Modica K, Jungels C, editors. Paléolithique moyen en Wallonie. pp. 188-201.
80. Rucquoy A (1886-1887) Note sur les fouilles faites en Août 1879 dans la caverne de la Bèche-aux-Roches, près de Spy. Bulletin de la Société d'Anthropologie de Bruxelles 5: 318-328.
81. Fraipont J, Lohest M (1886) La race humaine de Néanderthal ou de Canstadt, en Belgique. . Bulletin de l'Académie royale de Belgique 3ème série 12 741-784.
82. Rougier H, Crevecoeur I, Fiers E, Hauzeur A, Germonpré M, et al. (2004) Collections de la Grotte de Spy: (re)découvertes et inventaire anthropologique. Notae Praehistoricae 24: 181-190.
83. De Loe A, Rahir E (1911) Nouvelles fouilles à Spy. Grotte de la Betche-aux-Rotches. . Bulletin de la Société d'Anthropologie de Bruxelles 30 40-58.
84. Semal P, Rougier H, Crevecoeur I, Jungels C, Flas D, et al. (2009) New Data on the Late Neandertals: Direct Dating of the Belgian Spy Fossils. American Journal of Physical Anthropology, 138: 421-428.
85. Germonpré M (2014) The fossil mammals of Spy. In: Semal P, Hauzeur A, editors. Spy cave – State of 120 years of plurisdisciplinary research on the Betche-aux-Rotches from Spy (Jemeppe-sur-Sambre, Province of Namur, Belgium): Royal Belgian Institute of Natural Sciences.
86. Germonpré M, Bogemans F, Van Neer W, Grün R (1993) The dating of two pleistocene mammal assemblages from the Flemish Valley, Belgium. . Contributions Tertiary and Quaternary Geology 30: 147-153.

87. Semal P, Hauzeur A, Higham T, Van der Plicht J, Jungels C, et al. (2014) Dating. In: Semal P, Hauzeur A, editors. Spy cave – State of 120 years of plurisdisciplinary research on the Betche-aux-Rotches from Spy (Jemeppe-sur-Sambre, Province of Namur, Belgium) Brussels: Royal Belgian Institute of Natural Sciences.
88. Stehlin HG, Graziosi P (1935) Ricerche sugli Asinidi fossili d'Europea. Mémoires de la Société Paléontologique Suisse 56: 1-73.
89. Boulbes N (2009) Etude comparée de la denture d'Equus hydruntinus (Mammalia, Perissodactyla) dans le Sud-Est de la France. Implications biogéographiques et biostratigraphiques. Quaternaire 20: 449-465.
90. Delagnes A, Tournepiche JF, Armand D (1999) Le gisement Pléistocène moyen et supérieur d'Artenac (Saint-Mary, Charente): premier bilan interdisciplinaire. Bulletin de la Société Préhistorique Française 96: 469-496.
91. Tournepiche JF (1996) Les grands mammifères pléistocènes de Poitou-Charentes. Paléo 8: 109-141.
92. Simpson GG (1941) Pleistocene felines of North America. AmMus Novitates 1136: 1-27.
93. Hinguant S, Colleter R, Arellano A, Moullé P-E, Pigeaud R (2007) Le cône d'éboulis Pléistocène de la grotte de la Chèvre. Rapport de diagnostic archéologique. Rennes: CNRS UMR 6566 "Civilisations atlantiques et archéosciences".
94. Hinguant S, Colleter R, Arellano A, Biard M, Chaut J-J, et al. (2007) Le Solutréen de la grotte Rochefort (Saint-Pierre-sur-Evre, Mayenne): premiers résultats. Rapport de fin d'opération triennale. Rennes: CNRS UMR 6566 "Civilisations atlantiques et archéosciences".
95. Hinguant S, Moullé P-E, Arellano A (2005) Premiers indices de la présence d'une faune du Pléistocène moyen dans la vallée de l'Erve (Mayenne). Bulletin du Musée d'Anthropologie Préhistorique de Monaco 45: 25-30.
96. Hinguant S, Moullé P-E, Arellano AB (2012) Nouvelles données sur la faune du Pléistocène moyen de la vallée de l'Erve (Mayenne, France). Bulletin du Musée d'Anthropologie Préhistorique de Monaco 52: 35-42.
97. Alberdi MT, Caloi L, Palombo M-R (1988) The Quaternary fauna of Venosa : equids. Bull Mus Anthropol Préhist Monaco 31: 5-39.
98. Boulbes N (2010) Le cheval de Romain-la-Roche, Equus achenheimensis (Mammalia, Perissodactyla). Contribution à la biochronologie des équidés caballins au Pléistocène moyen. Revue de Paléobiologie, Genève 29 747-770.
99. Morales A. Una Fauna Albacetense de la Edad del Bronce: La Morra del Quintanar (Munera). 1991; Albacete. Instituto Estudios Albacetenses. . pp. 145-153.
100. Cardoso JL, Vilstrup JT, Eisenmann V, Orlando L (2013) First evidence of Equus asinus L. in the Chalcolithic disputes the Phoenicians as the first to introduce donkeys into the Iberian Peninsula. . Journal of Archaeological Science 40: 4483-4490.
101. Geoffrey-Saint-Hilaire I (1855) Sur deux Chevaux sauvages, d'une espèce nouvelle (Equus hemippus), donnés par S.M. l'Impératrice à la Ménagerie du Muséum d'Histoire Naturelle. Comptes Rendus de l'Académie des Sciences Paris 41: 1220-1224.
102. Saltz D, D. I. Rubenstein DI (1995) Population-dynamics of a reintroduced Asiatic wild ass (Equus-hemionus) herd. Ecological Applications 5: 327-335.
103. Sinai Y (1994) A program for selecting individuals from a breeding core for reintroduction - Equus hemionus in Hai-Bar Yotveta. Jerusalem: The Hebrew University.
